# Chromatin condensates tune nuclear mechanosensing and preserve nuclear integrity to prevent cGAS activation

**DOI:** 10.1038/s44318-026-00884-z

**Published:** 2026-08-12

**Authors:** Sarah D’Annunzio, Martina Di Santo, Giulia Vitali, Lucia Santomaso, Daniela Michelatti, Leonardo Morelli, Chiara Bernardis, Maulana Ariefai, Shiza Nasir, Sara Lago, Lorenzo Lunelli, Claudia Testi, Emanuele Pontecorvo, Alessandro Poli, Fabrizio A Pennacchio, Paolo Maiuri, Elodie Sanchez, David Genevieve, Lorenzo Petrolli, Thomas Tarenzi, Roberto Menichetti, Raffaello Potestio, Giancarlo Ruocco, Alessio Zippo

**Affiliations:** 1 Department of Cellular, Computational and Integrative Biology (CIBIO), University of Trento, 38123 Trento, Italy; 2 Sensors and Devices Center, Fondazione Bruno Kessler, Trento, Italy; 3 Center for Life Nano- and Neuro-Science, Istituto Italiano di Tecnologia, Rome, Italy; 4 CrestOptics S.p.A., Rome, Italy; 5 IFOM ETS The AIRC Institute of Molecular Oncology, Milan, Italy; 6 Department for Health Sciences and Technology ETH, Zürich, Switzerland; 7 Department of Molecular Medicine and Medical Biotechnology, Università degli Studi di Napoli “Federico II”, Naples, Italy; 8 Montpellier University, Inserm U1183, Montpellier, France; 9 Reference center for Rare Disease developmental anomaly, Clinical genetic Department, Montpellier University Hospital, Montpellier, France; 10 Physics Department, University of Trento, Trento, Italy; 11 INFN-TIFPA, Trento Institute for Fundamental Physics and Applications, Trento, Italy; 12 Dipartimento di Fisica, Università di Roma “La Sapienza”, Roma, Italy

**Keywords:** Chromatin, Transcription & Genomics, Molecular Biology of Disease

## Abstract

Cells and tissue integrity are constantly challenged by the necessity to adapt and respond to mechanical loads. Among cellular components, the nucleus possesses mechano-sensing and mechanotransduction capabilities, yet the molecular mechanisms involved remain poorly defined. Here we investigate whether the mechanical properties of chromatin and its organization into condensates contribute to nuclear adaptation to external forces, while preserving its integrity. By interrogating the effects of MLL4 loss-of-function in Kabuki Syndrome, we find that the balancing of transcriptional and Polycomb condensates tunes nuclear responsiveness to external mechanical forces. MLL4 assembles into mechanosensitive condensates through its prion-like domain, and this response is regulated by the chromatin context. Furthermore, the mechano-sensing activity of MLL4 condensates is instrumental to withstand the physical challenges nuclei experience during cell confinement and migration by preserving their integrity. In Kabuki Syndrome, persistent nuclear envelope rupture triggers cGAS-STING activation, leading to programmed cell death. Together, these findings identify chromatin condensates as active regulators of nuclear mechanosensing and establish a mechanistic link between defective chromatin organization and cGAS–STING activation in Kabuki syndrome.

## Synopsis

Besides contributing to genome function, chromatin condensates are active components of the nuclear mechano-response. This study shows that the histone methyltransferase MLL4 functions as a chromatin mechano-sensor that preserves nuclear integrity and restrains cGAS-STING signalling during mechanical stress.

- Mechanical forces shift the dynamic balance between transcriptional and Polycomb condensates to tune nuclear mechanical properties.
- MLL4 assembles into mechano-responsive condensates through its prion-like domain, acting as a chromatin mechanosensor.
- MLL4-dependent condensate dynamics preserve nuclear integrity during mechanical stress.
- MLL4 haploinsufficiency in Kabuki Syndrome compromises the nuclear adaptation to mechanical stress, increasing nuclear fragility.
- Persistent nuclear envelope rupture activates cGAS-STING signalling, linking defective chromatin mechano-responsiveness to Kabuki syndrome pathology

## Abstract (web summary)

Mechanical stress remodels chromatin condensates through MLL4 to preserve nuclear integrity and suppress cGAS–STING activation.

## Graphical abstract

**Figure:**
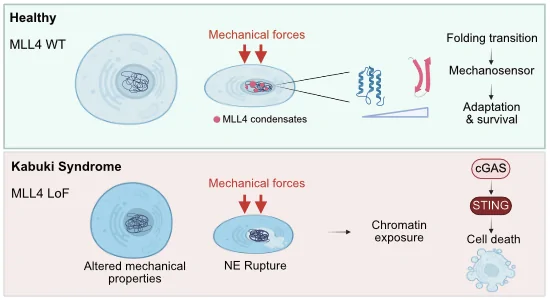

## Introduction

Tissue functionality and homeostasis rely on the capability of cells to respond and adapt to both biochemical and mechanical environmental stimuli. The extracellular matrix (ECM) generates extrinsic mechanical forces which are transmitted and sensed by the cells through structural components and organelles, triggering different cellular responses across time and space (Iskratsch et al, 2014; Kechagia et al, 2019). How biological systems encode and decode mechanical forces depends on the inherent physical properties of the cellular components, with the nucleus being constantly exposed to inward and outward forces that can lead to its transient deformations while preserving the genome integrity (Bustin and Misteli, 2016).

ECM-mediated forces are directly transmitted through the cytoskeleton and LINC complex to the nuclear envelope (NE) and the nuclear lamina, ultimately propagating the mechanical load to the chromatin. The nuclear lamina is a meshwork of the intermediate filament proteins Lamin A/C and B, whose relative abundance scales with the stiffness of the ECM (Dechat et al, 2008; Swift et al, 2013). Nuclear mechanics is determined by the physical properties of the nuclear lamina and the chromatin, which together modulate the magnitude and the timing of mechano-responses (Miroshnikova and Wickström, 2022; Stephens et al, 2017; Lammerding et al, 2006; Furusawa et al, 2015; Fasciani et al, 2020). As chromatin behaves as a dynamic viscoelastic polymer in response to nuclear deformations, its organization is temporally reshaped with an increment of euchromatin (H3 acetylation and H3K4 methylation) paralleled by a decrease of constitutive heterochromatin (H3K9me3), dissipating mechanical energy and safeguard the genome integrity (Nava et al, 2020; Killaars et al, 2019; Killaars et al, 2020; Heo et al, 2023). Albeit this evidence, the precise contribution of chromatin organization to nuclear mechanical resilience remains incompletely understood.

Maintaining nuclear integrity relies on the integration of multiple mechanical inputs, including cytoskeleton-generated compressive and tensile forces acting on the nucleus, together with contributions from intranuclear processes and chromatin biophysical properties. During cell migration through confined environments, nuclear deformation can lead to transient and localized NE rupture (Kalukula et al, 2022; Raab et al, 2016; Denais et al, 2016), triggering the recruitment of chromatin-binding protein BAF, which senses exposed genomic DNA and initiates NE repair through ESCRT-III, Lamins, and LEM-domain proteins (Denais et al, 2016; Maciejowski and Hatch, 2020; Halfmann et al, 2019; Kono et al, 2022; Young et al, 2020). Persistent NE rupture, which is often linked to pathological conditions compromising Lamin A/C abundance or function (Denais et al, 2016; Lammerding et al, 2004; Goldman et al, 2004; De Vos et al, 2011; Karoutas et al, 2019; Bell et al, 2022), leads to DNA damage and activation of cGAS-STING pathway, a cytosolic DNA sensing mechanism that triggers innate immune responses (Young et al, 2020; Denais et al, 2016,2021; Guey et al, 2020). In many physiological and pathological conditions, the recurrent loss of NE integrity initiates the cGAS-STING cascade, which leads to diverse outcomes including type I interferon response, inflammasome activation, and cell death pathways (Hopfner and Hornung, 2020; Frittoli et al, 2023; Sladitschek-Martens et al, 2022). Yet, how chromatin mechanics and nuclear integrity shape the downstream cGAS-STING response remains poorly defined.

Perturbations of mechano-transmission and mechano-responses underline several pathological conditions including cancer, fibrosis, cardiomyopathies, and developmental disorders (Zuela-Sopilniak and Lammerding, 2022; Negri et al, 2023; Banani et al, 2022). Among them, Kabuki syndrome (KS) is a rare genetic disease caused by the haploinsufficiency of the chromatin regulators MLL4 (encoded by *KMT2D*) or UTX (encoded by *KMD6A*) and is characterized by a broad spectrum of clinical features, including growth retardation, craniofacial abnormalities, and skeletal defects (Bögershausen and Wollnik, 2013; Miyake et al, 2013; Boniel et al, 2021). These phenotypes have been primarily linked to altered transcriptional and epigenetic regulation. However, emerging evidence suggests that defects in cellular responses to mechanical cues may also contribute to aspects of the disease, particularly in the context of development and tissue homeostasis (Bögershausen and Wollnik, 2013; Miyake et al, 2013; Boniel et al, 2021). Both MLL4 and UTX harbor intrinsically disorder regions (IDRs) that mediate their compartmentalization into transcriptional condensates, which counterbalance the repressive function of the Polycomb (PcG) complexes (Fasciani et al, 2020; Zuela-Sopilniak and Lammerding, 2022; Shi et al, 2021). Besides determining the local chromatin environment to regulate gene expression, condensates are also involved in defining the 3D genome organization and the chromatin’s mechanical properties. In this respect, we have previously found that in KS, MLL4 haploinsufficiency alters the equilibrium between transcriptional and PcG condensates, affecting the nuclear architecture and mechanics, and cell lineage commitment (Fasciani et al, 2020; Negri et al, 2023). Although these findings point to a structural role for chromatin condensates, the mechanism by which chromatin senses and responds to mechanical force remains elusive.

We hypothesize that chromatin condensates contribute to nuclear mechano-sensing and mechano-responsiveness. We investigated whether externally applied forces and nuclear deformation can be sensed and decoded by chromatin condensates through their transient spatial reorganization, thereby regulating the nuclear architecture. To verify this hypothesis, we measured the response of transcription- and PcG-associated chromatin condensates to external mechanical cues in the context of KS. We show that MLL4 promotes mechano-responsiveness by mediating transcriptional condensate nucleation and limiting PcG clustering. The rebalancing of condensates contributes to chromatin softening, preserves nuclear integrity, and prevents aberrant cGAS-STING pathway activation. These findings define an unrecognized function for MLL4 and chromatin condensates in nuclear mechano-responsiveness and nuclear integrity, providing new insight into how chromatin organization contributes to cell fate and pathology in mechanically active tissues.

## Results

### Mechanical stimuli unbalance the transcription- and PcG-associated chromatin condensates

**Figure EV1:**
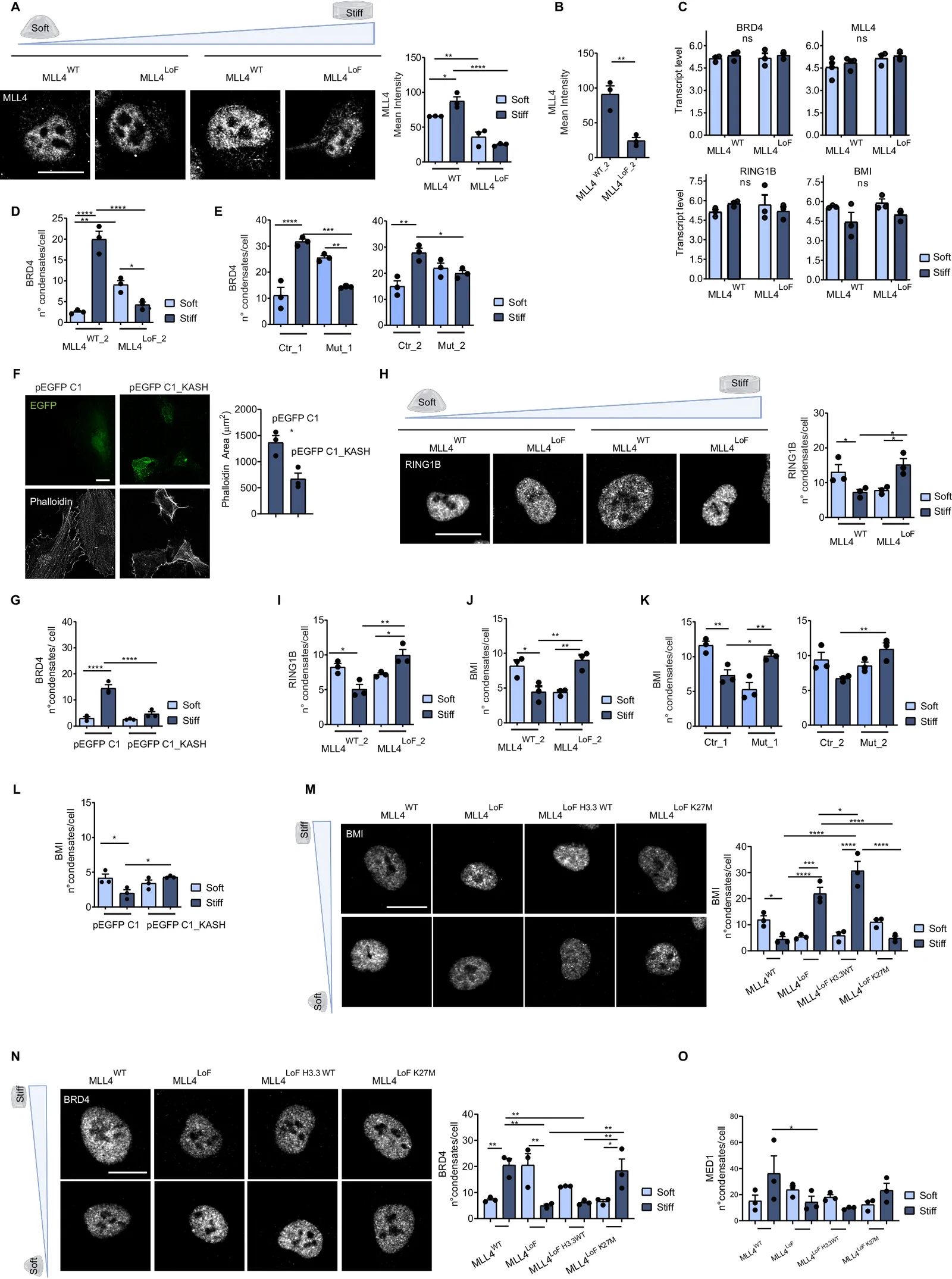
Substrate stiffness unbalances the transcription- and PcG-associated chromatin condensates. (**A**) Representative confocal images and relative quantification of nuclear MLL4 mean intensity in MLL4^WT^ and MLL4^LoF^ MSCs on soft and stiff matrix. Scale bars, 15 µm. Bar plots show mean + SEM of *n* = 3 independent biological replicates. Statistical significance was determined by Sidak’s multiple comparison test (two-way ANOVA). (**B**) Quantifications of nuclear MLL4 mean intensity in MLL4^WT_2^ and MLL4^LoF_2^ MSCs, independent clones on stiff matrix. Bar plots show mean + SEM of *n* = 3 independent biological replicates. Statistical significance was determined by a two-tailed unpaired Student’s *t*-test. (**C**) Real-time quantitative reverse transcription PCR of the indicated genes in MLL4^WT^ and MLL4^LoF^ MSCs on soft and stiff matrix (mean + SEM; *n* = 3 independent experiments each). (**D**) Condensates quantifications of immunostaining for BRD4 in MLL4^WT_2^ and MLL4^LoF_2^ MSCs, independent clones on soft and stiff matrix. Bar plots show mean + SEM of *n* = 3 independent biological replicates. Statistical significance was determined by Sidak’s multiple comparison test (two-way ANOVA). (**E**) Condensates quantifications of immunostaining for BRD4 in primary fibroblasts from healthy donor (Ctrl) or Kabuki patients (Mut) on soft and stiff matrix. Bar plots show mean + SEM of *n* = 3 independent biological replicates. Statistical significance was determined by Sidak’s multiple comparison test (two-way ANOVA). (**F**) Representative confocal images of EGFP signal and Phalloidin staining in MLL4^WT^ MSCs expressing pEGFP C1 or pEGFP C1_DN_KASH construct. On the right, quantification of the Phalloidin Area. Bar plots show mean + SEM of *n* = 3 independent biological replicates. Statistical significance was determined by a two-tailed unpaired Student’s *t*-test. (**G**) Quantifications of nuclear condensates for BRD4 in MLL4^WT^ MSCs expressing pEGFP C1 or pEGFP C1_DN_KASH construct on soft and stiff matrix. Bar plots show mean + SEM of *n* = 3 independent biological replicates. Statistical significance was determined by Sidak’s multiple comparison test (two-way ANOVA). Representative confocal images and relative quantifications of condensates for RING1B in MLL4^WT^/ MLL4^LoF^ (**H**) and MLL4^WT_2^/ MLL4^LoF_2^ independent clones (**I**) MSCs on soft and stiff matrix. Scale bars, 15 µm. Bar plots show mean + SEM of *n* = 3 independent biological replicates. Statistical significance was determined by Sidak’s multiple comparison test (two-way ANOVA). (**J**) Quantifications of immunostaining for BMI nuclear condensates in MLL4^WT_2^ and MLL4^LoF_2^ MSCs, independent clones on soft and stiff matrix. Bar plots show mean + SEM of *n* = 3 independent biological replicates. Statistical significance was determined by Sidak’s multiple comparison test (two-way ANOVA). (**K**) Quantifications of nuclear BMI condensates in primary fibroblasts from healthy donors (Ctrl) or Kabuki patients (Mut) on soft and stiff matrix. (**L**) Quantifications of nuclear condensates for BMI in MLL4^WT^ MSCs expressing pEGFP C1 or pEGFP C1_DN_KASH construct on soft and stiff matrix. Bar plots show mean + SEM of *n* = 3 independent biological replicates. Statistical significance was determined by Sidak’s multiple comparison test (two-way ANOVA). (**M**–**O**) Representative images and relative quantifications of nuclear condensates for BMI (**M**), BRD4 (**N**), and MED1 (**O**) in MLL4^WT^ and MLL4^LoF^ MSCs, as well as MLL4^LoF^ MSCs expressing either H3.3WT or H3.3K27M on soft and stiff matrix. Bar plots show mean + SEM of *n* = 3 independent biological replicates. Scale bars, 15 µm. Statistical significance was determined by Sidak’s multiple comparison test (two-way ANOVA). *P* values definition: *p < 0.05, **p < 0.01, ***p < 0.001, ****p < 0.0001, ns not significant (*p* ≥ 0.05).

**Figure 1:**
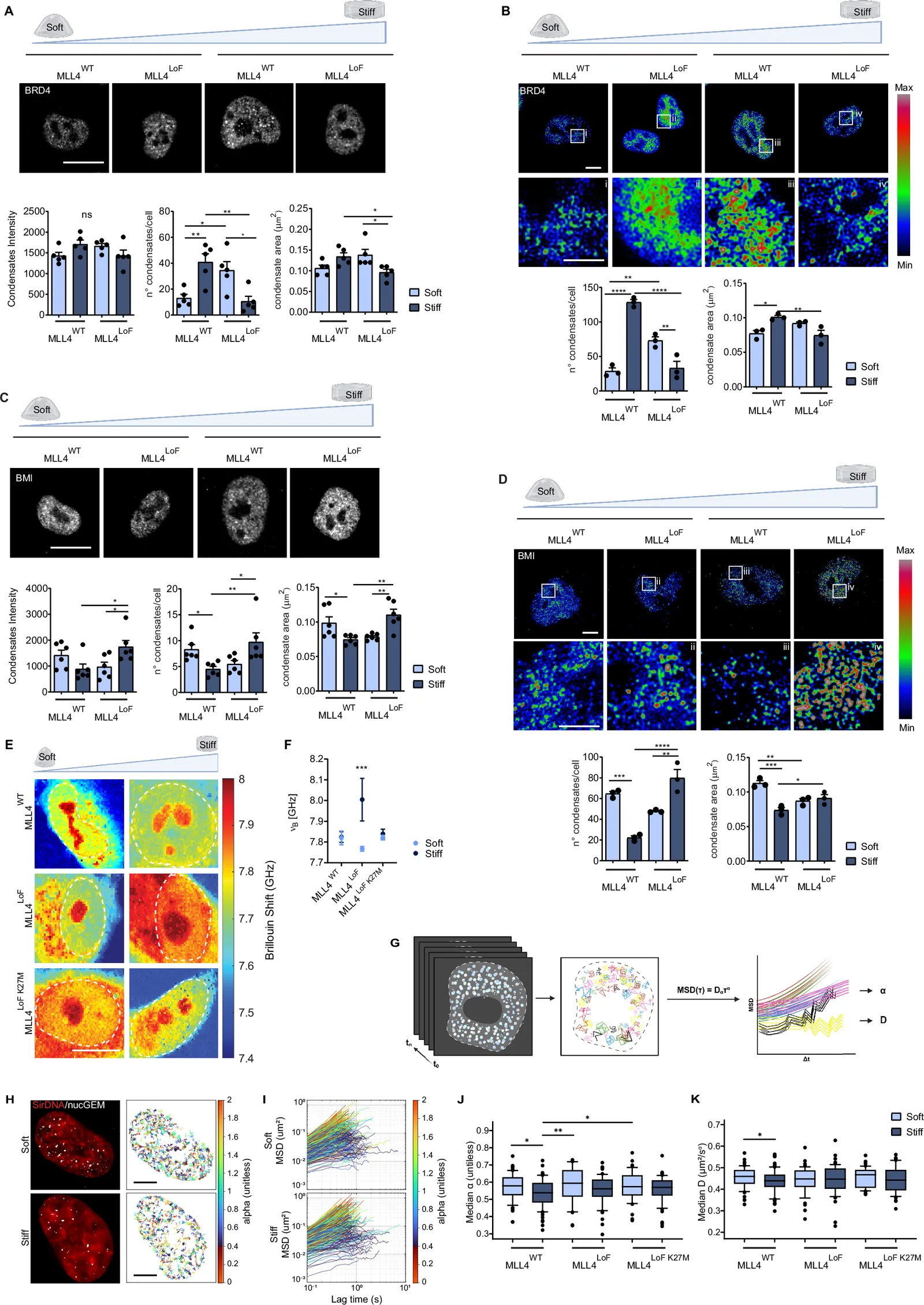
Mechanical stimuli unbalance the transcription- and PcG-associated chromatin condensates. (**A**) Representative confocal images and relative condensates quantifications of immunostaining for BRD4 in MLL4^WT^ and MLL4^LoF^ MSCs on soft (0.5kPA) and stiff (32kPA) matrix. Scale bars, 10 µm. Bar plots show mean + SEM of *n* = 5 independent biological replicates. Statistical significance was determined by Sidak’s multiple comparison test (two-way ANOVA). (**B**) Representative SIM images and quantifications of the number and area of BRD4 condensates in MLL4^WT^ and MLL4^LoF^ MSCs, on soft and stiff matrix, scale bars 5 µm. Bar plots show mean + SEM of *n* = 3 independent biological replicates. Statistical significance was determined by Sidak’s multiple comparison test (two-way ANOVA). Crops of BRD4 condensates are highlighted (i–iv), scale bars 2.5 µm. (**C**) Representative confocal images and relative condensates quantifications of immunostaining for BMI in MLL4^WT^ and MLL4^LoF^ MSCs on soft and stiff matrix, scale bars 10 µm. Bar plots show mean + SEM of *n* = 6 independent biological replicates. Statistical significance was determined by Sidak’s multiple comparison test (two-way ANOVA). (**D**) Representative SIM images and quantifications of the number and area of BMI condensates in MLL4^WT^ and MLL4^LoF^ MSCs, on soft and stiff matrix, scale bars 5 µm. Bar plots show mean + SEM of *n* = 3 independent biological replicates. Statistical significance was determined by Sidak’s multiple comparison test (two-way ANOVA). Crops of BMI condensates are highlighted (i–iv), scale bars 2.5 µm. (**E**) Representative maps of the stiffness distribution in MLL4^WT^ and MLL4^LoF^ MSCs, as well as MLL4^LoF^ MSCs expressing H3.3K27M, acquired with the Brillouin microscope. A higher Brillouin shift in the GHz frequency corresponds to a stiffer region of the cell. The dashed white lines enclose nuclei. Scale bars, 10 µm (**F**) Scatter plot representing the nuclear Brillouin shift in WT and MLL4^LoF^, showing mean + SEM of *n* ≥ 4 independent biological replicates. Statistical significance was determined by a linear mixed-effects model. (**G**) Schematic workflow of nucGEM particle detection, single-particle tracking, and mean squared displacement (MSD) analysis. (**H**) Representative images of nuclei stained with SiR-DNA (red), showing nucGEM particles and reconstructed nucGEM trajectories retrieved in MSCs MLL4^WT^ grown on soft and stiff matrices. (**I**) Distribution of nucGEM tracks in a nucleus of MLL4^WT^ according to anomalous exponent α. (**J**,** K**) Quantification of the generalized diffusion coefficient *D* (**J**) and anomalous exponent α (**K**) in MLL4^WT^, MLL4^LoF^, and MLL4^LoF^ MSCs. grown on soft and stiff matrixes. Box plots show the median and interquartile range; whiskers indicate the 10th–90th percentiles. Each point represents one nucleus from *N* = 5 independent biological replicates. Number of nuclei analyzed: MLL4^WT^ stiff, *n* = 80; MLL4^WT^ soft, *n* = 66; MLL4^Lof^ stiff, *n* = 56; MLL4^Lof^ soft, *n* = 47; MLL4^LoF K27M^ stiff, *n* = 54; MLL4^LoF K27M^ soft, *n* = 41. Statistical significance was determined using a two-sided Mann–Whitney test. *P* values definition: *p < 0.05, **p < 0.01, ***p < 0.001, ****p < 0.0001, ns not significant (*p* ≥ 0.05). Source data are available online for this figure.

To address the contribution of chromatin-binding proteins participating in nuclear condensates to external mechanical stimuli, we monitored the responses of mesenchymal stem cells (MSCs) to the increase in the ECM substrate stiffness in the genetic context of KS. Indeed, we adopted the disease model system in which the insertion of a *KMT2D* truncating mutation (MLL4^Q4092X^) causes MLL4 loss-of-function (LoF), impairing nuclear architecture and chromatin condensates, balancing commitment (Fasciani et al, 2020). Single-cell analyses by quantitative immunofluorescence showed that, irrespective of the substrate stiffness, *KMT2D* haploinsufficiency caused a reduction of MLL4 protein abundance yet maintaining the puncta-like nuclear pattern and its cellular localization (Fig. EV1A–C; Appendix Figs. S1 and S2) commitment (Fasciani et al, 2020). By analyzing the distribution of the transcription-associated cofactors, we found that the increase in substrate stiffness corresponded to an augmented numerosity and size of BRD4 condensates, while its transcript abundance was unchanged (Figs. 1A and EV1C). Importantly, this correlation between BRD4 condensates and substrate stiffness was not observed upon MLL4 LoF. Furthermore, this pattern was replicated using an independent MSC clone carrying a different truncating mutation of MLL4 (encoding MLL4^P4093X^), as well as in KS patient-derived fibroblasts (Figs. 1A and EV1D,E). To rule out whether the cytoskeleton-transmitted mechanical forces control the organization of transcription condensates in dependency of MLL4 abundance, we performed super-resolution imaging by SIM microscopy on the MSCs maintained on a soft and stiff substrate, respectively. Super-resolution imaging revealed that BRD4 is distributed throughout the nucleus in condensates ranging from ∼0.03 to ∼0.75 μm^2^. Notably, MLL4 LoF reduced BRD4 clustering, resulting in fewer and smaller transcriptional condensates in response to substrate stiffness (Fig. 1B). The mechano-responsiveness of transcriptional condensates was corroborated by interfering with the assembly of a functional LINC complex through the overexpression of the dominant negative GFP–Nesprin-1/2–KASH protein, uncoupling the transmission of cytoskeletal forces to the nucleus (Fig. EV1F,G). These results suggest that the increment of the external mechanical forces is associated with an augmented MLL4-dependent assembly of transcriptional condensates.

Considering that MLL4 LoF affected the clustering of PcG complexes into condensates (Fasciani et al, 2020), we determined whether these repressive compartments were similarly responsive to the mechanical cues. Quantitative immunofluorescence analyses showed that although the transcription levels were unaltered (Fig. EV1C), condensates comprising PRC1 components BMI1 and RING1B were diminished with the increment of the substrate stiffness (Figs. 1C and EV1H). Of note, the responsiveness of PcG condensates to the external mechanical forces was impaired in MSCs carrying *KMT2D* truncating mutations, and in KS patient-derived fibroblasts (Figs. 1C and EV1I–K), as well as in response by abrogating the formation of a functional LINC complex (Fig. EV1L). SIM microscopy indicated that the differences in PcG spatial organization were associated with a decrease in the abundance and the dimension of the condensates (Fig. 1D).

To rule out the contribution of PcG condensates to the altered responsiveness of the MLL4 ^Q4092X^ MSCs, we rescued its functionality by overexpressing the H3.3 K27M mutant, which has a dominant negative effect on PcG activity and compartmentalization (Fig. EV1M) commitment (Fasciani et al, 2020). We found that by restoring PcG clustering, we re-established the relative abundance of the transcriptional condensates, both on soft and stiff conditions (Fig. EV1N,O). These results suggest that the balancing between transcription and PcG condensates could be involved in cellular mechano-response by mediating the nuclear architecture and mechanical properties. Indeed, by quantifying the longitudinal elastic modulus calculated based on the Brillouin frequency shift (Prevedel et al, 2019), we found that the chromatin stiffness mirrored the abundance of PcG condensates (Fig. 1E,F). This pattern was reversed upon MLL4 LoF and was rescued by re-establishing PcG condensates by expressing the H3.3K27M mutant. These results were further corroborated by measuring nuclear stiffness by atomic force microscopy (AFM) (Appendix Fig. S3). To determine whether the measured MLL4-dependent increase in nuclear stiffening was associated with nucleoplasm viscosity, we performed micro-rheology measurements by adopting nuclear genetically encoded multimeric nanoparticles (nucGEMs) (Szoradi et al, 2022) (Fig. 1G,H). By monitoring the diffusion pattern of nucGEM through single molecule tracking (Fig. 1H,I), we retrieved information about the viscosity and macromolecular crowing of the nucleoplasm, which reflets changes in the nuclear biophysical properties (McCreery et al, 2025; Xia et al, 2025; Guo et al, 2017). We found that while the increased substrate stiffness caused a decrease in diffusion of nucGEM with a confined motion, which indicates an augmented nuclear viscosity, this response was attenuated in the MLL4 LoF condition (Fig. 1H–K). In sum, the depicted organization of chromatin condensates suggests that *KMT2D* haploinsufficiency impairs the nuclear mechano-response to ECM-mediated forces, strengthening the role of MLL4 in tuning the nuclear mechanical properties. These results indicate that chromatin condensates are responsive to external mechanical forces, and their balancing possibly tunes the nuclear mechanical properties.

### MLL4 abundance determines nuclear mechanical response to tissue stiffening

**Figure 2:**
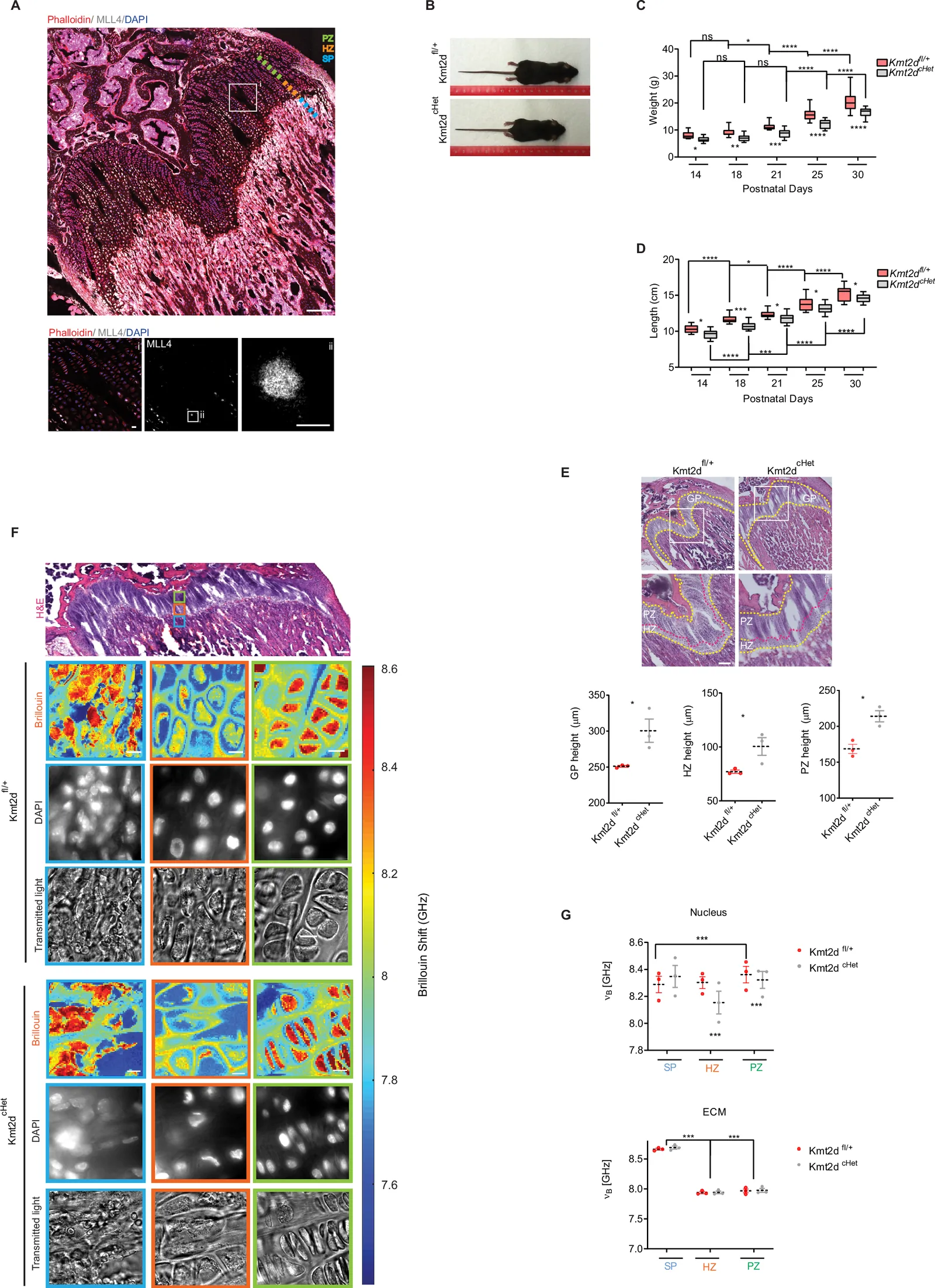
MLL4 abundance determines nuclear mechanical response to tissue stiffening. (**A**) Representative images of MLL4 (white)/Phalloidin (red) immunostaining, scale bars 100 µm. Lower panels: (i) enlargement of MLL4/Phalloidin immunostaining in hypertrophic and proliferative cells within the growth plate, (ii) single-cell crop showing MLL4 nuclear distribution. Scale bars 10 µm. (**B**) Representative images of Kmt2d^fl/+^ (WT) and Kmt2d^cHet^ (KS) at postnatal day 30. (**C**) Quantification of mice weight in Kmt2d^fl/+^ (WT) and Kmt2d^cHet^ (KS) at the indicated postnatal days (*n* = 18 mice derived from three different litters). The box plot indicates median values (middle lines), first and third quartiles (box edges) and 10th and 90th percentiles (error bars). Statistical significance was determined by Tukey’s and Sidak’s multiple comparison test (two-way ANOVA). (**D**) Quantification of the length (cm) of Kmt2d^fl/+^ (WT) and Kmt2d^cHet^ (KS) at the indicated postnatal days (*n* = 18 mice derived from three different litters). The box plot indicates median values (middle lines), first and third quartiles (box edges) and 10th and 90th percentiles (error bars). Statistical significance was determined by Tukey’s and Sidak’s multiple comparison test (two-way ANOVA). (**E**) Hematoxylin and Eosin (H&E) staining of Kmt2d^fl/+^ and Kmt2d^cHet^ femoral growth plate (GP), highlighted by a yellow dotted line. Within the GP, the hypertrophic and proliferative zones (HZ and PZ, respectively) are marked. Below, quantification of GP, HZ, and PZ height (data combined from three mice in each group). Statistical significance was determined by a two-tailed unpaired Student’s *t*-test. (**F**) Upper panel**:** representative image of H&E staining of the femur growth plate in Kmt2d^fl/+^ (WT). Within the GP are highlighted the SP in blue, the HZ in green and the PZ in orange. Scale bars, 100 µm. Below are representative maps of the stiffness distribution in the aforementioned regions, in Kmt2d^fl/+^ (WT) and Kmt2d^cHet^ (KS) GP. Scale bars, 10 µm. The DAPI and transmitted light signal are also included. (**G**) Quantification of nuclear (upper panel) and ECM (lower panel) stiffness (Brillouin shift) in primary spongiosa (SP), hypertrophic zone (HZ) and proliferative zone (PZ) in Kmt2d^fl/+^ (WT) and Kmt2d^cHet^ (KS). Bar plots show mean + SEM of data combined from three mice in each group. Statistical significance was determined by a linear mixed-effects model. *P* values definition: *p < 0.05, **p < 0.01, ***p < 0.001, ****p < 0.0001, ns not significant (*p* ≥ 0.05). Source data are available online for this figure.

Next, we asked whether the measured increment of MLL4 clustering in response to mechanical forces also occurs in vivo and what its role is in tissue mechano-response. We first analyzed the abundance and the nuclear distribution of MLL4 along mice’s growth plate located at the epiphysis of long bones, which is the tissue site in which bone elongation takes place by endochondral ossification (Mackie et al, 2011). Of importance, immunofluorescence analyses showed that MLL4 was non-homogeneously distributed within the nuclei of differentiating hypertrophic chondrocytes and osteoblasts while showing a low level of expression in the resting and proliferating chondrocytes that reside within the apical zone (Fig. 2A). Thereafter, we assessed the contribution of MLL4 to the nuclear mechanics of chondrocytes and osteoblasts, whose function and homeostasis are influenced by ECM-mediated mechanical forces (Saraswathibhatla et al, 2023). To this end, we generated a KS mouse model (thereafter named *Kmt2d*^*cHe*t^) by crossing homozygous *Kmt2d* conditional allele mice *(Kmt2d*^*fl/fl*^*)* with CMV-Cre transgene (Jang et al, 2019). By monitoring the body length and weight, we found that *Kmt2d*^*cHe*t^ mice showed a marked postnatal growth delay with respect to the *Kmt2d*^*fl/+*^ counterpart (Fig. 2B–D). Given this phenotype that resembles the postnatal growth delay affecting KS individuals (Boniel et al, 2021), we focused on measuring possible alterations in the morphology and tissue mechanics of the epiphysis of long bones. We noticed an enlargement of the growth plate and a poorly defined hypertrophic zone in the *Kmt2d*^*cHe*t^ condition compared to the *Kmt2d*^*fl/+*^ littermates (Fig. 2E). Brillouin microscopy showed that the ECM stiffness increased longitudinally along the growth plate, showing the highest rigidity at the level of the primary spongiosa, the site for cartilage mineralization of the newly elongating bones (Fig. 2F,G). In the same condition, we found that the chromatin stiffening anti-correlated with the ECM rigidity, showing a lower level of stiffness in the osteoblasts residing in the spongiosa, with respect to the chondrocytes. Of importance, osteoblasts of the *Kmt2d*^*cHe*t^ mice showed a relative increment of nuclear stiffness, while the ECM mechanical properties were unperturbed. On the contrary, within the hypertrophic zone of the growth plate of the *Kmt2d*^*cHe*t^ mice, which is characterized by a modest ECM stiffness, we measured an augmented nuclear stiffening (Fig. 2F,G). Remarkably, the same change in nuclear mechanical properties was not detected within the proliferative zone, which is characterized by a low level of expression of MLL4 as well as by an increment of ECM stiffening in the *Kmt2d*^*cHe*t^ mice (Fig. 2F,G). In sum, the depicted organization of chromatin condensates suggests that *KMT2D* haploinsufficiency impairs the nuclear mechano-response to ECM-mediated forces, strengthening the role of MLL4 in tuning the nuclear mechanical properties.

### MLL4 condensates are responsive to external mechanical forces

**Figure 3:**
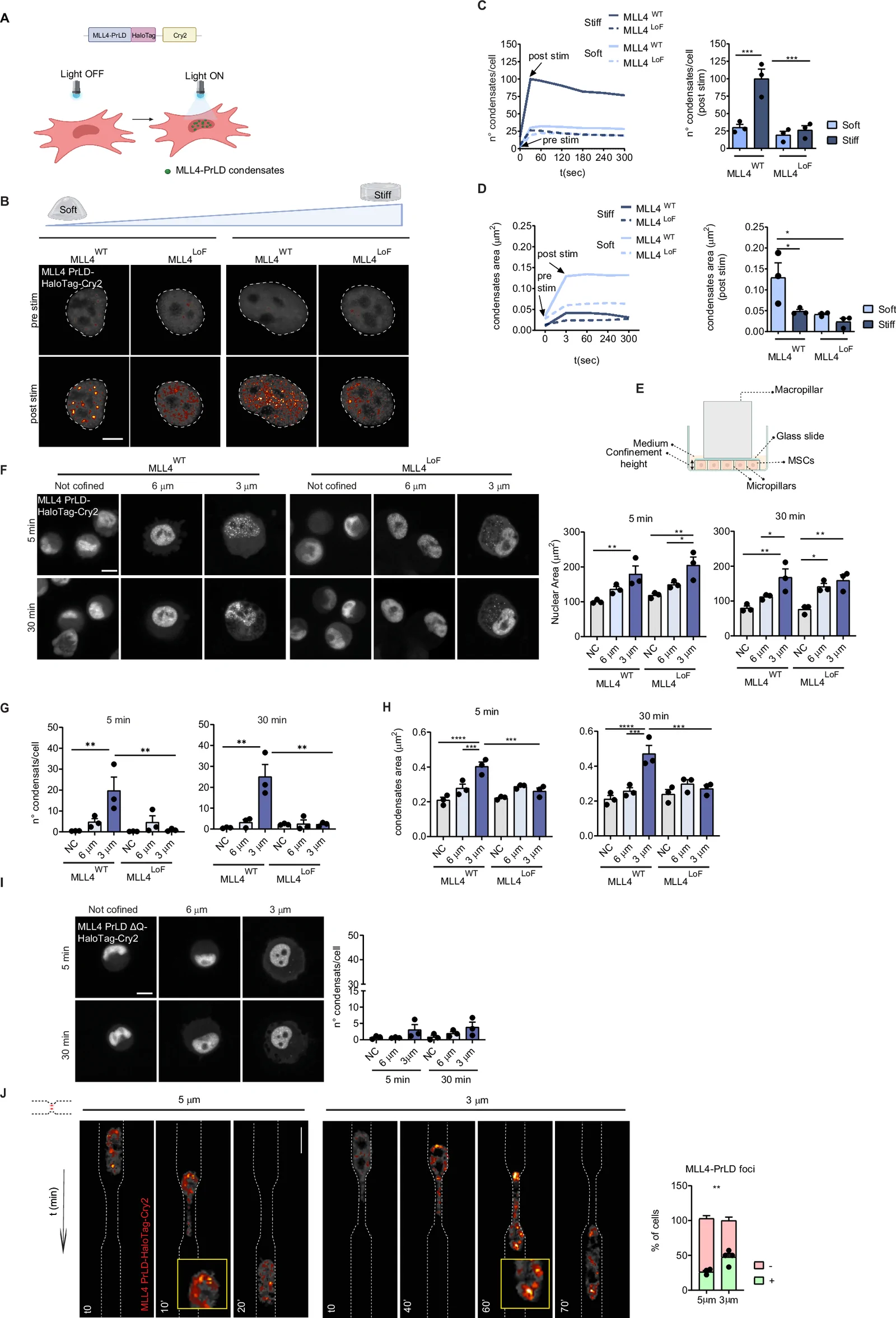
MLL4 condensates are responsive to external mechanical forces. (**A**) Schematic representation of the MLL4-PrLD-HaloTag-Cry2 optogenetic tool. Illustration created with Biorender.com. (**B**) Representative images of blue light-induced clustering of MLL4-PrLD-HaloTag-Cry2 before and after the stimulus in MLL4^WT^ and MLL4^LoF^ MSCs on soft and stiff matrix. Dashed borders highlight the cell nucleus. The cells were stimulated for 3 s with 488-nm light, followed by image acquisition. Scale bars, 5 µm. (**C**,** D**) Quantification of the number (**C**) and area (**D**) of light-induced condensates of MLL4-PrLD-HaloTag-Cry2 at different time points (Mean of *n* = 3 independent biological replicates) and in the post-stimulus (bar plots show mean + SEM of *n* = 3 independent biological replicates; Sidak’s multiple comparison test—two-way ANOVA). The time point *t* = 0 represents the pre-stimulus. (**E**) Schematic representation of the cell confinement. Illustration created with BioRender.com. The height of confinement is controlled by polydimethylsiloxane (PDMS) micropillars, fabricated in a glass slide and attached to a PDMS piston. Illustration created with BioRender.com. (**F**) Representative images of MLL4^WT^ and MLL4^LoF^ MSCs expressing MLL4-PrLD-HaloTag-Cry2 unconfined or confined at 3 and 6 μm. Cells were confined in suspension. Scale bars, 5 µm. On the right, quantification of nuclear area are plotted as mean + SEM of *n* = 3 independent biological replicates; Sidak’s multiple comparison test (two-way ANOVA). (**G**) Quantification of the number and area (**H**) of MLL4-PrLD-HaloTag-Cry2 condensates 5 and 30 min post confinement detected in MLL4^WT^ and MLL4^LoF^ MSCs not confined, and confined at 3 and 6 μm. Bar plots show mean + SEM of *n* = 3 independent biological replicates; Sidak’s multiple comparison test (two-way ANOVA) (**I**) Representative images and relative condensate quantification of MLL4-PrLD-ΔQ-HaloTag-Cry2 in MLL4^WT^ MSCs not confined, and confined at 3 and 6 μm. Bar plots show mean + SEM of *n* = 3 independent biological replicates; Sidak’s multiple comparison test (two-way ANOVA). Scale bars, 5 µm. (**J**) Representative images of MLL4^WT^ MSCs expressing MLL4-PrLD-HaloTag-Cry2 passing through constrictions of 5 and 3 μm at different time points. Dashed white lines highlight the microchannel borders. On the right, quantification of the percentage of cells that spontaneously form (+) or do not form (−) MLL4-PrLD condensates. Bar plots show mean + SEM of *n* = 4 independent biological replicates. Statistical significance was determined by a two-tailed unpaired Student’s *t*-test. Scale bars, 5 µm. *P* values definition: *p < 0.05, **p < 0.01, ***p < 0.001, ****p < 0.0001, ns not significant (*p* ≥ 0.05). Source data are available online for this figure.

**Figure EV2:**
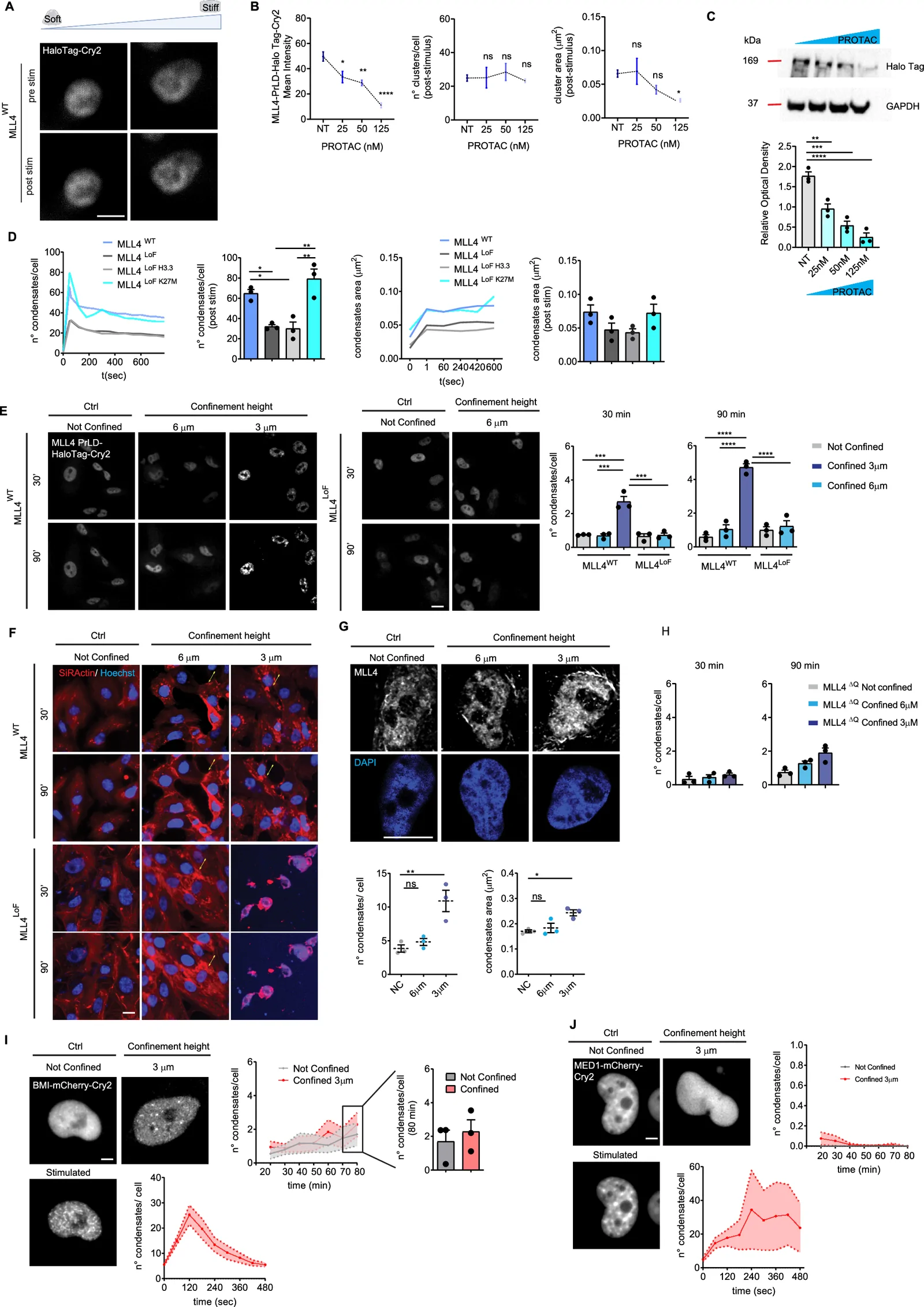
MLL4 condensates respond to external mechanical forces. (**A**) Representative images of MSCs on soft and stiff matrix overexpressing HaloTag-Cry2 before and after the stimulus with blue light. Scale bars, 10 µm. (**B**) Quantification of the number and area of light-induced droplets of MLL4-PrLD-HaloTag-Cry2 in MLL4^WT^ MSCs after the blue light stimulus treated with PROTAC3 at the concentration indicated (NT nontreated). Bar plots show mean + SEM of *n* = 3 independent biological replicates. Statistical significance was determined by Dunnett’s multiple comparison test (Welch and Brown–Forsythe one-way ANOVA). (**C**) Western Blot analysis of MLL4-PrLD-HaloTag-Cry2 in MLL4^WT^ MSCs after PROTAC3 treatment (*n* = 3 independent experiments). Bar plots show mean + SEM of *n* = 3 independent biological replicates. Statistical significance was determined by Dunnett’s multiple comparison test (one-way ANOVA). (**D**) Quantification of the number and area of light-induced droplets of MLL4-PrLD-HaloTag-Cry2 at different time points in MLL4^WT^ and MLL4^LoF^ MSCs, as well as MLL4^LoF^ MSCs expressing either H3.3WT or H3.3K27M on soft and stiff matrix. The time point *t* = 0 represents the pre-stimulus. Bar plots show quantifications of n° condensates/cell in the post-stimulus (mean + SEM of *n* = 3 independent biological replicates). Statistical significance was determined by Sidak’s multiple comparison test (two-way ANOVA). (**E**) Representative image of MLL4^WT^ and MLL4^LoF^ MSCs overexpressing MLL4-PrLD-HaloTag-Cry2 not confined or confined at 6 and 3 μm in adhesion for 30 and 90 min. Scale bars, 15 µm. On the right, quantification of the number of condensates/cell at the indicated time points. (mean + SEM of *n* = 3 independent biological replicates). Statistical significance was determined by Sidak’s multiple comparison test (two-way ANOVA). (**F**) Representative images showing adherent MLL4^WT^ and MLL4^LoF^ MSCs unconfined, or confinement at 6 and 3 μm, at the indicated time points. Cell nuclei are stained with Hoechst (blue), whether the cytoskeleton is marked in red (SirActin). Yellow arrows indicate actomyosin recruitment to the cell-cortex in response to confinement. Scale bars, 15 µm. (**G**) Representative confocal images and relative quantifications of immunostaining for MLL4 in MLL4^WT^ MSCs unconfined and confined at 6 and 3 μm for 45 min. Scale bars, 15 µm. Bar plots show mean + SEM of *n* = 3 independent biological replicates. Statistical significance was determined by Dunnett’s multiple comparison test (one-way ANOVA). (**H**) Quantification of the number of condensates/cell in MLL4^WT^ MSCs expressing MLL4-PrLD-ΔQ-HaloTag-Cry2 confined in adhesion at 6 and 3 μm for 30 and 90 min. Bar plots show mean + SEM of *n* = 3 independent biological replicates. Statistical significance was determined by Sidak’s multiple comparison test (two-way ANOVA). (**I**,** J**) Representative images of MLL4^WT^ MSCs expressing BMI-mCherry-Cry2 (**I**) and MED1-mCherry-Cry2 (**J**) in not confined cells +/− blue light stimulation and confined at 3 µm. On the right, quantification of the number of condensates at different time points is shown upon confinement and upon stimulation. Bar plots show mean + SEM of *n* = 3 independent biological replicates; two-tailed unpaired Student’s *t*-test. Scale bars, 5 µm. *P* values definition: *p < 0.05, **p < 0.01, ***p < 0.001, ****p < 0.0001, ns not significant (*p* ≥ 0.05).

To investigate the mechanism by which transcriptional condensates respond to external mechanical forces, we monitored the assembly of MLL4 condensates in living cells using the light-activated optoIDR system (Shin et al, 2017; Fasciani et al, 2020) (Fig. 3A). A single pulse of blue light triggered the clustering of MLL4_PrLD (MLL4 Prion-like domain) into spherical droplets whose dimensions scaled proportionally to the protein abundance, as expected (Fig. EV2A–C). Time-lapse imaging showed that optoMLL4 initially nucleated into discrete droplets, which progressively coalesced into larger condensates over time (Fig. 3B–D). Notably, we found that the MLL4 clustering into condensates was sensitive to external mechanical cues. Indeed, MSCs grown on soft matrices exhibited reduced formation of MLL4-PrLD condensates, along with an increase in condensate size compared to cells on stiff matrices (Fig. 3B–D). We then assessed the contribution of the chromatin context to the dynamic clustering of optoMLL4 in these mechanical regimes. We found that the assembly of the optoMLL4 in response to external mechanical forces was impaired in MLL4^Q4092X^ MSCs, giving rise to a reduced number of smaller condensates (Fig. 3B–D). Notably, restoring PcG condensate levels through expression of the H3.3 K27M mutant rescued the mechano-responsiveness of optoMLL4 clustering (Fig. EV2D). These results indicate that matrix stiffness influences MLL4-PrLD condensate formation, and that MLL4 loss-of-function compromises its dynamic assembly in response to mechanical cues.

To determine whether the PrLD of MLL4 functions as a chromatin mechano-sensor, we assessed its dynamic assembly in response to applied mechanical load through nuclear confinement, independently of CRY2-mediated nucleation (Fig. 3E). We found that increased confinement triggered the assembly of MLL4-PrLD into spherical condensates, whose number and size increased proportionally over time (Fig. EV2E,F and Movie EV1). Consistently, a comparable mechano-response was detected by monitoring the clustering of the endogenous MLL4 in MSCs (Fig. EV2G). Given the central role of the polyQ tract in MLL4 clustering (Fasciani et al, 2020), we next evaluated its contribution to the mechano-response. Deletion of the polyQ stretch weakened MLL4-PrLD condensate formation upon cell confinement (Fig. EV2H). To assess whether this mechanical responsiveness is specific to MLL4, we applied the optoIDR system to other chromatin-associated factors known to form condensates, including MED1 and BMI1. Although both proteins assembled into condensates following blue light stimulation (Fig. EV2I), neither responded to mechanical confinement (Fig. EV2J), indicating that mechano-responsiveness is not a general property of chromatin condensates.

We next investigated the contribution of the MLL4-dependent chromatin context to the dynamic clustering of optoMLL4 in response to cell confinement. While both wild-type and MLL4^Q4092X^ MSCs displayed comparable cytoskeletal organization, MLL4 LoF showed a marked increase in cell death under more restrictive conditions (Fig. EV2F), possibly associated with the combinatorial effects of ECM-associated forces and mechano-osmotic signal (Lomakin et al, 2020). To overcome this limitation and improve temporal resolution, we monitored PrLD assembly in MSCs maintained in suspension and subjected to confinement, thereby decoupling the response from ECM-mediated mechanical forces (Jang et al, 2019). We measured a time- and height-dependent increase of PrLD clustering, which was depicted 5 min after applying cell confinement (Fig. 3F–H). Notably, this response was impaired in the MLL4^Q4092X^ MSCs and upon deletion of the polyQ stretch of the PrLD of MLL4 (Fig. 3F–I). Finally, we assessed the clustering of MLL4-PrLD upon migration of MSCs throughout microchannels, which imposes a transient mechanical load on the nucleus. Accordingly, we observed a progressive increase in MLL4-PrLD clustering proportional to the degree of mechanical constraint (Fig. 3J; Movies EV2 and 3). Collectively, these results indicate that MLL4 dynamically assembles into chromatin condensates in response to nuclear mechanical load, acting as a chromatin mechano-sensor through its PrLD.

### Mechanical forces guide the folding transition of the polyQ stretch of MLL4-PrLD

**Figure 4:**
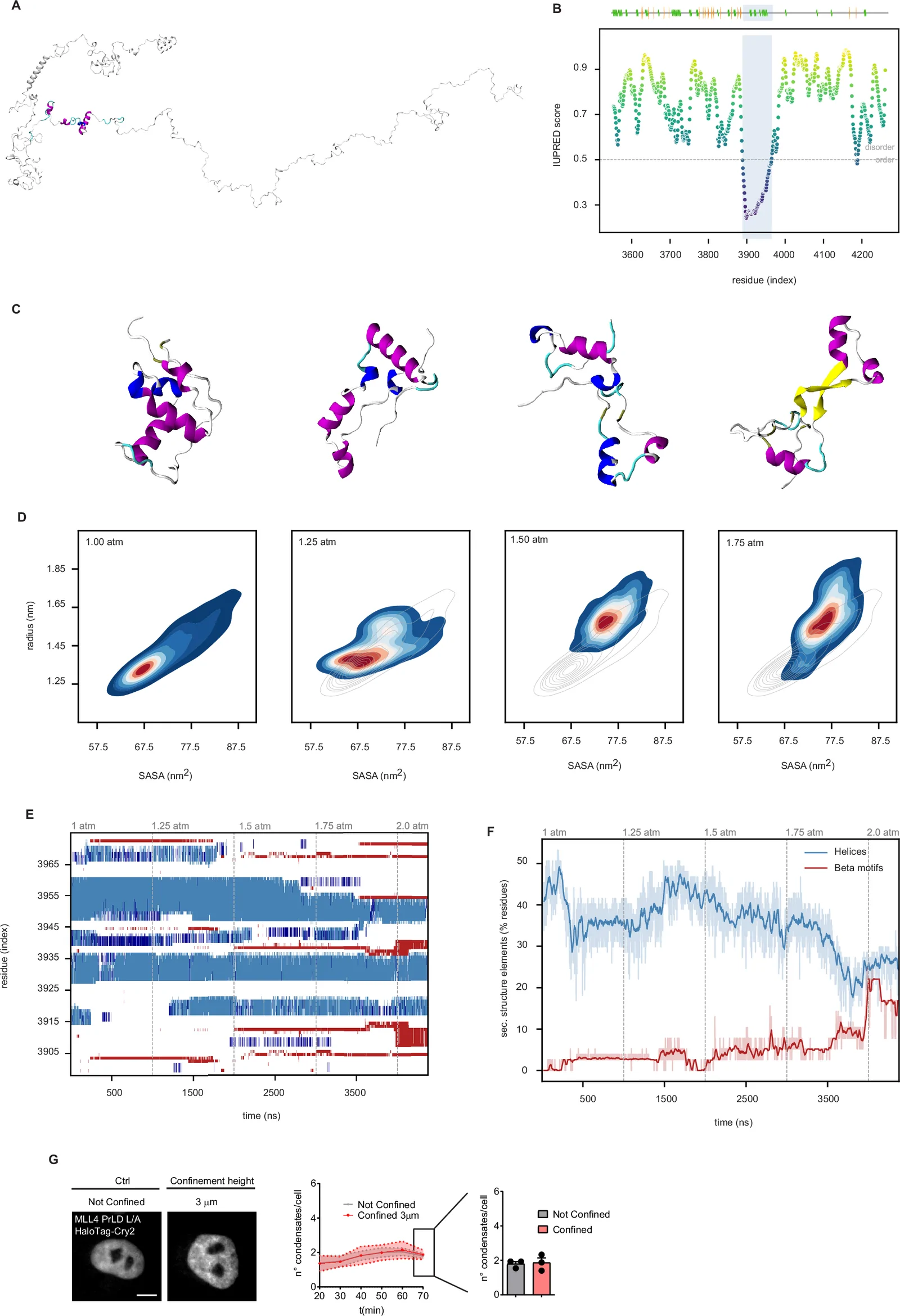
Mechanical forces guide the folding transition of the polyQ stretch of MLL4-PrLD. (**A**) Structure prediction of the Prion-like domain of MLL4 (MLL4-PrLD, residues 3561 to 4270 of the MLL4 sequence); the polyQ tract at the core of MLL4-PrLD (residues 3898 to 3974) is highlighted. (**B**) Top: Layout of the secondary structure motifs associated with the structural prediction of MLL4-PrLD by the IntFold web server: Helices and beta strands are shown as coils and arrows, respectively, and the polyQ tract of MLL4-PrLD (residues 3898 to 3974) is highlighted (shaded area). Illustration obtained via the Biotite Python toolkit. Bottom: Prediction of structural disorder of the MLL4-PrLD based on the IUPRED bioinformatics tool: Lower IUPRED scores are associated with a higher tendency for each residue to fold into ordered structures. Likewise, the polyQ tract of MLL4-PrLD is highlighted (shaded area). (**C**) Snapshots of the structure of the polyQ tract of MLL4-PrLD from MD simulations, taken as the final frames of the 1-μs trajectories at 1 (leftmost), 1.25, 1.5, and 1.75 atm (rightmost), highlighting a consistent evolution in the secondary and tertiary structure, and the emergence of beta-like motifs—shown in yellow. (**D**) Putative free energy profiles of the polyQ tract of the Prion-like domain of MLL4 (MLL4-PrLD), along the twofold reaction coordinate defined by the radius of gyration and the solvent-accessible surface area (SASA). All subplots relate to a different target pressure of the molecular dynamics (MD) setup, whereby each value of *P* has been sampled for 1  μs. A color scheme defines the likelihood for the system to adopt specific values of the reaction coordinates throughout the MD trajectory, from blue (lowest) to red (highest frequency): A transition between two basins—towards an expanded conformation of the polyQ tract—is associated with the external pressure ramp. In fact, a decreasing trend of both reaction coordinates observed in the earliest frames at 1 atm (which is replicated within each subplot by dark gray contour lines) relates to an early equilibration stage of the system. (**E**) Left: Profile of the evolution of the secondary structure of the polyQ tract of MLL4-PrLD via MD simulations: Each residue is attributed a secondary structure motif as beta strands (red), alpha helices (cyan), alt. helices (blue), coils/turns (white) on a frame-wise basis along the trajectory - dashed vertical lines mark the stages of the pressure “ramp”. (**F**) Fraction of residues of the polyQ tract of MLL4-PrLD involved with either helices or beta motifs along the MD trajectory, on a frame-wise basis - likewise, dashed vertical lines mark the stages of the pressure ramp, whereas rolling averages of the signals are included as darker lines for clarity. (**G**) Representative images of MLL4^WT^ MSCs overexpressing MLL4-PrLD L/A-HaloTag-Cry2 in not confined cells +/− blue light stimulation and confined at 3 µm. On the right, quantification of the number of MLL4-PrLD L/A-HaloTag-Cry2 condensates at different time points upon stimulation and upon confinement (Mean of *n* = 3 independent biological replicates). Bar plots of n° condensates per cell upon confinement show mean + SEM of *n* = 3 independent biological replicates; two-tailed unpaired Student’s *t*-test. Scale bars, 5 µm. *P* values definition: *p < 0.05, **p < 0.01, ***p < 0.001, ****p < 0.0001, ns not significant (*p* ≥ 0.05). Source data are available online for this figure.

**Figure EV3:**
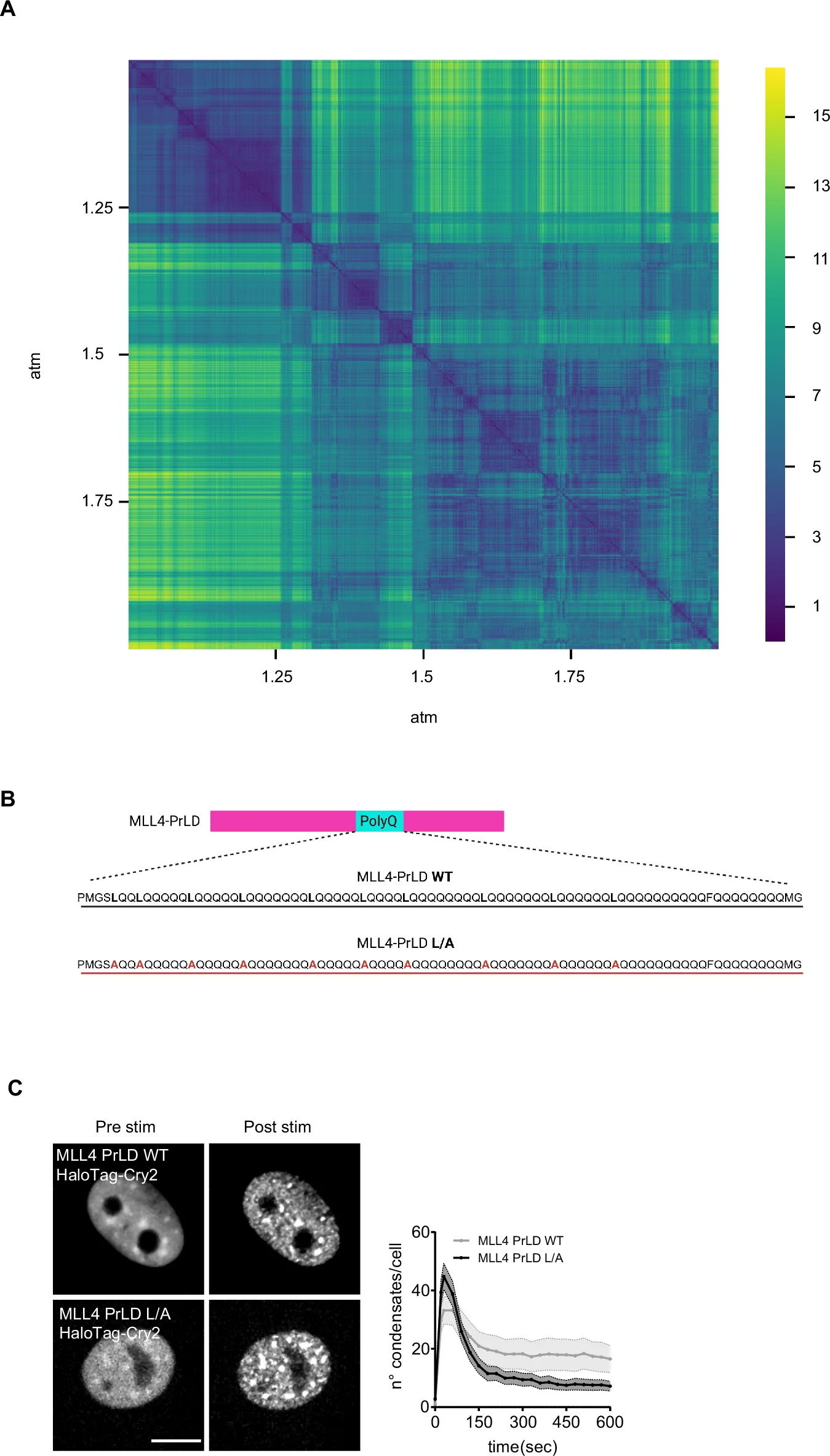
Mechanical forces determine the folding transition of the polyQ stretch of MLL4-PrLD. (**A**) Two-dimensional root mean square displacement (RMSD) profile of the polyQ tract of MLL4-PrLD, based upon the coordinates of the two most stable helices along the MD trajectory (i.e., conserved in over 75% of the trajectory frames and involving residues 3928–3935 and residues 3948–3954). A low value of the RMSD is associated with a high degree of structural/conformational similarity between frames: Here, two broad squares (blue shades) might be identified that corroborate the existence of a twofold conformational basin of the polyQ tract, the higher pressure regimes stabilizing a more expanded configuration. Analysis performed within the MD Analysis framework (PMID: 15973002, 21500218). (**B**) Schematic representation of MLL4 PrLD L/A substitutions within the PolyQ stretch. (**C**) Representative images of MSCs overexpressing MLL4-PrLD WT-/ MLL4-PrLD L/A-HaloTag-Cry2 before and after the stimulus with blue light. On the right, quantifications of n° condensates/cell (merge of two biological replicates). Scale bars, 10 µm. *P* values definition: *p < 0.05, **p < 0.01, ***p < 0.001, ****p < 0.0001, ns not significant (*p* ≥ 0.05).

As mechano-signaling relies on the capability of macromolecules to sense and transmit the received signals by transiently changing their conformations (Beedle and Garcia-Manyes, 2023; Saini and Discher, 2019), we investigated whether the PrLD of MLL4 undergoes a folding transition in response to mechanical load. Structural predictions of the MLL4-PrLD highlighted that, despite belonging to an intrinsically disordered region, its embedded polyQ tract displays an intrinsic propensity to adopt locally folded conformations, such as α-helixes (Fig. 4A,B). This observation supported the choice of this region as the object of a molecular dynamics (MD) simulation campaign. To explore the mechanistic role of the polyQ tract in MLL4 clustering, we performed MD simulations by applying an increasing external pressure (1.0 to 1.75 atm) to a single polyQ segment, thereby mimicking the effects of mechanical forces exerted on the system to investigate their consequences on the protein’s conformational landscapes. Under these conditions, the dynamics of the isolated polyQ tract revealed a broad transition between conformational basins, shifting from a “compact” to a more “expanded” configuration (Figs. 4D and EV3A). This transition is mainly accounted for by a re-adjustment of the stable helical motifs in the sequence, that are maintained in over 75% of the trajectory frames, involving residues 3929–3936 and 3949–3955 (Fig. 4C).

Notably, this global conformational transition is accompanied by a steady reduction in α-helical content and the emergence of β-like motifs (Fig. 4F)—the latter being sparsely distributed in the initial stages of the MD trajectory, eventually yielding a stably-folded, extended beta sheet in an antiparallel arrangement (Fig. 4C–F). Increasing the applied pressure enhances this transition and involves residues originally belonging to both coiled and helical regions (Fig. 4E,F). Moreover, the newly-achieved ***β***-motif is conserved by further adjusting the target external pressure, as observed in extended simulations at 2 atm. Thus, molecular dynamics simulations suggest that the polyQ tract of MLL4-PrLD exhibits several conformational polymorphs, with increasing external pressure favoring the stabilization of an extended, β-rich configuration.

To experimentally assess the contribution of these conformational transitions to MLL4 mechano-responsiveness, we examined PrLD assembly under mechanical load. Considering the propensity of the interspersed Leucine within the PolyQ stretch to contribute to ***β***-rich configurations, we substituted them with Alanine and measured the responsiveness of the mutated optoMLL4 to mechanical load. We found that the L-to-A substitutions abrogated MLL4 coacervation upon cell confinement, while its responsiveness to blue-light stimulation was only marginally affected (Figs. 4G and EV3B,C).

### Unbalancing chromatin condensates affects nuclear shape in response to mechanical cues

**Figure 5:**
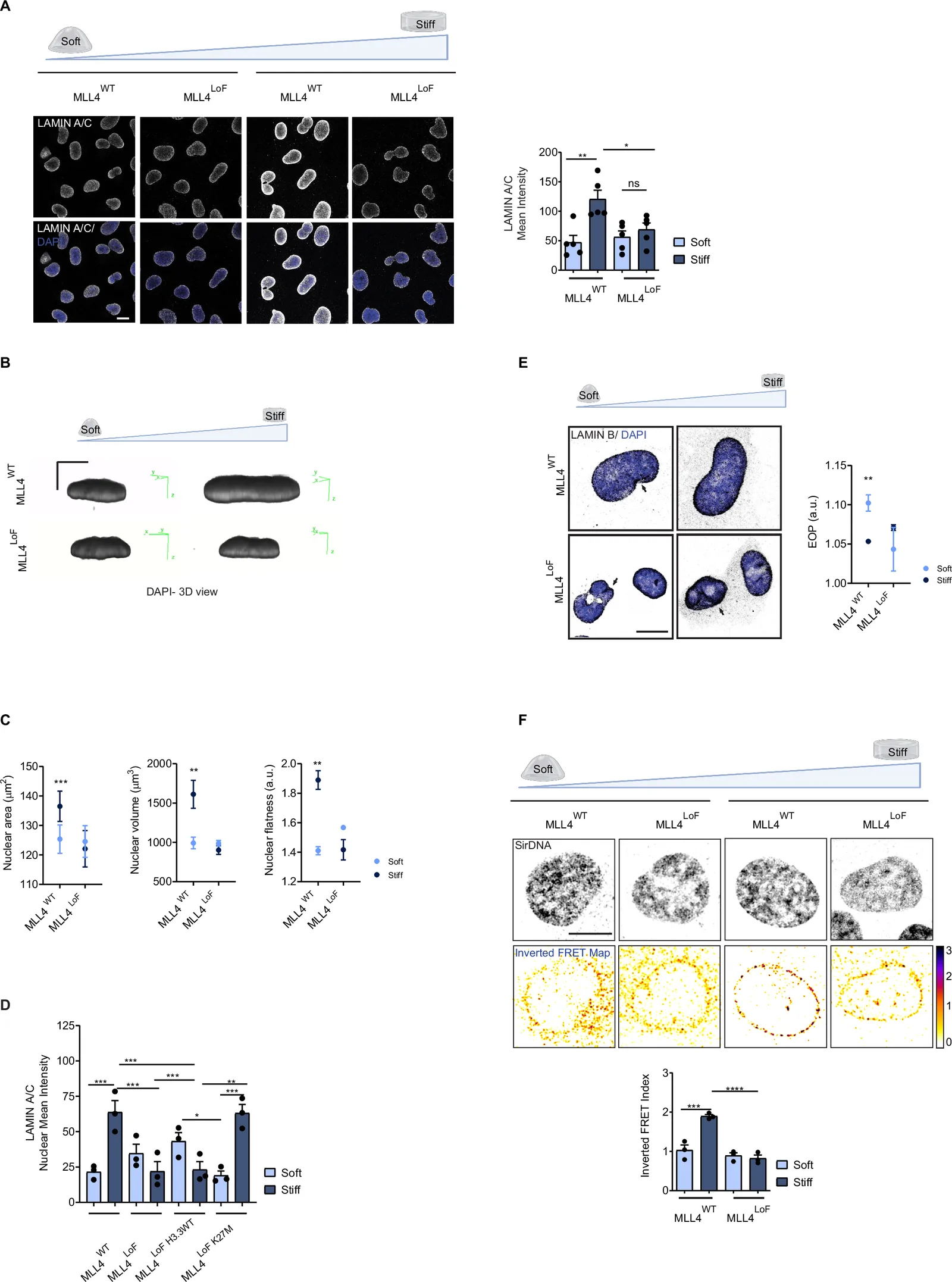
Unbalancing chromatin condensates affects nuclear envelope shape in response to mechanical cues. (**A**) Representative confocal images and relative quantifications of immunostaining for LAMIN A/C in MLL4^WT^ and MLL4^LoF^ MSCs on soft and stiff matrix. Bar plots show mean + SEM of *n* = 5 independent biological replicates. Statistical significance was determined by Sidak’s multiple comparison test (two-way ANOVA). Scale bars, 10 µm. (**B**) 3D side view of MLL4^WT^ and MLL4^LoF^ MSCs nuclei stained with DAPI on soft and stiff matrix. Scale bars, 10 µm. (**C**) Quantification of nuclear volume, area, and flatness in MLL4^WT^ and MLL4^LoF^ MSCs on soft and stiff matrix. Bar plots show mean + SEM of *n* ≥ 3 independent biological replicates. Statistical significance was determined by a linear mixed-effects model. (**D**) Quantifications of immunostaining for LAMIN A/C in MLL4^WT^ and MLL4^LoF^ MSCs, as well as MLL4^LoF^ MSCs expressing either H3.3WT or H3.3K27M on soft and stiff matrix. Bar plots show mean + SEM of *n* = 3 independent biological replicates. Statistical significance was determined by a linear mixed-effects model. (**E**) Representative confocal images of immunostaining for LAMIN B in MLL4^WT^ and MLL4^LoF^ MSCs on soft and stiff matrix. Black arrows indicate the presence of nuclear envelope invaginations, quantified as EOP (Excess Of Perimeter). Bar plots show mean + SEM of *n* = 4 independent biological replicates. Statistical significance was determined by a linear mixed-effects model. (**F**) Representative images showing the FRET/Donor maps of the Mini-Nesprin FRET sensor in MLL4^WT^ and MLL4^LoF^ MSCs on soft and stiff matrices. Below, a quantification of the Inverted FRET; lower values indicate decreased tension on the nuclear envelope. Bar plots show mean + SEM of *n* = 3 independent biological replicates. Statistical significance was determined by Sidak’s multiple comparison test (two-way ANOVA). Scale bars, 10 µm. *P* values definition: *p < 0.05, **p < 0.01, ***p < 0.001, ****p < 0.0001, ns not significant (*p* ≥ 0.05). Source data are available online for this figure.

**Figure EV4:**
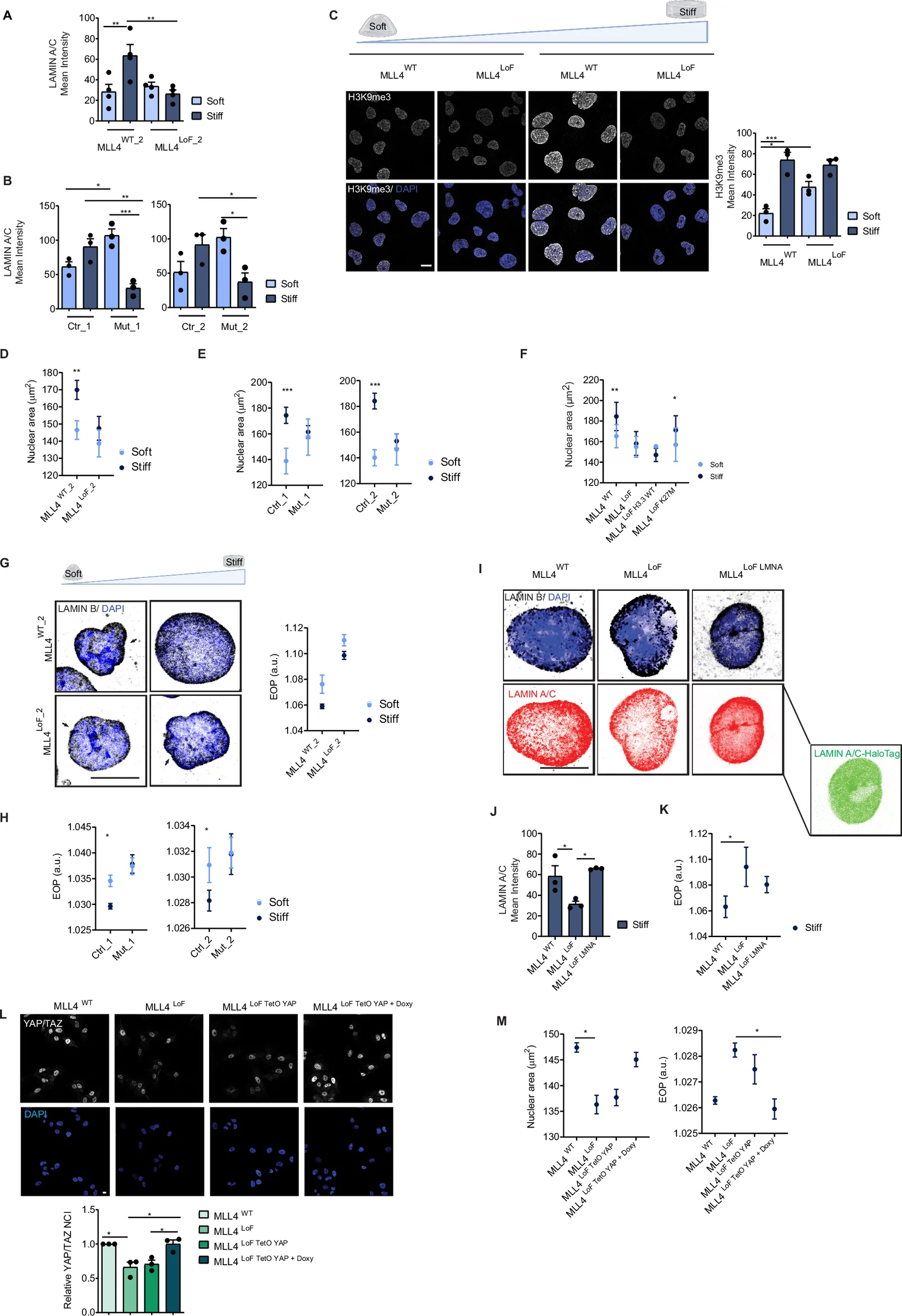
Unbalancing chromatin condensates affects nuclear envelope tension in response to substrate stiffness. (**A**) Quantifications of immunostaining for LAMIN A/C in MLL4^WT_2^ and MLL4^LoF_2^ MSCs, independent clones on soft and stiff matrix. Bar plots show mean + SEM of *n* = 4 independent biological replicates. Statistical significance was determined by Sidak’s multiple comparison test (two-way ANOVA). (**B**) Quantifications of nuclear LAMIN A/C mean intensity in primary fibroblasts from healthy donors (Ctrl) or Kabuki patients (Mut) on soft and stiff matrix. Bar plots show mean + SEM of *n* = 3 independent biological replicates. Statistical significance was determined by Sidak’s multiple comparison test (two-way ANOVA). (**C**) Representative confocal images and relative quantifications of immunostaining for H3K9me3 in MLL4^WT^ and MLL4^LoF^ MSCs on soft and stiff matrix. Scale bars, 10 µm. Bar plots show mean + SEM of *n* = 3 independent biological replicates. Statistical significance was determined by Sidak’s multiple comparison test (two-way ANOVA). Quantifications of nuclear area in MLL4^WT^ and MLL4^LoF^ MSCs independent clones (**D**) and in primary fibroblasts (**E**) on soft and stiff matrix. Bar plots show mean + SEM of *n* ≥ 3 independent biological replicates. Statistical significance was determined by a linear mixed-effects model. (**F**) Quantifications of the nuclear area in MLL4WT and MLL4LoF MSCs and MLL4LoF MSCs expressing either H3.3WT or H3.3K27M on soft and stiff matrix. Bar plots show mean + SEM of *n* ≥ 3 independent biological replicates. Statistical significance was determined by a linear mixed-effects model. Representative confocal images and relative quantification of immunostaining for LAMIN B in MLL4^WT^ and MLL4^LoF^ MSCs independent clones (**G**) and primary fibroblasts (**H**) on soft and stiff matrix. Black arrows indicate the presence of nuclear envelope invaginations, quantified as EOP (excess of perimeter). Scale bars, 10 µm. Bar plots show mean + SEM of *n* = 3 independent biological replicates. Statistical significance was determined by a linear mixed-effects model. (**I**) Representative confocal images of immunostaining for LAMIN B and LAMIN A/C in MLL4^WT^ and MLL4^LoF^ MSCs, as well as MLL4^LoF^ MSCs expressing Halo-tag LAMIN A/C. Bar plots (mean + SEM) showing quantification of LAMIN A/C (**J**) and EOP (**K**) in MLL4^WT^, MLL4^LoF^ and MLL4^LoF^ expressing Halo-tag LAMIN A/C MSCs. Scale bars, 10 µm. *N* ≥ 3 independent biological replicates. Statistical significance was determined by Sidak’s multiple comparison test (two-way ANOVA) for pane (**J**) and linear mixed-effects model for panel (**K**). (**L**) Representative confocal images of immunostaining for YAP/TAZ in MLL4^WT^, MLL4^LoF^, and MLL4^LoF^ MSCs overexpressing TetO YAP (±24 h doxycycline treatment) on stiff matrix. Scale bars, 10 µm. Below, quantification of the relative nuclear to cytoplasmic ratio (NCI). Bar plots show mean + SEM of *n* = 3 independent biological replicates. Statistical significance was determined by Tukey’s multiple comparison test (two-way ANOVA). (**M**) Quantification of nuclear area and nuclear envelope invaginations (EOP, based on LAMIN B immunostaining) in MLL4^WT^, MLL4^LoF^, and MLL4^LoF^ MSCs overexpressing TetO YAP (±24 h doxycycline treatment) on stiff matrix. Mean of ≥3 independent biological replicates is plotted. *P* values definition: *p < 0.05, **p < 0.01, ***p < 0.001, ****p < 0.0001, ns not significant (*p* ≥ 0.05).

In light of these findings, we measured whether MLL4 LoF impaired the responsiveness of MSCs to the increment of mechanical forces. We first investigated the contribution of Lamin A/C to define the nuclear mechanics, considering that its abundance increases in response to ECM stiffness (Swift et al, 2013) (Kalukula et al, 2022) We found that while in the wild-type cells Lamin A/C abundance increased in response to the augmented stiffening of the ECM, the *KMT2D* haploinsufficiency resulted in reduced responsiveness to the same mechanical load (Fig. 5A; Appendix Figs. S1 and S4). Remarkably, the impaired responsiveness to ECM stiffness was further verified using an independent clone of MSCs, and in patient-derived fibroblasts (Fig. EV4A,B). Of note, a similar pattern was observed when we analyzed the level and distribution of H3K9me3, a marker of constitutive heterochromatin, which is frequently associated with the nuclear lamina (van Steensel and Belmont, 2017) (Fig. EV45C). By analyzing the nuclear morphology, we found that the nuclear area, volume, and flatness were increased in cells maintained on a stiff matrix, and this response was weakened upon MLL4 LoF (Figs. 5B,C and EV4D,E). Notably, in the same cellular condition, we re-established the nuclear shape and the relative abundance of Lamin A/C by rescuing the clustering of PcG condensates (Figs. 5D and EV4F). By measuring the excess of the nuclear perimeter, we found that the adjustment of the nuclear shape in response to the increment of mechanical forces was mirrored by an augmented NE perimeter (Figs. 5E and EV4G,H). The NE of *KMT2D* haploinsufficient cells showed invaginations and a reduced nuclear perimeter, resembling the pattern observed in cells maintained on a soft matrix (Figs. 5E and EV4G,H). To rule out the contribution of the Lamin A/C decrease to the altered nuclear shape, we restored its abundance by expressing Lamin A-HaloTag in MLL4^Q4092X^ MSCs. In this setting, we did not observe any rescue of the NE surface despite a comparable level of Lamin A/C (Fig. EV4I,J), indicating that the altered nuclear shape was not solely dependent on the relative abundance of Lamin A/C. Considering that MLL4 LoF was associated with reduced nuclear YAP/TAZ (Fasciani et al, 2020) that controls nuclear shape and NE integrity (Sladitschek-Martens et al, 2022), we investigated its potential function in ensuring nuclear structure, in the context of KS. We found that by re-establishing YAP/TAZ abundance in MLL4^LoF^ MSCs, we rescued the altered nuclear shape and NE invaginations (Fig. EV4L,M). Finally, to measure the cytoskeletal forces acting on the NE in response to ECM stiffness, we adopted the Mini-Nesprin-1 reporter as the FRET sensor(Poli et al, 2023). We found that while in the MLL4^WT^ MSCs, the pulling forces increased with the stiffening of the matrix, this responsiveness was impaired upon MLL4 LoF (Fig. 5F). In sum, the obtained results indicate that the *KMT2D* haploinsufficiency reduces the NE responsiveness to the external mechanical load.

### MLL4 LoF impairs nuclear envelope integrity upon mechanical stress

**Figure 6:**
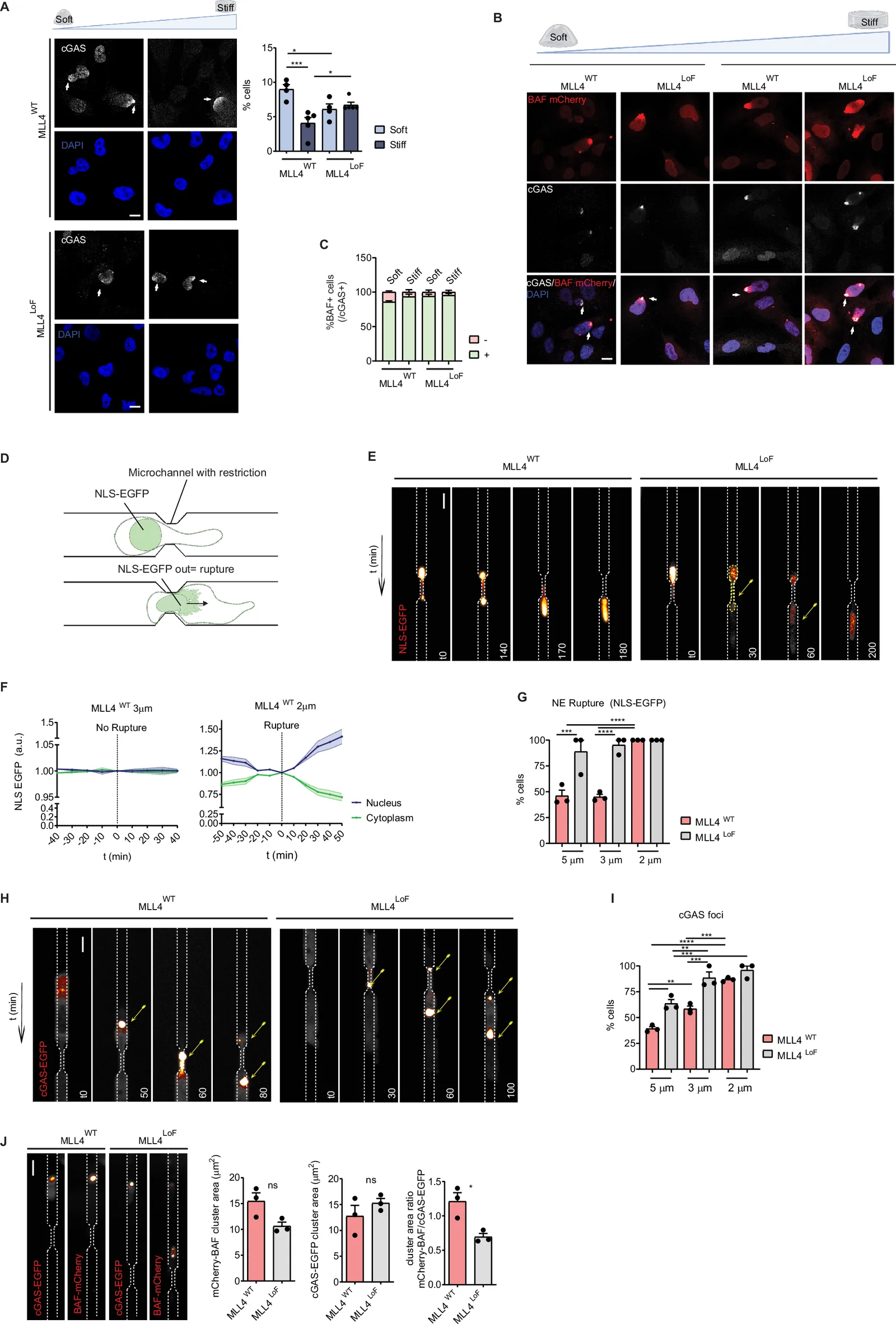
MLL4 LoF impairs nuclear envelope integrity upon mechanical stress. (**A**) Representative confocal images of immunostaining for cGAS in MLL4^WT^ and MLL4^LoF^ MSCs on soft and stiff matrix. Scale bars, 10 µm. On the right, quantification of the percentage of cells showing cGAS accumulation at the nuclear periphery, an indication of NE rupture (white arrows). Bar plots show mean + SEM of *n* ≥ 4 independent biological replicates. Statistical significance was determined by Sidak’s multiple comparison test (two-way ANOVA). (**B**) Representative confocal images of immunostaining for cGAS (white) in MLL4^WT^ and MLL4^LoF^ MSCs overexpressing BAF-mCherry (red) on soft and stiff matrix. Scale bars, 10 µm. (**C**) Quantification of the percentage of cGAS-positive cells showing also BAF accumulation at the nuclear periphery in correspondence of the cGAS condensates (colocalization). Bar plots show mean + SEM of *n* = 3 independent biological replicates. Statistical significance was determined by Sidak’s multiple comparison test (two-way ANOVA). (**D**) Schematic representation of the NLS-EGFP reporter for measuring NE rupture. When the nucleus ruptures, the NLS-EGFP signal (green, in the nucleus) diffuses into the cytoplasm (out =  rupture). Illustration created with BioRender.com. (**E**) Representative images of MLL4^WT^ and MLL4^LoF^ MSCs expressing NLS-EGFP passing through constrictions of 3 μm. Dashed white lines highlight the microchannel borders. Yellow arrows indicate cells that are subjected to NE rupture. Scale bars, 10 µm. (**F**) NLS-EGFP normalized mean intensity inside the nucleus (blue) and in the cytoplasm (green) of MLL4^WT^ in case of NE rupture (2 μm) or not (3 μm) (*n* ≥ 4). Cytoplasmic NLS-EGFP intensity was normalized to initial nuclear intensity and vice versa to obtain NLS nuclear or cytosolic signal. Time equal to 0 (x-axis) corresponds to the tip of the nucleus reaching the end of the constriction in which NE rupture occurs. (**G**) Bar plots (mean + SEM of *n* = 3 independent biological replicates) showing the % of cells undergoing NE rupture in NLS EGFP- MSCs. Statistical significance was determined by Sidak’s multiple comparison test (two-way ANOVA). (**H**) Representative images of MLL4^WT^ and MLL4^LoF^ MSCs expressing cGAS-EGFP passing through constrictions of 3 μm. Scale bars, 10 µm. Dashed white lines highlight the microchannel borders. Yellow arrows indicate cGAS foci. (**I**) Bar plots (mean + SEM of *n* = 3 independent biological replicates) showing the % of cells showing cGAS condensates in cGAS-EGFP- MSCs. Statistical significance was determined by Sidak’s multiple comparison test (two-way ANOVA). (**J**) Representative images of MLL4^WT^ and MLL4^LoF^ MSCs co-expressing cGAS-EGFP and BAF-mCherry passing through constrictions of 3μm. Scale bars, 10 µm. Dashed white lines highlight the microchannel borders. On the right, quantification of the area of the cGAS-EGFP and BAF-mCherry foci (Bar plots - mean + SEM of *n* = 3 independent biological replicates). Statistical significance was determined by a two-tailed unpaired Student’s *t*-test. *P* values definition: *p < 0.05, **p < 0.01, ***p < 0.001, ****p < 0.0001, ns not significant (*p* ≥ 0.05). Source data are available online for this figure.

**Figure EV5:**
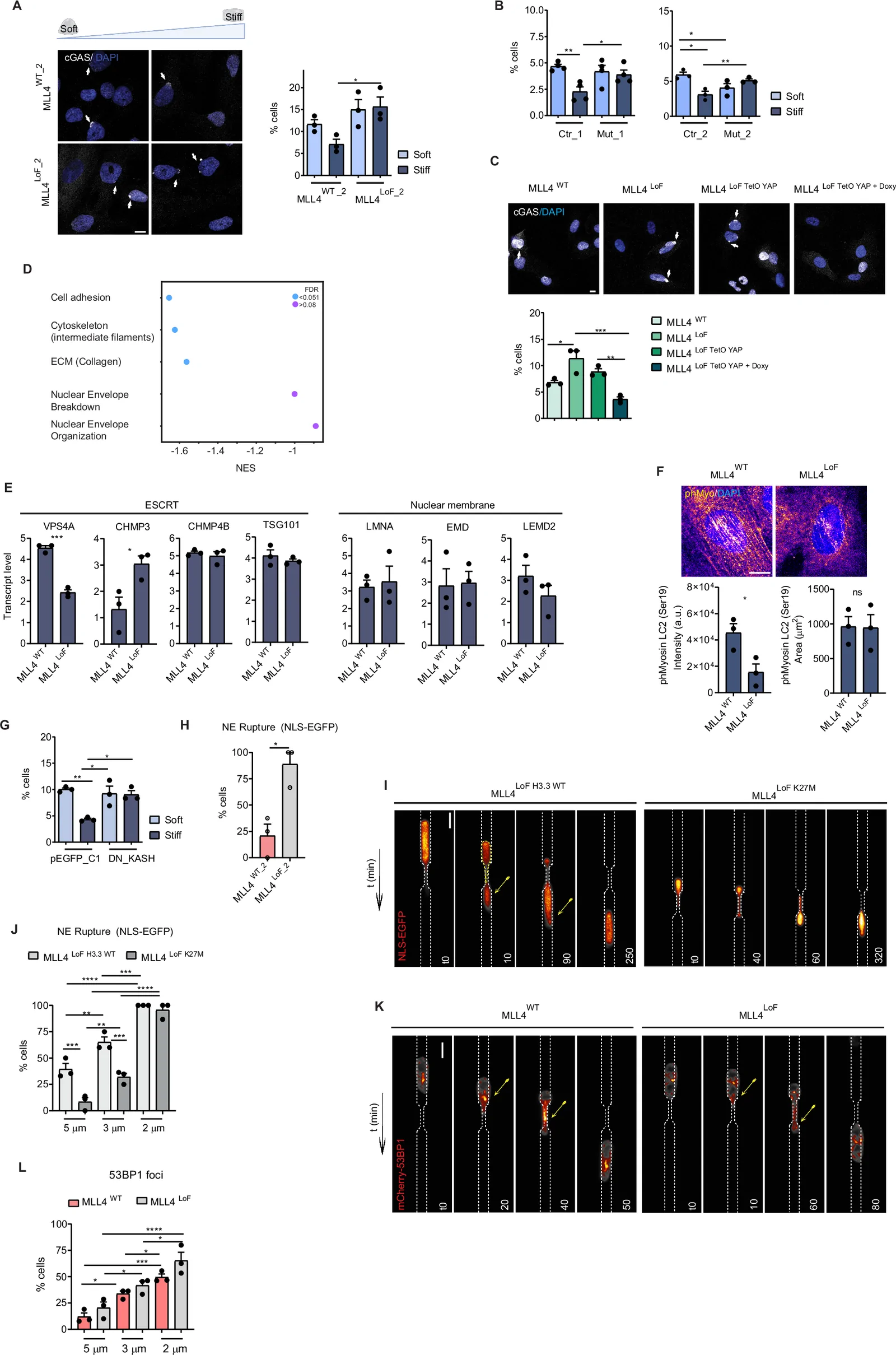
MLL4 LoF leads to recurrent NE rupture upon mechanical stress. (**A**) Representative confocal images of immunostaining for cGAS in MLL4^WT_2^ and MLL4^LoF_2^ MSCs independent clones on soft and stiff matrix. Scale bars, 10 µm. On the right, quantification of the percentage of cells showing cGAS accumulation at the nuclear periphery, indicating NE rupture (white arrows). Bar plots show mean + SEM of *n* = 3 independent biological replicates. Statistical significance was determined by Sidak’s multiple comparison test (two-way ANOVA). (**B**) Quantification of the percentage of cells showing cGAS accumulation at the nuclear periphery in primary fibroblasts from healthy donors (Ctrl) or Kabuki patients (Mut) on soft and stiff matrix, indicating NE rupture (white arrows). Bar plots show mean + SEM of *n* = 3 independent biological replicates. Statistical significance was determined by Sidak’s multiple comparison test (two-way ANOVA). (**C**) Representative confocal images of immunostaining for cGAS in MLL4^WT^, MLL4^LoF^, and MLL4^LoF^ MSCs overexpressing TetO YAP (±24 h doxycycline treatment) on stiff matrix. Scale bars, 10 µm. Below, quantification of % of cells showing cGAS accumulation at the nuclear periphery (white arrows). Bar plots show mean + SEM of *n* = 3 independent biological replicates. Statistical significance was determined by Tukey’s multiple comparison test (two-way ANOVA). (**D**) Bubble plot showing the normalized enrichment score (NES) for the specified FDR (false discovery rate) in the selected categories (GSEA analysis). (**E**) Real-time quantitative reverse transcription PCR of the indicated genes implicated in NE organization and repair in MLL4^WT^ and MLL4^LoF^ MSCs on stiff matrix (mean + SEM; *n* = 3 independent experiments each). Statistical significance was determined by a two-tailed unpaired Student’s *t*-test. (**F**) Representative confocal images of immunostaining for phMyosin Light Chain 2 (Ser19) in MLL4^WT^ and MLL4^LoF^ MSCs on soft stiff matrix. Scale bars, 10 µm. Below, quantification of phMyosin intensity and area (mean + SEM; *n* = 3 independent experiments). Statistical significance was determined by a two-tailed unpaired Student’s *t*-test. (**G**) Quantification of the percentage of cells showing cGAS accumulation at the nuclear periphery in MLL4^WT^ MSCs expressing pEGFP C1 or pEGFP C1 DN_KASH construct on soft and stiff matrix. Bar plots show mean + SEM of *n* = 3 independent biological replicates. Statistical significance was determined by Sidak’s multiple comparison test (two-way ANOVA). (**H**) Quantification of the percentage of cells undergoing NE rupture in NLS EGFP- MSCs (independent clones). Bar plots show mean + SEM of *n* = 3 independent biological replicates. Statistical significance was determined by a two-tailed unpaired Student’s *t*-test. (**I**) Representative images of MLL4^LoF^ H3.3WT and H3.3 K27M - NLS-EGFP in the process of passing through restrictions of 3 μm at the indicated time points. Scale bars, 10 µm. Dashed white lines highlight the microchannel borders. Yellow arrows indicate cells undergoing NE rupture. (**J**) Bar plots showing the percentage of cells undergoing NE rupture passing through constrictions of different widths (mean + SEM, *n* = 3). Statistical significance was determined by Sidak’s multiple comparison test (two-way ANOVA). (**K**) Representative images of MLL4^WT^ and MLL4^LoF^ MSCs expressing mCherry-53BP1 in the process of passing through restrictions of 3 μm at the indicated time points. Scale bars, 10 µm. Dashed white lines highlight the microchannel borders. Yellow arrows highlight cells with 53BP1 foci while passing through the constriction. (**L**) Quantification of the percentage of cells harboring 53BP1 foci (*n* = 3 independent experiments) passing through constrictions of different widths (5, 3, and 2 μm). Bar plots show mean + SEM. Statistical significance was determined by Sidak’s multiple comparison test (two-way ANOVA). *P* values definition: *p < 0.05, **p < 0.01, ***p < 0.001, ****p < 0.0001, ns not significant (*p* ≥ 0.05).

Considering that the MLL4-associated phenotype resembled the perturbation of the NE integrity that characterizes pathological conditions such as cancer and laminopathies (Raab et al, 2016; Denais et al, 2016; Earle et al, 2020), we measured the frequency of NE rupture in response to ECM stiffening. To assess the frequency of spontaneous NE rupture, we quantified the clustering of the endogenous cGAS, which senses the exposure of chromatin to the cytoplasm (Raab et al, 2016; Denais et al, 2016) (Fig. 6A). We found that ECM-mediated NE softening was mirrored by increased cGAS clustering in proximity to the NE. Of note, this pattern was resembled by the MLL4 LoF, as both MSCs and KS patient-derived fibroblasts grown on a stiff matrix showed an increment of spontaneous NE rupture (Figs. 6A and EV5A,B). Notably, by re-establishing YAP/TAZ nuclear abundance in the context of MLL4 LoF, we rescued cGAS clustering (Fig. EV5C). To rule out the mechanism by which MLL4 LoF contributed to NE fragility, we assessed the expression levels of ECM- and NE-associated genes. MLL4-associated condensates counterbalance Polycomb-mediated nuclear mechanical stress in Kabuki syndrome (Fasciani et al, 2020). We found that although the expression of genes implicated in NE functioning (ESCRT-III, complex, Lamins, LEM-associated and nucleopore proteins) were unaffected, genes involved in cell adhesion, cytoskeleton, and ECM were downregulated, as confirmed by a reduction of contractile perinuclear actin cap (Fig. EV5D–F). By interfering with the formation of a functional LINC complex through the overexpression of the dominant negative GFP–Nesprin-1/2–KASH protein, we found that cGAS clustering was depending on the balancing between the chromatin- and cytoskeleton-mediated mechanical forces acting on the NE (Fig. EV5G). The increased NE fragility of MLL4^Q4092X^ MSCs was further verified by monitoring the colocalization of cGAS and BAF, which is involved in the repair of the NE ruptures (Denais et al, 2016; Halfmann et al, 2019; Young et al, 2020; Guey et al, 2020). The redistribution of the BAF-mCherry in the proximity of the nuclear envelope occurred at the same sites in which cGAS clustered into condensates, irrespective of the source of NE fragility (Fig. 6B,C). To better determine the transient loss of NE integrity during nuclear deformation, we combined the NLS-GFP reporter system with the migration of MSCs through the confined environment of microchannels (Fig. 6D). By monitoring the relative accumulation of the GFP signal in the cytoplasm after the nuclear engagement with the microchannel restrictions (Fig. 6E,F; Movies EV4–6), we found that in wild-type MSCs the frequency of NE rupture increased with the augmented degree of confinement, in line with previous reports (Denais et al, 2016) (Fig. 6G). Of importance, this response was altered in the MLL4 LoF condition, as the NE rupture occurred in all the cells encountering the least stringent confinement (Figs. 6G and EV5H; Movies EV7–9). Notably, this pattern was rescued upon re-establishing the PcG condensates by means of expressing the H3.3K27M mutant (Fig. EV5I,J). To determine whether this transient NE rupture culminates in the exposure of the genomic DNA in the cytoplasm and eventually in the activation of the NE repair machinery, we monitored the clustering of both cGAS and BAF in living cells. We found that shortly after passing through the constrictions, the cGAS-GFP biosensor clustered in condensates, whose frequency increased proportionally to the degree of confinement (Fig. 6H,I, and Movies EV10–12). The MLL4^Q4092X^ MSCs showed an increased occurrence of cGAS clustering irrespective of the compressive forces on the nucleus (Fig. 6H,I and Movies EV13–15). Of note, the events of NE rupture were often associated with mechanically induced DNA damage, as visualized by the accumulation of 53BP1 after the passage through the constrictions (Fig. EV5K,L). Nevertheless, the MLL4^Q4092X^ MSCs did not show a higher frequency of DNA damage, thus suggesting that the genome stability is similarly preserved (Fig. EV5K,L). As the efficacy of NE repair is associated with the abundance and the extent of cytosolic BAF accumulating at the rupture site (Denais et al, 2016; Halfmann et al, 2019; Young et al, 2020; Guey et al, 2020), we monitored in living cells the dimensions of the BAF clusters at the NE rupture sites that were visualized by using the cGAS-GFP biosensor. We found that while the cGAS condensates had a comparable size, the dimension of the BAF clusters was reduced in MLL4^Q4092X^ MSCs (Fig. 6J). Together, these data indicate that in response to nuclear mechanical stress, the NE integrity is impaired in *KMT2*D haploinsufficient cells.

### Loss of NE integrity drives the cGAS/STING-mediated cell death in KS

**Figure 7:**
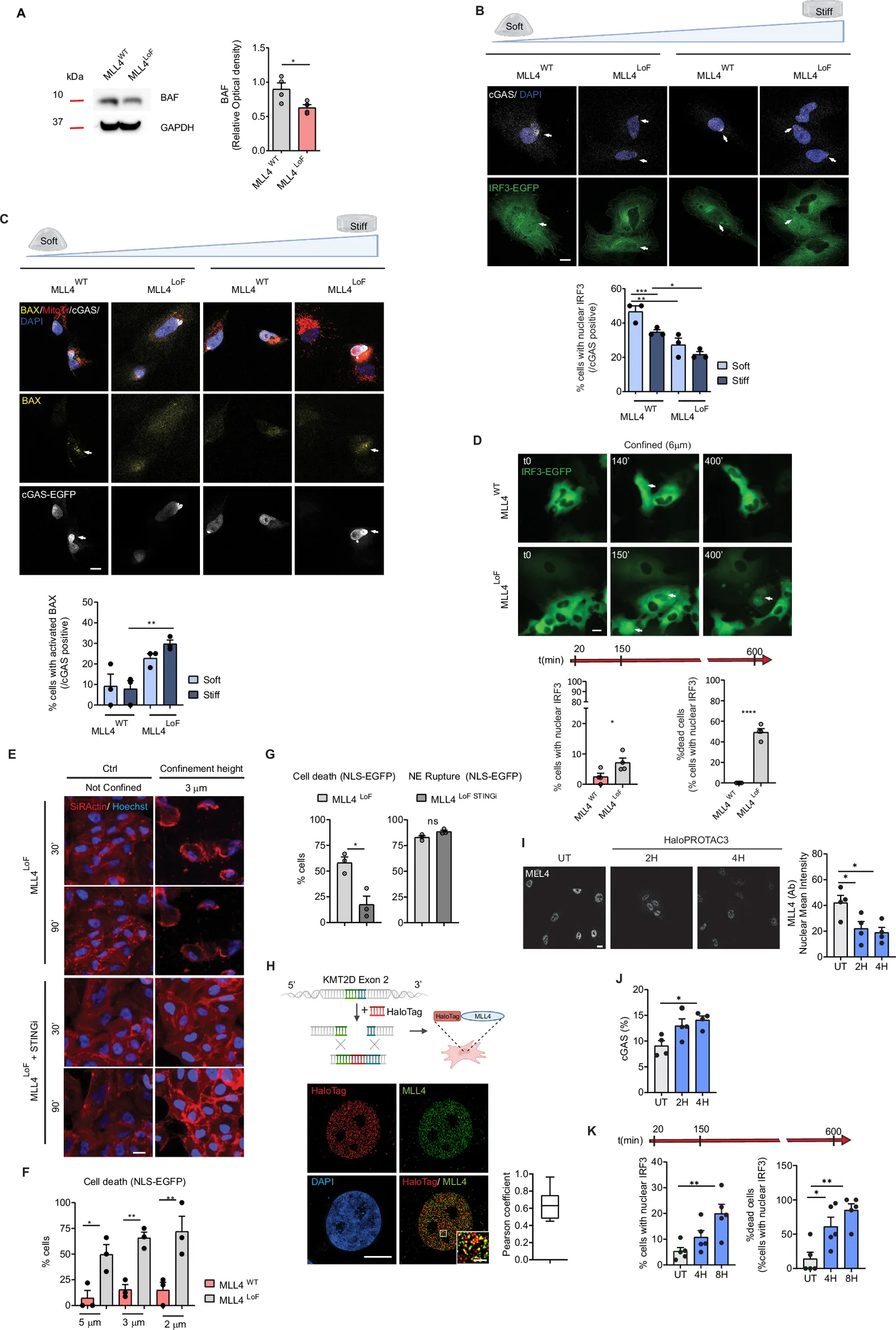
Loss of NE integrity drives the cGAS/STING-mediated cell death in KS. (**A**) Western Blot analysis of BAF in MLL4^WT^ and MLL4^LoF^ MSCs; GAPDH was used as a loading control. On the right: signal quantification for BAF normalized on GAPDH levels. Bar plots show mean + SEM of *n* = 4 independent experiments. Statistical significance was determined by a two-tailed unpaired Student’s *t*-test. (**B**) Representative confocal images of immunostaining for cGAS in MLL4^WT^ and MLL4^LoF^ MSCs overexpressing IRF3-EGFP on soft and stiff matrix. Scale bars, 10 µm. Below is a quantification of the percentage of cells harboring IRF3 nuclear localization calculated over the cGAS-positive cells. Bar plots show mean + SEM of *n *= 3 independent biological replicates. Statistical significance was determined by Sidak’s multiple comparison test (two-way ANOVA). (**C**) Representative confocal images of immunostaining for BAX (yellow) in MLL4^WT^ and MLL4^LoF^ MSCs overexpressing cGAS-EGFP (white) on soft and stiff matrix. The MitoTracker (red), was used to visualize mitochondria. Scale bars, 10 µm. Below, quantification of the percentage of cells with activated BAX measured over the cGAS-positive cells. Bar plots show mean + SEM of *n* = 3 independent biological replicates. Statistical significance was determined by Sidak’s multiple comparison test (two-way ANOVA). (**D**) Representative images of MLL4^WT^ and MLL4^LoF^ MSCs overexpressing IRF3-EGFP under cell confinement of 6μm. Scale bars, 10 µm. Below is a quantification of the percentage of cells harboring IRF3 nuclear localization in the first 150’ of time-lapse. On those cells having nuclear IRF3, the percentage of cells that undergo cell death within 600’ of time-lapse was calculated. Bar plots show mean + SEM (*n* = 4 combined from 2 independent experiments). Statistical significance was determined by a two-tailed unpaired Student’s *t*-test. (**E**) Representative images showing MLL4^LoF^ MSCs treated or not with the STINGi, not confined and under confinement of 3 μm at the indicated time points. Cell nuclei are stained with Hoechst (blue), and the cytoskeleton is marked in red (SirActin). Scale bars, 10 µm. (**F**) Bar plots (mean + SEM; *n* = 3 independent biological replicates) showing the % of cells undergoing cell death in NLS EGFP- MSCs passing through restrictions of different widths. Statistical significance was determined by Sidak’s multiple comparison test (two-way ANOVA). (**G**) Bar plots (mean + SEM; *n* = 3 independent biological replicates) showing the % of cells undergoing NE rupture and cell death in NLS EGFP- MSCs treated or not with the STINGi passing through restrictions of 3μm. Statistical significance was determined by a two-tailed unpaired Student’s *t*-test. (**H**) Representative SIM images of HaloTag-646 and MLL4 immunostaining in MLL4^HaloTag^ MSCs, scale bars 10 µm. On the right, the Pearson correlation coefficient is measured in *n* = 9 cells (one biological replicate). The box plot indicates median values (middle lines), first and third quartiles (box edges) and min to max percentiles (error bars). (**I**) Representative confocal images of MLL4 immunostaining in MLL4^HaloTag^ MSCs untreated (−) or treated (+) with HaloPROTAC3 for 2 and 4 h; scale bars 10 µm. Below, quantification of MLL4 nuclear Mean Intensity in the specified conditions; Bar plots show mean + SEM *n* = 4 independent biological replicates. Statistical significance was determined by a two-tailed unpaired Student’s *t*-test. (**J**) Quantification of the percentage of cGAS-positive cells in MLL4^HaloTag^ MSCs untreated (−) or treated (+) with HaloPROTAC3 for 2 and 4 h. Bar plots show mean + SEM *n* = 4 independent biological replicates. Statistical significance was determined by a two-tailed unpaired Student’s *t*-test. (**K**) Quantification of the percentage MLL4^HaloTag^ MSCs harboring IRF3 nuclear localization in the first 150’ of time-lapse, untreated (−) or treated (+) with HaloPROTAC3 for 4 and 8 h. On those cells having nuclear IRF3, the percentage of cells that undergo cell death within 600’ of time-lapse was calculated. Bar plots show mean + SEM on *n* = 5 independent biological replicates. Statistical significance was determined by a two-tailed unpaired Student’s *t*-test. *P* values definition: *p < 0.05, **p < 0.01, ***p < 0.001, ****p < 0.0001, ns not significant (*p* ≥ 0.05). Source data are available online for this figure.

**Figure EV6:**
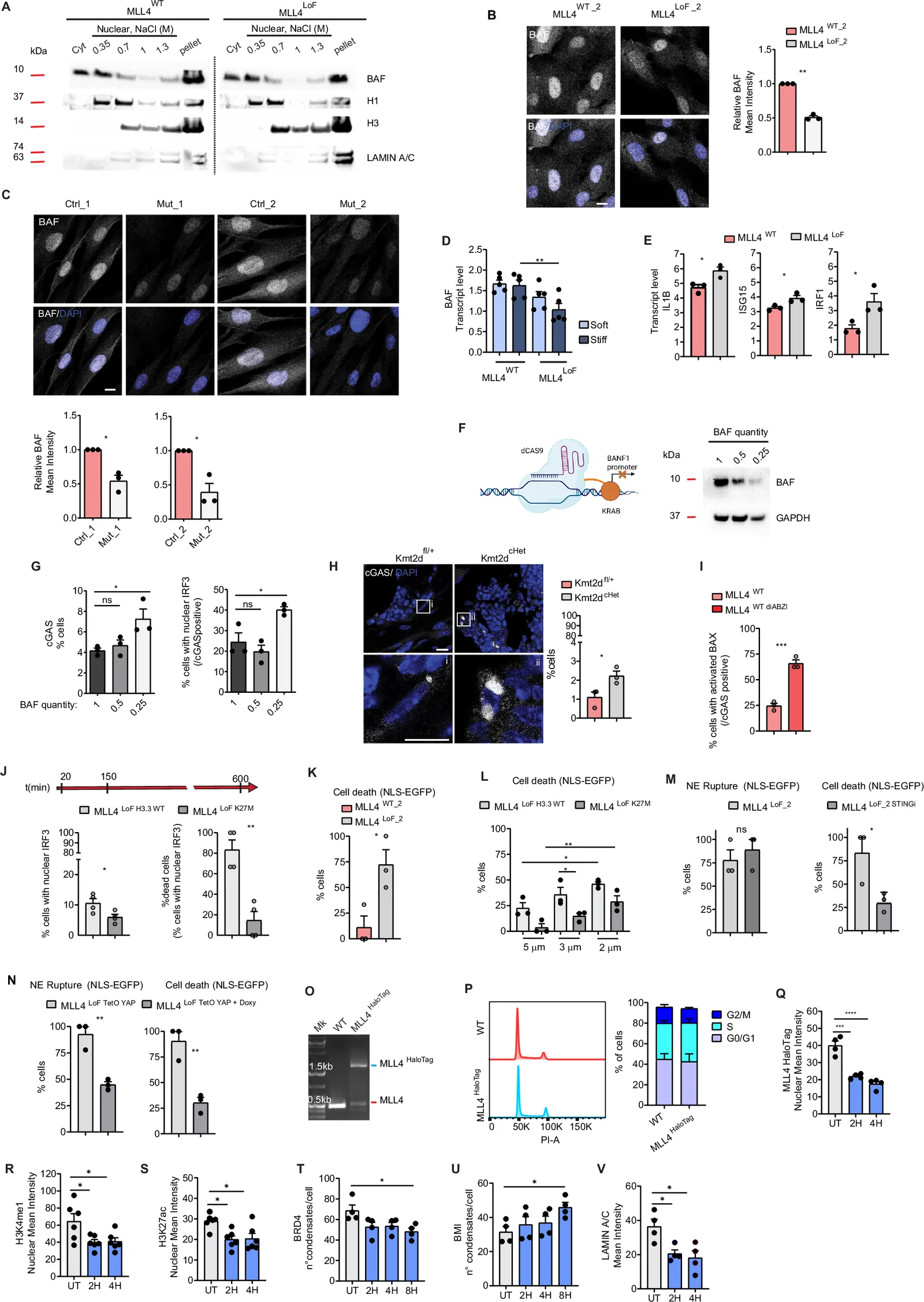
Disrupted NE integrity guides the cGAS/STING-mediated cell death in KS. (**A**) Western Blot analysis of BAF, Histone H1, Histone H3, and LAMIN A/C in MLL4^WT^ and MLL4^LoF^ MSCs extracts obtained using a gradient of NaCl. (**B**) Representative confocal images and e quantifications of nuclear BAF mean intensity in MLL4^WT_2^ and MLL4^LoF_2^ MSCs on the stiff matrix. Bar plots represent data quantification as mean + SEM of three independent experiments. Statistical significance was determined by a two-tailed paired Student’s *t*-test. Scale bar, 10 µm. (**C**) Representative confocal images and relative quantifications of immunostaining for BAF in primary fibroblasts from healthy donors (Ctrl) or Kabuki patients (Mut) on the stiff matrix. Bar plots represent data quantification as mean + SEM of three independent experiments. Statistical significance was determined by a two-tailed paired Student’s *t*-test. Scale bar, 10 µm. (**D**) Real-time quantitative reverse transcription PCR of BAF in MLL4^WT^ and MLL4^LoF^ MSCs on soft and stiff matrix. Bar plots show mean + SEM of *n* = 5 biological replicates. Statistical significance was determined by Sidak’s multiple comparison test (two-way ANOVA). (**E**) Real-time quantitative reverse transcription PCR of IL1B, ISG15 and IRF1 in MLL4^WT^ and MLL4^LoF^ MSCs on stiff matrix (*n* = 3 independent experiments each). Bar plots show mean + SEM of *n* = 3 independent replicates. Statistical significance was determined by a two-tailed unpaired Student’s *t*-test. (**F**) Left: schematic representation of the CRISPRi strategy adopted. The sgRNAs are designed to target the BANF1 promoter. Right: Western Blot analysis of BAF in MLL4WT MSCs transduced at different MOI of sgRNA targeting BANF1 promoter to obtain a gradient of BAF quantity (1 = SCRAMBLE, 0.5 = MOI 1, 0.25 = MOI 0.25). (**G**) Quantification of the percentage of cells showing cGAS accumulation at the nuclear periphery and harboring IRF3 nuclear localization (calculated over the cGAS-positive cells) in MLL4^WT^ expressing different levels of BAF protein. Bar plots show mean + SEM. of *n* = 3 biological replicates. Statistical significance was determined by Dunnett’s multiple comparison test (one-way ANOVA). (**H**) Representative confocal images of immunostaining for cGAS in the primary spongiosa of Kmt2d^fl/+^ and Kmt2d^cHet^ mice. Scale bar, 10 µm. On the right, quantification of the percentage of cells showing cGAS accumulation at the nuclear periphery. Bar plots show mean + SEM of data combined from three experimental groups. Statistical significance was determined by a two-tailed unpaired Student’s *t*-test. (**I**) Quantification of the percentage of cells with activated BAX measured over the cGAS-positive cells in MLL4^WT^ MSCs +/− treated with diABZI. Bar plots show mean + SEM of *n* = 3 independent experiments. Statistical significance was determined by a two-tailed unpaired Student’s *t*-test. (**J**) Quantification of the percentage of MLL4^LoF^ H3.3WT and H3.3 K27M- IRF3-EGFP harboring IRF3 nuclear localization in the first 150’ of time-lapse (6μm confinement). On those cells having nuclear IRF3 was calculated the percentage of cells that undergo cell death within 600’ of time-lapse (Bar plots = mean + SEM of *n* = 4 combined from two independent experiments; two-tailed unpaired student’s *t*-test. (**K**) Quantification of the percentage of cells undergoing cell death in NLS EGFP- MSCs independent clones passing through restrictions of 3μm. Bar plots show mean + SEM of *n* = 3 independent experiments. Statistical significance was determined by a two-tailed unpaired Student’s *t*-test. (**L**) Quantification of the percentage of cells undergoing cell death in MLL4^LoF^ H3.3WT and H3.3 K27M - NLS-EGFP passing through restrictions of 3 μm. Bar plots show mean + SEM. of *n* = 3 biological replicates. Statistical significance was determined by Sidak’s multiple comparison test (two-way ANOVA). (**M**) Quantification of the percentage of cells undergoing NE rupture and cell death in NLS EGFP- MLL4^LoF^ MSCs treated or not with the STINGi passing through restrictions of 3μm. Bar plots show mean + SEM of *n* = 3 independent experiments. Statistical significance was determined by a two-tailed unpaired Student’s *t*-test. (**N**) Quantification of the percentage of cells undergoing NE rupture and cell death in NLS EGFP- MLL4^LoF^ MSCs overexpressing TetO YAP (±24 h doxycycline treatment) passing through restrictions of 3 μm. Bar plots show mean + SEM of *n* = 3 independent experiments. Statistical significance was determined by a two-tailed unpaired Student’s *t*-test. (**O**) Analyses of genomic DNA to confirm the HaloTag insertion in MLL4 HaloTag MSCs. PCR products were run in 1% agarose gel to separate upper and lower bands corresponding to edited (2291 bp) and parental templates (600 bp), respectively. The PCR strategy established HaloTag insertion (1691 bp) in hemizygosity at the desired site after the knock-in (right part of the gel). (**P**) Representative histograms of cell cycle analysis (PI-A) and relative quantification of cell % of cell cycle phases in WT and MLL4 HaloTag MSCs (mean + SEM of *n* = 3 biological replicates). Bar plot represents mean + SEM of *n* > 4 biological replicates of nuclear mean intensity of the endogenous MLL4 HaloTag (**Q**), H3K4me1 (**R**) H3K27ac (**S**), LAMIN A/C (**V**) retrieved in untreated or treated MSCs^HaloTag^ with PROTAC3 for 2 and 4 h. Statistical significance was determined by a two-tailed unpaired Student’s *t*-test. Bar plot (Mean + SEM of *N* = 4 biological replicates) showing quantification of BRD4 (**T**) and BMI (**U**) number of condensates/cell retrieved in untreated or treated MSCs^HaloTag^ with PROTAC3 for 2, 4, and 8 h. Statistical significance was determined by a two-tailed unpaired Student’s *t*-test. *P* values definition: *p < 0.05, **p < 0.01, ***p < 0.001, ****p < 0.0001, ns not significant (*p* ≥ 0.05).

Besides nucleating the NE repair machinery, BAF also plays a central role in controlling the activation of the cGAS-STING pathway by restricting cGAS binding to the genome after NE rupture, preventing the assembly of DNA-cGAS oligomers and its enzymatic activity (Guey et al, 2020). Although the cellular distribution of BAF was not altered in MLL4^Q4092X^ MSCs, we found that its protein level was decreased (Figs. 7A and EV6A–D), possibly contributing to the activation of the cGAS-STING pathway upon mechanical stress. To verify this, we monitored the activation of the downstream effector IRF3 and found that in response to NE rupture, it was translocated into the nucleus (Fig. 7B). With the increase of ECM-mediated mechanical load, MLL4^Q4092X^ MSCs showed a higher frequency of cGAS-STING pathway activation (Figs. 7B and EV6E). Interestingly, recapitulating the 2-fold decrease in BAF1 abundance reflected in MLL4^Q4092X^ MSCs did not affect the cGAS-STING pathway response in MLL4WT MSCs, although further reduction of it mimicked the MLL4 LoF phenotype. (Fig. EV6F,G).

To figure out whether the cGAS-STING is primed by the increment of mechanical forces in a physiological context, we monitored the cGAS clustering along the growth plate and the primary spongiosa of long bones. We found that cGAS clustered in proximity to the NE of osteoblasts and osteoclasts residing in the primary spongiosa, which is characterized by the highest level of ECM stiffening (Figs. EV6H and 2E). Of importance, the frequency of cGAS activation resulted in being augmented in the *Kmt2d*^*cHe*t^ (Fig. EV6H).

These data suggested that the cGAS-STING pathway is activated under mechanical stress in the KS pathological setting. Besides mediating IFN-dependent inflammatory response, cGAS-STING signaling activates different evolutionarily ancient defense programs, including non-canonical autophagy and NLRP3-mediated inflammasome, which leads to different DNA-stimulated programmed cell death (PCD) pathways (Paludan et al, 2019; Gui et al, 2019; Murthy et al, 2020; Mazzocca et al, 2023). Indeed, we found that the executors of PCD pathways BAX/BAD (Paludan et al, 2019; Bové et al, 2014; Wang et al, 2017; Heilig et al, 2020) resulted in activation in MLL4^Q4092X^ MSCs grown on a stiff matrix (Figs. 7C and EV6I). By loading mechanical forces through cell confinement, we measure a dynamic increase of cGAS-STING pathways, which culminates with 50% of cell death upon MLL4 LoF (Fig. 7D). To determine the contribution of chromatin clustering unbalancing in the activation of the pathway, we re-established the PcG condensates by expressing the H3.3K27M mutant in MLL4^Q4092X^ MSCs and found that the level of IRF3 activation was rescued, and the consequent PCD was strongly inhibited (Fig. EV6J). Using the most stringent confined conditions (3 μm), we observed a pronounced induction of PCD, which was abrogated by inhibiting STING (Fig. 7E). To verify that under mechanical stress the cGAS-STING axis activated PCD pathways upon MLL4 LoF, we monitored cell death of MSCs challenged by the passages through the microchannel restrictions. We found that while wild-type MSCs rarely underwent cell death after migrating through the confined environments, many *KMT2D* haploinsufficient cells died in response to mechanical stress (Figs. 7F and EV6K). This response was partially rescued by re-establishing the proper chromatin compartmentalization of PcG proteins (Fig. EV6L). At the same time, while treatment with STING inhibitor did not reduce the frequency of NE rupture, it interfered with PCD pathways, increasing cell vitality of *KMT2D* haploinsufficient cells (Figs. 7G and EV6M). Notably, re-establishing YAP/TAZ nuclear abundance restored NE integrity and cell viability, in line with its role in controlling cGAS/STING activity (Sladitschek-Martens et al, 2022) (Fig. EV6N).

To rule out whether MLL4 contributes directly to tuning the chromatin condensates-mediated nuclear mechano-sensing and NE integrity modulating cGAS-STING signaling, we employed a HaloTag-based protein degradation strategy to temporally control its protein level. After retrieving MSCs in which we knocked in the HaloTag cassette in frame with *KMT2D* ORF in heterozygosity (named MLL4^HaloTag^), we firstly verified the maintenance of MLL4 protein distribution into chromatin condensates without perturbing cell viability (Figs. 7H and EV6O,P). Upon treatment of MLL4^HaloTag^ MSCs with HaloPROTAC3 for 2 h, we phenocopied the *KMT2D* haploinsufficiency by reducing MLL4 abundance of 50%, which resulted in a decrease of H3K4me1 and H3K27ac (Figs. 7I and EV6Q–S). In the same time frame, we measured a reduced clustering of transcriptional condensates, while PcG condensates resulted in being augmented with a lag time of 4 h (Fig. EV6T,U), possibly indicating the involvement of chromatin-associated downstream effectors. Nevertheless, MLL4 degradation resulted in a perturbation of nuclear shape and diminished Lamin A/C level (Fig. EV6V). The decrease of NE integrity resulted in the activation of the cGAS-STING pathway that triggered an increase in cell death of MLL4^HaloTag^ MSCs, in response to mechanical stress (Fig. 7J,K). Together, these findings indicated that in the KS condition, the increased frequency of NE rupture occurring in response to nuclear mechanical load, drove the activation of the cGAS-STING signaling, leading to PCD.

## Discussion

Genome compartmentalization into chromatin condensates has been viewed as a mean of increasing the efficacy and specificity of biochemical processes, including transcription regulation and DNA repair. Here, we show that the balance between transcriptional and PcG condensates is instrumental in tuning the nuclear mechanics and preserving nuclear integrity in response to mechanical load. This study goes beyond our previous understanding of the pathological contribution of chromatin compartmentalization to pathological settings (Fasciani et al, 2020), as we demonstrate that MLL4-dependent chromatin condensates dynamically respond to mechanical stress and actively contribute to nuclear mechano-responsiveness. Specifically, we found that MLL4 LoF hampers the nuclear response to external mechanical stimuli by reducing the assembly of transcriptional condensates. Of importance, the associated perturbation of the nuclear mechanical properties was reversed by restoring PcG condensate clustering, indicating that chromatin compartmentalization contributes to the mechanical response of the nucleus in coordination with externally applied forces. This response operates within a broader context shaped by ECM-cytoskeletal inputs and intranuclear processes. It is important to note that while the use of the H3.3 K27M mutant allowed us to probe the involvement of PcG-associated domains, this approach does not fully disentangle the epigenetic effects of PcG enzymatic activity from the biophysical and mechanical contribution of PcG condensates. These two mechanisms are likely interdependent, and the current lack of tools to separate them represents an inherent limitation of the study. Future efforts aimed at developing strategies to selectively perturb condensate organization without affecting catalytic activity (and vice versa) will be essential to fully dissect their individual contributions to nuclear mechanics and chromatin-based mechano-responsiveness. Indeed, several components of transcriptional condensates, including members of the MLL4 COMPASS-like complex, Mediator, and EP300/CREBBP co-activators, are enriched in Q-rich IDRs that favor the dynamic interaction with RNA Pol II transcription complex (Ruff et al, 2026). Whether these sequence features confer mechano-responsiveness to this subset of chromatin regulators should be tested experimentally, as we have shown here for the MLL4 PrLD through substitution of the polyQ tract. Nevertheless, with recent findings showing that chromatin folding influences nuclear stiffness, our results support the notion that chromatin-associated proteins are capable of perceiving and responding to mechanical forces to withstand these physical challenges.

We provided evidence that MLL4 functions as a chromatin mechano-sensor, transiently clustering into condensates in response to mechanical load. This responsiveness is mediated by the PrLD of MLL4, as mutagenizing the characterizing polyQ stretch impaired the mechano-stimulated clustering. Consistent with the mechanism of other mechano-sensors that rely on force-induced conformational changes (Beedle and Garcia-Manyes, 2023; Saini and Discher, 2019), our simulations indicate that MLL4-PrLD undergoes a transition from α-helix to β-strand conformation under pressure. While similar structural transitions underlie protein aggregation in polyQ disorders (Bunting et al, 2022; Priya and Gromiha, 2019), our results show that MLL4 condensate formation under confinement is disrupted by point mutations predicted to impair β-structure acquisition. Although mechanical signaling cascades likely contribute to chromatin reorganization (Venturini et al, 2020; Le et al, 2016; Gulen et al, 2023), our data support a model in which intrinsic biophysical properties of MLL4 enable condensate-mediated nuclear mechano-responsiveness. However, we cannot exclude that other MLL4-associated functions, including its methyltransferase activity, may cooperate in translating mechanical cues into nuclear and cellular phenotypes. To what extent other chromatin factors contributing to nuclear condensates may act as mechano-sensors is yet to be defined. Besides the anticipated role of conformational transitions, further investigation would be required to identify molecular signatures that confer mechano-sensing competence in chromatin regulators. Understanding these principles could uncover a broader paradigm linking chromatin organization into condensates and nuclear mechanics.

We also showed that the perturbation of condensates balancing predisposes to NE fragility, which is worsened by mechanically demanding conditions that cells experience during cell migration or in response to confinement. This vulnerability is biologically significant, as persistent NE rupture leads to cGAS-STING activation and programmed cell death. In the context of KS, MLL4 haploinsufficiency exacerbates nuclear deformation, increasing the frequency of NE ruptures and cGAS-STING activation that triggered PCD pathways. Sensing cytosolic DNA activates cGAS-STING, which stimulates a broad range of stress responses, including interferon signaling, non-canonical autophagy, or inflammasome activation that can eventually lead to cell death (Earle et al, 2020). What factors guide the cellular choice of activating PCD pathways downstream to cGAS-STING signaling have yet to be determined. Possibly, the extent of the stimulus, combined with the integration with other signaling pathways, may determine the cellular outcome. In this respect, we observed that in the context of KS, the relative abundance of BAF1 contributes to the activation of cGAS-STING, indicating that multiple factors can tune the pathway and the consequent biological outcome. Further experiments would be required to define whether alterations of the chromatin organization can predispose cells to mount a more robust response upon exposure to chromatin in the cytoplasm because of NE rupture.

Of importance, many of the clinical manifestations of KS are attributable to alterations in the nuclear mechanics and integrity of the nuclear envelope. Indeed, many affected tissues rely on a proper response to mechanical stimuli that guide cell lineage commitment during development or control proper tissue homeostasis and functionality postnatally. The latter condition is properly exemplified by the postnatal growth delay affecting KS individuals, which we showed to correlate with an altered endochondral ossification with osteoblasts being characterized by an increase of chromatin stiffening that was mirrored by an augmented activation of the cGAS-STING pathway. Whether a similar pattern contributes to determining other tissues’ functional alterations characterizing KS individuals warrants further investigation.

Our findings underscore the previously unrecognized role of chromatin condensates in nuclear mechano-sensing. By linking MLL4-dependent condensate dynamics to nuclear integrity and cGAS regulation, this study uncovers a novel mechanistic axis relevant for KS and potentially other diseases involving mechanical stress and nuclear fragility. Given recent studies implicating cGAS-STING in neurodegeneration, and the therapeutic efficacy of STING inhibitors in preclinical models (Robustelli et al, 2018; Xie et al, 2023), these results suggest that modulating chromatin mechanics or STING signaling may hold therapeutic potential for KS and related disorders with impaired mechano-responsiveness.

## Methods

**Table:** Reagents and tools table

Reagent/resource	Reference or source	Identifier or catalog number
**Experimental models**		
MSCs	Gifted from P. Tatrai	N/A
MLL4^Q4092X^ and MLL4^P4093X^ (MLL4^LoF_1^ and MLL4^LoF_2^ respectively)	Fasciani et al	N/A
Human primary fibroblasts	Provided by D. Genevieve	N/A
NIH3T3	American Type Culture Collection	CRL-1658
Mouse: Kmt2d ^fl/fl^	Gifted from I. Pastushenko (Y. Jang et al)	N/A
Mouse: CMV-Cre	Charles River Laboratories	JAX #006054
Mouse: Kmt2d^fl/+^, Kmt2d^cHet^	This paper	N/A
**Recombinant DNA**		
pTRIP-SFFV-EGFP-NLS	Addgene	#86677
pCDH-MiniNesprin1-GFP-cpstFRET	Gifted from Maiuri Lab	N/A
pCDH–EF1–MCS–IRES–PURO–H3.3/K27M	Gifted from Allis Lab	N/A
TETO-FUW-LMNA-HALO	Gifted from Zaret Lab	N/A
pTRIP-GFP-IRF3	Addgene	#127663
CMV_PBase	Gifted from Tiberi Lab	N/A
pTRIP-SFFV-cGAS-EGFP	Addgene	#127661
pTRIP-CMV-mCherry-53BP1	Addgene	#127658
pTRIP-BAF-mCherry	This paper	N/A
piggyBAC-MLL4-PrLD-HaloTag-Cry2	This paper	N/A
pKLV-U6gRNA(BbsI)-PGKpuro2ABFP	Addgene	#50946
pHR-SFFV-KRAB-dCas9-P2A-mCherry	Addgene	#60954
pET-28b-NLS-Cas9-2NLS-His	Gifted from Fava Lab	N/A
pCR-Blunt II-TOPO_Puro_HaloTag	This paper	N/A
piggyBAC-MLL4-PrLD L/A-HaloTag-Cry2	This paper	N/A
pUBC-SV40NLS-Pfv-Sapphire	Addgene	#203653
**Antibodies**		
Anti-Human MLL4	Invitrogen	Cat# 701869 RRID:AB_2633033
Histone H3	Cell Signaling	Cat# 9715 RRID:AB_331563
Anti-Human RING1B	Cell Signaling	Cat# 5694 RRID:AB_10705604
Anti-Human Lamin A/C JOL2	Abcam	Cat# ab40567 RRID:AB_775967
Anti-Human BMI1	Millipore	Cat# 05-637 RRID:AB_309865
Anti-Human BRD4	Abcam	Cat# ab128874 RRID:AB_11145462
H3K9me3	Abcam	Cat# ab8898 RRID:AB_306848
Anti-Human Lamin B	Abcam	Cat#ab65986
Anti-Human BAF	Custom; provided by Wiebe Lab (N.Ibrahim et al)	N/A
Pan-Histone H11-4	Millipore	Cat# MAB3422 RRID:AB_2114845
Anti-Human BAX (6A7)	Santa Cruz	Cat# sc-23959 RRID:AB_626728
Anti-Human cGAS	Cell Signalling	Cat# 79978 RRID:AB_2905508
H1	Neo Biotechnologies	Cat#3005-MSM7-P1
Anti-Human GAPDH (6C5)	Santa Cruz	Cat# sc-32233 RRID:AB_627679
H3K27ac Monoclonal Antibody	Invitrogen	#MA5-23516 RRID:AB_2608307
Anti-Human MLL4	Sigma-Aldrich	#HPA035977
Phospho Myosin Light Chain 2 Ser19	Cell Signaling	3675 RRID:AB_2250969
**Oligonucleotides and other sequence-based reagents**		
BRD4_FW	CGCTATGTCACCTCCTGTTTGC	N/A
BRD4_RV	ACTCTGAGGACGAGAAGCCCTT	N/A
RING1B_FW	CAGTCACAGCATTGAGGAAGGAC	N/A
RING1B_RV	GCTTCCTGATTGCTATGTGTGGA	N/A
BANF1_FW	GTTCTAGTGGCTTGAGGTATCC	N/A
BANF1_RV	CTTTTGGGAGGTTGTCATCTTG	N/A
MLL4_FW	TGAAAGGGCACTGAGGGATA	N/A
MLL4_RV	TGAGGGGGTGTAGGCAAG	N/A
BMI_FW	TCATTGTCTTTTCCGCCCG	N/A
BMI_RV	TCAGGTGGGGATTTAGCTCA	N/A
IL1B_FW	GGAGAATGACCTGAGCACCT	N/A
IL1B_RV	GGAGGTGGAGAGCTTTCAGT	N/A
ISG15_FW	CAACGAATTCCAGGTGTCCC	N/A
ISG15_RV	CAGAGGTTCGTCGCATTTGT	N/A
IRF1_FW	ATGCTTCCACCTCTCACCAA	N/A
IRF1_RV	TGTAGCTGCTGTGGTCATCA	N/A
VPS4A_FW	ACAACGTCAACCCTCCAGAA	N/A
VPS4A_RV	TGATAGCGTGGAGGAAGTACT	N/A
TSG101_FW	AGGAGGAAATGGATCGTGCC	N/A
TSG101_RV	TCAACCTCGGCTACTTCTTGA	N/A
CHMP4B_FW	TGGAGAAGAAAATCGAGCAGG	N/A
CHMP4B_RV	GCTGGAACTCGATGGTTGAT	N/A
CHMP3_FW	AGGTCAAGGAAGGCTGTGAG	N/A
CHMP3_RV	CCTCATGGTGGCCTGAATCT	N/A
LMNA_FW	GGTGACCTGATAGCTGCTCA	N/A
LMNA_RV	CGCAGCATCTCATCCTGAAG	N/A
EMD_FW	GAGGAGTGCAAGGATAGGGA	N/A
EMD_RV	TGAGCCATGAAGAGGAAGATGA	N/A
LEMD2_FW	GGAAGCCCAAGAATATATAGCCA	N/A
LEMD2_RV	GATGCCCACGTCCTTGTTAC	N/A
Alt-R® CRISPR-Cas9 crRNA	AGTCGGAGAGAGGGATGGAC	N/A
sg EXON2_FW	AGCGTATGTGTTTATCTGGGGAGGA	N/A
sg EXON2_RV	CACCAGACGCATGAGATTATCCAAAA	N/A
dsDONOR_FW	GTCTCCCCGAGAGGGCCCTGCCCAGTCGGAGAGAGGGGCCGCCACCATGatgaccgagta	N/A
dsDONOR_RV	CTGAATCTTTATCCTCACCAGCCAGCTTCTGGCTGTCGCCgctgccTccAccgccg	N/A
sg BANF1_1	CCTGGCTTGAACCATCACGG	N/A
sg BANF1_2	GGCCACCAAGAGGAAGCATG	N/A
HaloTag_FW	GCCGCTAGCATGGCAGAAATCGGTACTGG	N/A
HaloTag_RV	GCCACGCGTGCCGGAAATCTCGAGCG	N/A
BANF1_FW	GCCGGATCCATGACAACCTCCCAAAAGCACC	N/A
BANF1_RV	GCCCTCGAGTTATCACAAGAAGGCGTCGCACC	N/A
**Chemicals, enzymes and other reagents**		
Goat serum	Fisher Scientific	#11475055
BSA	Millipore	#126579
ProLong Gold	Invitrogen	#P36934
DAPI	Sigma-Aldrich	#D9542
SiR-actin	Spirochrome	#SC001
SiR-DNA	Spirochrome	#SC007
hematoxylin	Sigma-Aldrich	#H3136
paraformaldehyde	Sigma-Aldrich	#158127
H-151	MedChemExpress	#HY-112693
diABZI	MedChemExpress	#HY-112921B
ECL reagent	GE Healthcare	#RPN2232
TRIzol	Ambion	#15596018
polybrene	Millipore	# TR-1003-G
646 Janelia Fluor® HaloTag®	Promega	#GA1120
T4 Ligase	New England Biolabs	#M0202
Phusion HF 2×	New England Biolabs	#F531L
Phusion™ Plus DNA Polymerase	Thermo Scientific™	#F630S
Cas9	Purified in-house according to	From the plasmid “pET 28b-NLS-Cas9-2NLS-His”
NU7441	Selleck Chemicals	Cat#S2638
Puromycin	Invivogen	Cat# ant-pr-1
RO-3306	Sigma-Aldrich	#217699
HaloPROTAC3	Promega	#GA3110
Alt-R® CRISPR-Cas9 tracrRNA	IDT™	#1072533
Alt-R® Cas9 Electroporation Enhancer	IDT™	#1075916
Propidium Iodide	Invitrogen	#P3566
PureLink RNAse A	Invitrogen	#12091-021
**Software**		
Fiji (1.52)	NIH	https://imagej.nih.gov/ij/download.html
LAS AF Software 2.6.0	Leica Microsystems	N/A
Prims 9	GraphPad Software	N/A
Image lab 2.0.1	Bio-Rad	N/A
NIS elements (AR 5.11)	Nikon	N/A
BioRender	BioRender	https://biorender.com/
IDT^TM^ Custom Alt-R® CRISPR-Cas9 guide RNA	N/A	https://eu.idtdna.com/site/order/designtool/index/CRISPR_CUSTOM
CRISPOR	N/A	https://crispor.gi.ucsc.edu/
FlowJo™ v10	BD Science	N/A
Matlab R2025b	MathWorks	N/A
**Other**		
Lipofectamine 3000	Life Technologies	#L3000001
P1 Primary Cell 4D-NucleofectorTM X Kit L	Lonza	#V4XP-1024
PierceTM BCA Protein Assay Kit 24	Thermo Scientific	#23227
SuperScript III One-Step SYBR Green kit	Invitrogen	#11746

### Cell lines

The cell lines used in this study were hTERT-immortalized human adipose-derived MSCs (a gift from P. Tatrai) carrying frameshift mutation in the exon 39 of KMT2D, i.e., MLL4^Q4092X^ and MLL4^P4093X^ (MLL4^LoF_1^ and MLL4^LoF_2^, respectively), human primary fibroblasts derived from either healthy or Kabuki syndrome individuals (kindly provided by D. Genevieve), and NIH3T3 cells (American Type Culture Collection). MSCs were cultured in 1:1 DMEM/F-12 medium (Gibco; 11320-074) supplemented with 10% fetal bovine serum (Euroclone; ECS0180L) and 100 U/mL Penicillin/Streptomycin (Gibco; 15140122). Primary fibroblasts and NIH3T3 were maintained in DMEM medium (Gibco; 11965092) supplemented with 10% fetal bovine serum and 100 U/mL Penicillin/Streptomycin. Cells were maintained at 37 °C under 5% CO_2_. When needed, cells were treated with the STINGi (H-151 HY-112693) 0.2 µM for 24 h, the STING agonist (diABZI HY-112921B) 0.1 µM for 16 h, doxycycline 0.2 µg/mL for 24 h or HaloPROTAC3 (Promega GA3110) 500 nM for 2, 4, and 8 h.

### Mice

Mice were housed in a certified animal facility in accordance with European Guidelines. The experiments were approved by the Italian Ministry of Health as conforming to the relevant regulatory standards. Kmt2d^fl/fl^ mice were a kind gift from I. Pastushenko and were previously generated and described to target the SET domain of Mll4^46^. CMV-Cre mice (JAX #006054) were purchased from Charles River Laboratories. Control strain (Kmt2d^fl/+^) was generated by crossing Kmt2d^fl/fl^ with C57BL/6J mice. The KS mouse model (Kmt2d^cHet^) was obtained crossing Kmt2d^fl/fl^ with CMV-Cre mice to obtain Mll4 haploinsufficiency.

### Derivation of stable cell lines

MSCs expressing pTRIP-SFFV-EGFP-NLS (Addgene #86677), pCDH-MiniNesprin1-GFP-cpstFRET (gifted by Maiuri laboratory), pCDH–EF1–MCS–IRES–PURO–H3.3/K27M (gifted by Allis laboratory.), FUW-TetO-LMNA-HALO (gifted by Zaret laboratory), pTRIP-GFP-IRF3 (Addgene #127663), pTRIP-SFFV-cGAS-EGFP (Addgene #127661), pTRIP-CMV-mCherry-53BP1 (Addgene #127658), pTRIP-BAF-mCherry, FUW-TetO-wtYAP (Addgene #84009) were obtained through transduction of MSCs with the corresponding lentiviral vectors. MSCs stably expressing piggyBAC-MLL4-PrLD-HaloTag-Cry2 were obtained by co-nucleofecting 50,000 cells with piggyBAC-MLL4-PrLD-HALOTag-Cry2 (1 µg) and pCMV- Pbase (0.2 µg) (kindly provided by L. Tiberi) using P1 Primary Cell 4D-NucleofectorTM X Kit L (Lonza; V4XP-1024) Amaxa Nucleofector (program FF104, Lonza) following manufacturer’s instructions.

### CRISPR/Cas9 knock-in of HaloTag-MLL4 degron system

To knock-in the HaloTag cassette upstream of the MLL4 coding region, a double-stranded PCR product encoding the PuroR-T2A-HaloTag resistance was synthesized as previously described and used as a donor for the genome editing (Reagents and Tools table). MSCs were synchronized at the G2/M border through 16 h treatment with 8 µM of RO-3306 (Sigma-Aldrich, #217699). A total of 50,000 cells were then electroporated on a Lonza Nucleofector 4D according to the manufacturer’s instructions. Briefly, equal amounts of 100-µM crRNA and tracrRNA (Integrated DNA Technologies) were complexed to form gRNAs. About 150 pmol of gRNA were mixed with 120 pmol of Cas9 protein to form RNP as previously described (Ghetti et al, 2021). To enhance homologous recombination, 0.5 µM of NU7441 (Selleck Chemicals, Cat# S2638) was added to the fresh medium on day 1 and day 2 after the electroporation. Fully recovered MSCs after electroporation were kept under antibiotic selection with Puromycin (InvivoGen, Cat# ant-pr-1) for 10 days, before clonal selection. Genomic DNA was extracted, and the target locus amplified by PCR using Phusion High Fidelity DNA Polymerase (Thermo Fisher) with sgEXON2_FW and sgEXON2_RV oligos (Reagents and Tools table).

### FACS analysis

For cell cycle analysis, 80% confluent cells were detached with trypsin, pelleted and resuspended in 1 ml PBS 1X. About 3 ml of ice-cold 100% ethanol was poured dropwise. Fixed cells were stored at 4 °C for at least 1 h prior to staining. About 12 ml PBS was added to the samples to wash off the ethanol, and then pellets were resuspended in 50 µg/ml PI solution (Invitrogen; P3566) + 5 µg PureLink RNAse A (Invitrogen; 12091-021) in 3.8 mM sodium acetate and incubated in the dark for 30 min at 37 °C. All samples were acquired at a BD FACS Symphony A1 (BD Biosciences). Data were analyzed using FlowJo 10.10.0.

### Analysis of cell mechano-response

To assess the effect of different substrate stiffness on MSCs, we used the CytoSoft® Imaging 24-well Plates (Advanced Biomatrix; 5188-1EA) of 0.5 and 32 kPa stiffness. Plates were washed three times with PBS and then coated with 10 µg/ml fibronectin (Santa Cruz; sc-29011) for 1 h at RT. About 20,000 cells/cm^2^ were plated and grown for 48 h prior to analysis. For the optogenetics experiments, MSCs expressing piggyBAC-MLL4-PrLD-HaloTag-Cry2 were stained with 200 nM of 646 Janelia Fluor® HaloTag® (Promega; GA1120) diluted in complete culture medium for 20 min at 37 °C. The substrate was then quickly washed once in PBS before replacing the medium and performing time-lapse imaging.

To assess the level of rupture and cell death MSCs were plated in 35 mm Petri dish containing a set of molded microchannels, with variable dimensions and restrictions (4DCell; MC019). Microchannel dishes were washed three times with PBS for 5 min. The last washing was replaced in each chamber by 10 µl of 10 µg/ml fibronectin and incubated for 1 h at RT. After the coating, the device was washed three times with PBS, and the MSC culture medium containing 2% FBS was added to cover each chamber and left for 15 min at 37 °C. Afterward, cells (1,500,000 cells/10 µl in 2% FBS-containing medium) were loaded in each access port and kept for 30 min at 37 °C before adding 2 ml of 2% FBS culture medium to the dish. Cells were grown for 48 h. Six hours before live imaging, a gradient of FBS was created by adding a droplet of 10% FBS-containing medium in the access port closest to where the cells have been plated and with whom they communicate. Finally, 10% FBS-containing medium was added to fill each chamber, and time-lapse video microscopy was performed.

To confine cells in adhesion at 3 and 6 μm, the CSOW 620 – static confiner (4DCell) was used. Pistons and confinement slides were equilibrated for 1 h at 37 °C in the culture medium before performing live-cell imaging. To ensure cell adherence, the glass-bottomed six-well plate was coated with 10 µg/ml fibronectin for 1 h at RT, before plating 20,000 MSCs in the central circular part of the well. When needed, cells were stained with Hoechst and SiR-actin (Spirochrome; SC001). Confinement was performed 24 h after plating. To confine cells in suspension, cells were stained while in adherence with 200 nM of 549 Janelia Fluor® HaloTag® (Promega; GA1110) diluted in complete culture medium for 20 min at 37 °C. After destaining wash with PBS, cells were detached with trypsin and collected for proceeding with cell confinement. For each condition, 40,000 cells were seeded in the center of the glass-bottomed six-well plate. Immediately after seeding, cells were confined with the static confiner, followed by live imaging 5 and 30 min after confinement.

To study the NE tensional state, MSCs expressing pCDH-MiniNesprin1-GFP-cpstFRET were seeded at a density of 20,000 cells/cm2 in an IBIDI plate (82426) and stained with SirDNA (Spirochrome; SC007). Imaging was performed after 48 h. pCDH-MiniNesprin1-GFP-cpstFRET is an orientation-based FRET biosensor (circularly permutated stretch sensitive - cpst) that uses the N-terminus and C- terminus of Nesprin-1 protein as backbone. It was designed as previously described to be modulated by the angles between the donor (cpst Cerulean) and acceptor (cpst Venus).

### DNA constructs

For the optogenetic experiments, the construct piggyBAC-MLL4-PrLD-HaloTag-Cry2 was cloned as follows: the HaloTag sequence was PCR amplified and then cloned into the piggyBAC mCherry-MLL4-PrLD-Cry2 (Fasciani et al, 2020), substituting the mCherry construct.

To overexpress BAF, the lentiviral vector pTRIP-mCherry-BANF1 was generated as follows: the BANF1 sequence was PCR amplified from pHM-Hyg-1xFlag-BAF-WT (a kind gift from Matthew S. Wiebe). The fragment was then cloned into the lentiviral vector pTRIP-mCherry-53BP1, substituting the 53BP1 construct. The MLL4 PrLD L/A amplicon, in which all Leucine within the PolyQ tract were substituted with Alanine, was purchased from GenScript and subcloned into PB-MLL4-PrLD-HaloTag-CRY2 through FseI. Oligonucleotides used for PCR amplification of cloning fragments are listed in the Reagents and Tools table.

### Immunofluorescence

Cells were fixed with 4% PFA for 10 min at room temperature (RT) and washed three times with phosphate-buffered saline (PBS). Immunostaining was performed directly on the CytoSoft® Imaging 24-well Plates as follows: cells were permeabilized and blocked with PBS/1% bovine serum albumin (Millipore; 126579)/ 5% goat serum (Fisher Scientific; 11475055)/ 0.5% Triton X-100 (blocking solution) for 1 h at room temperature. Cells were incubated overnight at 4 °C with primary antibodies diluted in the blocking solution, washed in PBS three times, and incubated for 1 h at RT with secondary antibodies (Alexa Fluor conjugated produced in goat by Thermo Fisher) diluted in the blocking solution, containing DAPI (Sigma-Aldrich; D9542) for nuclear staining. After three washings with PBS, the mounting media ProLong Gold (Invitrogen; P36934) was added to each well and then covered with coverslips to preserve the staining. The primary antibodies used are listed in the Reagents and Tools table.

2D confocal images were acquired using a Leica TCS SP8 microscope with a HCX Plan Apo ×63/1.40 objective; 3D images were acquired with a Nikon Eclipse Ti2E AX laser scanning confocal with CFI Apo SR HP TIRF 100XC/1.49 objective. When needed (nuclear volume and flatness quantification), z stacks were acquired with sections of 0.1 μm (sampling rate calculated by the Nyquist theorem). Image acquisition settings were kept constant for downstream image analysis. SIM images were acquired using a Nikon Eclipse Ti2E N-SIM microscope in 2D using a CFI Apo SR HP TIRF 100XC/1.49 objective, with an ROI of 512×512 and 0.2 μm Z stacks.

### Live-cell imaging

Time-lapse video microscopy was performed at 37 °C and 5% CO2 using the Nikon Eclipse Ti2 with an ×20/0.75, ×60/1.4, or ×100/1.45 Plan Apo λ objective (Nikon) integrated with a Lumencor SpectraX LED light source system and an EMCCD sensor camera (Andor iXon Ultra 888) for the detection. When imaging cells in microchannels, each frame was acquired every 5–10 min for at least 12 h. When imaging cells under confinement, images were taken every 10 min for 2–10 h, starting the acquisition 20 min after applying the confiner. To avoid fluorophore bleaching and cell phototoxicity during time-lapse, imaging LED intensity and exposure time were kept between 4 and 25% and 5–100 ms, respectively. For the optogenetics experiments time-lapse video was carried out continuously for the indicated timings under controlled temperature and CO_2_. Images of fluorescent cells were acquired before and after the stimulus (blue-light activation with LED 470 100% intensity for 3 s) and then every 30 s. MSCs expressing pCDH-MiniNesprin1-GFP-cpstFRET were acquired using a Leica TCS SP8 confocal microscope with a HCX Plan Apo ×63/1.40 objective. Donor (cpCerulean) was excited at 458 nm, and emission peaks of cpCerulean and cpVenus were captured, respectively, in a window of 470–490 nm and 520–540 nm.

### Confocal Brillouin microscopy, data acquisition, and analysis

The Confocal Brillouin Microscope consists of an inverted Olympus IX-73 coupled to a double virtually imaged phased array (VIPA)-based spectrometer through optical fibers. The laser source (λ = 532 nm, VERDI) is focused on the sample plane via an Olympus UPlanSApo 60x/1.4 NA objective to have high resolution Brillouin shift maps of biological samples: a higher Brillouin shift is correlated with higher local stiffness, with a spatial resolution comparable to confocal ones (~400 nm on xy plane, ~800 nm on z). The 3D mechanical properties of the sample can be recovered in confocal laser scanning mode thanks to a pair of galvanometric mirrors (THORLABS) and a piezo stage (MadCity Labs). Standard brightfield and fluorescence units are located adjacent to the Brillouin laser-scanning module, allowing for the simultaneous comparison of morphological, fluorescent and mechanical data of any biological sample. The Microscope setup is controlled in MATLAB via a custom-built GUI. For the analysis of the nuclear mean Brillouin shift in cells and tissues stained with DAPI, a custom-made MATLAB program created a binary mask from fluorescence data and calculated the mean Brillouin shift across the masked region of each nucleus. For the analysis of the ECM mean Brillouin shift in tissues, another custom-made MATLAB code automatically segmented different regions of the maps, selected all the pixels having Brillouin shift values above 7.80 GHz (excluding the pixels corresponding to fluorescence masked regions), connected different parts of the images with the same shift and calculated the mean value across these regions.

### Atomic force microscopy (AFM)

Atomic force microscopy data were acquired with an NT-MDT Solver instrument, equipped with a tip-scanning SMENA head with accessories for data acquisition in fluids. Cells were seeded in 35 mm dishes with different stiffness substrates 24 h prior to data acquisition. Rectangular silicon CSG-11 cantilevers (NT-MDT) were used to acquire cell stiffness data, after calibration of their elastic constant by the Sader method in air. Data were taken along straight lines, then the raw force curves were calibrated and fitted using the Bilodeau model, suitable for a pyramidal tip indenter. Fitting routines, written in Octave [https://octave.org/], allowed the reconstruction of the cell profile and the elastic properties along the scanned line. Data were reported as the average and standard deviation of the elastic properties of the top 10% points of the height profile.

### Genetically encoded multimeric nanoparticles (NucGEMs)

For NucGEM experiments, cells were first transduced with NucGEM virus and incubated for a minimum of 72 h. Subsequently, 24 h before acquisition, the cells were seeded at a density of 5000 cells/cm² onto substrates of varying stiffness. Prior to image acquisition, cells were labeled with SiR-DNA for 30 min. Image acquisitions were performed using Nikon Eclipse Ti2 ×100/1.45 Plan Apo λ objective (Nikon) integrated with a Lumencor SpectraX LED light source system and a sCMOS sensor camera (Andor Zyla 5.5) for the detection. For the NucGEM tracking, cells were imaged using a 488 nm laser at 75% laser intensity with 100 ms exposure.

The analysis was performed using the Trackmate plugin in ImageJ to detect the spots and to connect spots as a track. The extracted trajectories were further analyzed in MATLAB using an adapted version of the code described in Mazza et al (Mazzocca et al, 2023). The code was used to calculate the mean squared displacement (MSD), anomalous diffusion exponent α, and generalized diffusion coefficient D. MSD curves were fitted using the anomalous diffusion model MSD ~ Dτ^α, where τ is the time lag, D is the generalized diffusion coefficient, and α is the anomalous diffusion exponent.

### Image analysis

Images acquired by confocal were analyzed using ImageJ (FIJI) software.

For 2D analysis, the DAPI signal was used to define the ROI (region of interest) of the nucleus. To measure the nuclear mean intensity and area, LIF files were first converted to TIFF composite images, then the minimum value of the threshold and the size range parameters were determined for each independent experiment to identify the nuclei on the binary image. For the measure of nuclear volume and flatness, a 3D analysis was performed by using the 3D plugin suite (ImageJ plugin) and segmenting the nuclei on the DAPI signal. Briefly, a Median 3D filter and 3D simple segmentation were applied to segment the nuclei, images were loaded on 3D Manager and the 3D features quantified (“Measure 3D/ Quantify 3D”). 3D rendering of nuclei was obtained by using the 3D viewer plugin (ImageJ). For measuring condensate size, intensity, and area in 2D, the analysis was performed in ImageJ as follows: first, background subtraction was applied (rolling ball correction), then unsharp masking and median filters were carried out. The condensates were finally identified with the Shanbhag dark automatic threshold.

For calculating the excess of perimeter (EOP), the ratio of internal and perimetral length of the NE (based on Lamin B staining) over the perimeter of the convex envelope was measured using an in-house ImageJ macro as previously published (Saini and Discher, 2019).

The percentage of cGAS-positive cells was measured by counting the number of cells showing cGAS accumulation at the nuclear periphery over the total number of cells acquired. The percentage of BAF-positive cells was measured by counting the number of cells showing BAF clustering (pTRIP-BAF-mCherry MSCs) over the number of cGAS-positive cells. The percentage of BAX-activated cells was measured by counting the number of cells showing BAX signal matching the MitoTracker signal over the number of the cGAS-positive cells (pTRIP-SFFV-cGAS-EGFP MSCs). The percentage of cells with nuclear IRF3 (pTRIP-GFP-IRF3 MSCs) was measured by counting the number of cells showing accumulation of IRF3 in the nucleus over the number of cGAS-positive cells. For the microchannel experiments, image analysis and single-cell tracking were carried out using the NIS software. Nuclear Envelope Rupture was quantified on the basis of the NLS-EGFP signal as previously described (Raab et al, 2016). For measuring the NLS-EGFP Intensity in the cytoplasm and nucleoplasm, a small ROI was put in front of the nucleus at each time frame in which the nucleus enters and passes through the restriction (ROI corresponding to the cytoplasm). The average intensity of the ROI of the cytoplasm was divided by the average intensity of the nuclear NLS-GFP signal before the nucleus entered the constriction (cytoplasm/nucleoplasm ratio) or vice versa (nucleoplasm/cytoplasm ratio). The percentage of rupture was calculated as the ratio between the number of cells undergoing nuclear envelope rupture and the number of cells passing through the constriction. The percentage of cell death was measured by calculating the ratio between the number of cells dying while passing through the restriction and the total number of cells passing through the constriction. The percentage of cells with cGAS, BAF, 53BP1, and MLL4-PrLD condensates was calculated based on the number of cells showing rounded clusters of the corresponding marker while passing through restrictions over the total number of cells that passed.

For the optogenetics experiments, the images of fluorescent cells were analyzed to obtain the number, area, and mean intensity of condensates as follows: maximum intensity projection in z was performed (z-step size of 0.5 μm), then the ROI was drawn based on the HaloTag-646 fluorescent intensity signal on the pre-stimulus condition. After background subtraction, in which the rolling ball correction was kept constant for every experiment, the following threshold was used to identify condensates: “(mean intensity of the nucleus on the pre-stimulus) + (4X standard deviation)”. This allowed us to consider variations in the expression of the protein before the stimulus at the single- cell level. The function “Find Maxima” with a prominence of 20 was used to further define the condensates. For the representative images, the out-of-the-nucleus background was subtracted, and the smart LUT was selected on ImageJ to allow clearer visualization of clusters.

The inverted FRET index was calculated as the ratio between cpCerulean and cpVenus mean intensity. Data were analyzed using the SirDNA channel to define the nuclear ROI. For the representative images, the PixFRET ImageJ plug-in was used to retrieve FRET/Donor maps.

For the colocalization analysis, the ImageJ function Colocalization was used. For each staining, the DAPI signal was used to identify the nucleus as an area of interest on which the Pearson coefficient was determined. Quantification of the nuclear to cytosolic ratio of YAP/TAZ or MLL4 was performed as previously described (Fasciani et al, 2020), using an adapted MATLAB routine that is deposited in GitHub. Briefly, the DAPI channel was used to segment nuclei and measure the average nuclear intensity of the channel of interest. To estimate the average cytosolic intensity per cell, a ring of 30-pixel width around each segmented nucleus was found. The nuclear to cytosolic intensity (NCI) was calculated as the ratio of the nuclear and cytosolic average YAP/TAZ or MLL4 intensities. For MLL4, we further normalized the NCI obtained by summing the average intensity of the nucleus plus the ring, identifying the cytosol.

### RNA extraction and expression level quantification

Total RNAs were extracted from log-phase cells with TRIzol (Ambion #15596018), according to the manufacturer’s instructions. Quantitative real-time PCR analysis was performed with SuperScript III One-Step SYBR Green kit (Invitrogen #11746) and C1000 Touch thermocycler—CFX96 Real Time System (Bio-Rad). RNA starting quantity was determined using the standard curve method. Primers used for gene expression analysis are listed in the Reagents and Tools table.

### Gene set enrichment analysis (GSEA)

GSEA was performed using a ranked gene list derived from differential expression analysis between MLL4 loss-of-function (LoF) and MLL4 wild-type (WT) conditions. Gene expression data (Log2 fold changes) were obtained from publicly available RNA-seq datasets reported in Fasciani et al (Fasciani et al, 2020). Where multiple transcripts encoded the same gene product, expression values were summed to obtain a unique value per gene symbol. Genes were ranked based on Log2 fold change (MLL4 LoF vs. WT) and input as a ranked list in .rnk format for compatibility with the GSEA PreRanked tool. Gene sets were obtained from the Molecular Signatures Database (MSigDB v2024.1), using the c2.cp.reactome.v2024. symbols and symbol collections in .gmt format (McCreery et al, 2025). The analysis was performed using GSEA v4.3.3 (Broad Institute), in PreRanked mode, with the following parameters: number of permutations = 1000; Enrichment statistic: weighted; min/max gene set size: 50/1000. For each gene set, a normalized enrichment score (NES) was calculated based on the distribution of the enrichment score (ES) under the null hypothesis. Significance was assessed via permutation testing, and gene sets with a false discovery rate (FDR) *q* value <0.051 were considered significantly enriched. Enrichment plots were generated for top-ranking gene sets using the GSEA visualization module. Additional split graphs of key genes within enriched pathways were generated using the Python package seaborn 0.13.2.

### Protein extraction and Western blot (WB) analysis

Total protein extracts were obtained in RIPA Buffer (10 mM Tris pH8, 1 mM EDTA, and 140 mM NaCl) by sonicating in pulses of 30 s with 30 s intervals by using the Bioruptor ® Pico sonication device. Lysates were cleared by centrifugation, and the supernatant was collected. Protein concentration was determined using PierceTM BCA Protein Assay Kit 24 (Thermo Scientific, 574 #23227), according to the manufacturer’s instructions. For nuclear fractionation, an Extraction Buffer (EB: 20 mM HEPES pH7.9, 25% glycerol, 1.5 mM MgCl_2_, 0.2 mM EDTA, 0.2 mM PMSF, 0.1 M DTT, and 0.1% Triton X-100) with increasing salt concentration ranging from 0.35 to 1.3 M NaCl was used. For western blot analysis, 30 μg of total protein extracts and the same volume of cytosolic/nuclear fractions were loaded onto a gradient gel (NuPAGE™ 4 to 12%, Bis-Tris, Invitrogen; NP0322BOX) run in MES Buffer (Invitrogen; NP0002). Proteins were transferred to a nitrocellulose membrane that was subsequently blocked in PBS-Tween containing 5% milk for one hour at RT and incubated with primary antibody O/N at 4 °C. Incubation with HRP- conjugated secondary antibody was performed for one hour at RT. The chemiluminescent signal was captured using the ChemiDoc XRS+ System (Bio-Rad). When needed, relative optical density was quantified with FIJI. Primary antibodies used are listed in the Reagents and tools table.

### Epigenome editing

The two guide RNA (gRNA) sequences targeting the BANF1 gene were designed according to the CRISPick portal (https://portals.broadinstitute.org/gppx/crispick/public). The guide RNA (gRNA) sequences are listed in the Reagents and Tools table. The two gRNAs were tandem cloned into the lentiviral vector pKLV-U6gRNA(BbsI)-PGKpuro2ABFP (Addgene #50946). For CRISPRi of the BANF1 gene, MSC wild-type cells were first transduced with the lentiviral construct pHR-SFFV-KRAB-dCas9-P2A-mCherry (Addgene #60954) at MOI 1 and with the lentiviral gRNA expression vector, pKLV-U6gRNA(BbsI)-PGKpuro2ABFP expressing the gRNAs of interest at MOI 0.25-1. Control cells were transduced with pKLV-U6gRNA(BbsI)-PGKpuro2ABFP carrying a non-targeting sgRNA pair. Lentiviral transduction was performed in the presence of polybrene (Millipore, 8 µg/ml).

### Animal studies

Mouse body growth and length were monitored at selected time points from postnatal day 14 (P14) to P30. To obtain the measures of mouse body length, mice were anesthetized with 5% Isoflurane and recorded with a camera. A single frame was isolated in post-processing, and body length from nose to tail was measured using ImageJ (FIJI) software. Animals were sacrificed at P30 for subsequent histological analysis.

### Histological examination of mouse tissues

Mice were euthanized using CO_2_ and cervical dislocation. Tibiae and femurs were collected and fixed with 4% PFA for 48 h. Tissues were cryopreserved in 30% sucrose overnight, embedded in Frozen Section Compound (Leica; 3801480) and sectioned at 30 µm using Thermo Scientific HM525 NX cryostat. For immunofluorescence, frozen sections were initially stained with Phalloidin-TRITC to visualize f-actin, post-fixed with 4% PFA and then incubated with 10 mM sodium citrate, pH 6.0 for 20 min at 95 °C. Incubation with blocking solution, primary and secondary antibodies were performed as previously described for the in vitro analysis. Sections were mounted using aqueous mounting medium and acquired using a Leica TCS SP8 confocal microscope with HC Plan Apo ×20/0.75 or HCX Plan Apo ×63/1.40 objective. For H&E staining and histological analysis, samples were acquired with the Zeiss Imager M2 microscope with ZEISS Axiocam 705 color camera and EC Plan-Neofluar ×20/0.5 objective. For femurs, longitudinal growth plate sections were used, and proliferative zone, hypertrophic zone, and total growth plate heights were measured using ImageJ (FIJI) software in at least six sites per section (1 section per mouse).

### Molecular dynamics simulation

Atomistic MD simulations of the extended polyglutamine (polyQ) tract belonging to the intrinsically disordered, Prion-like domain of MLL4 (MLL4-PrLD) (Fasciani et al, 2020) were carried out with the Gromacs 2020.3 software (https://manual.gromacs.org/documentation/2020.3/index.html), in combination with the a99SBdisp force field/TIP4P explicit water model (Essmann et al, 1995). Putative coordinates of MLL4-PrLD were obtained via the IntFold web server (https://www.reading.ac.uk/bioinf/IntFOLD/), whereby we extracted the structure of the polyQ stretch (i.e., residues 338 to 414 - Fig. 4A). The latter was solvated within a rhombic dodecahedral box and neutralized by an excess concentration of monovalent salt [KCl] = 0.15 M. Periodic boundary conditions in the three dimensions were associated with the particle mesh Ewald calculation of the full-system electrostatics (Hess et al, 1997), and a 12 Å cutoff was established to both Van der Waals and short-range electrostatic forces. In addition, LINCS constraints (Bussi et al, 2007) were applied to all hydrogen atoms, thereby allowing for an integration timestep of 2 fs.

Prior to raw data collection, a minimization/thermalization protocol has been followed that involves (i) 5000 minimization steps by a steepest descent algorithm, (ii) a 0.5-ns thermalization of the solvent bath in the NVT ensemble at T = 310 K, and (iii) a 5-ns further equilibration in the NPT ensemble at P = 1 atm—the last two stages restraining the coordinates of the solute. Fixed T, P were enforced by a stochastic velocity-rescale thermostat^71^ (*t* = 0.1 ps) and an isotropic Parrinello-Rahman barostat (Parrinello and Rahman, 1981) (*t* = 2.0 ps) respectively. Thus, subsequent 1-μs MD simulations of the polyQ tract at increasing target pressures—i.e., 1, 1.25, 1.5, and 1.75 atm—have followed, each trajectory starting from the last frame of the preceding stage on the pressure “ramp”. Yet, to ensure the stability and convergence of the system, each stage has been preceded by a 5-ns equilibration of the solvent bath in the NPT ensemble—as done similarly in the aforementioned protocol.

Lastly, raw data analyses and graphical rendering of the MD trajectories were performed with the VMD software (https://www.ks.uiuc.edu/Research/vmd/), whereas scientific Python libraries were employed for the elaboration and plotting of data.

### Quantifications and statistical analysis

Data were collected using LAS AF Software 3.5.7 for confocal imaging, NIS Elements AR 5.11.01 for time-lapse experiments, Image lab 2.14.0 software for acquisition of western blot images. For data analysis, Fiji (2.14.0) was used for immunofluorescence data analysis, NIS elements (AR 5.11.01) for time-lapse experiments analysis. No statistical method was used to predetermine sample size. All experiments were performed on independent biological samples. The average obtained for each replicate was used as the statistical “*n*”. The exact statistical parameters used for each experiment are reported in the figure captions. The statistical tests performed are two-tailed unpaired Student’s *t*-test and one or two-way ANOVA for multiple comparisons. To assess the effects of genotype, stiffness, and their interaction on area, excess of perimeter, flatness, volume and stiffness matrix, we employed linear mixed-effects models using the statsmodels Python package. Here, genotype and stiffness were treated as fixed effects, while replicates were considered as a random intercept to account for repeated measurements. Model fitting was performed using restricted maximum likelihood (REML). We report the *p* values associated with interaction terms (genotype × stiffness) to identify context-specific genotype effects across stiffness conditions. *P* values are indicated in the figures and the corresponding figure captions as: *p < 0.05, **p < 0.01, ***p < 0.001, ****p < 0.0001, ns not significant (*p* ≥ 0.05). Data collection and analyses of all studies involving animals were conducted randomly without blinding.

## Supplementary information

Appendix

Peer Review File

Movie EV1

Movie EV2

Movie EV3

Movie EV4

Movie EV5

Movie EV6

Movie EV7

Movie EV8

Movie EV9

Movie EV10

Movie EV11

Movie EV12

Movie EV13

Movie EV14

Movie EV15

Figure 1a

Figure 1b

Figure 1c

Figure 1d

Figure 1e

Figure 1f

Figure 1g

Figure 1h soft

Figure 1h stiff-250

Figure 1h soft-500

Figure 1h stiff-500

Figure 1h soft-750

Figure 1h stiff-750

Figure 1h soft-1000

Figure 1h stiff-1000

Figure 1h soft_sirDNA

Figure 1h stiff-sirDNA

Figure 1h filtered tracks

Figure 1i

Figure 1j

Figure 1k

Figure 2a

Figure 2a C1

Figure 2a MLL4-Phalloidin-Histone

Figure 2a C3

Figure 2b

Figure 2c

Figure 2d

Figure 2e

Figure 2f

Figure 2g

Figure 3a

Figure 3b

Figure 3c

Figure 3d

Figure 3e

Figure 3f

Figure 3g

Figure 3h

Figure 3i

Figure 3J-1

Figure 3j-2

Source data Fig. 4G

Figure 5a-soft

Figure 5a-stiff

Figure 5b

Figure 5c

Figure 5d

Source data Fig. 5E

Figure 5f-soft

Figure 5f-stiff

Figure 6a

Figure 6b

Figure 6c

Figure 6d

Figure 6e NLS-GFP

Figure 6e-LoF-NLS-GFP

Figure 6e WT-BF

Figure 6e_LoF_frames_1-60

Figure 6e_LoF_frames_61-120

Figure 6e_LoF_frames_121-181

Figure 6e_WT_frames_1-55

Figure 6e_WT_frames_56-110

Figure 6e_WT_frames_111-169

Figure 6f

Figure 6g

Figure 6h-LoF

Figure 6h_WT

Figure 6i

Figure 6j

Figure 7a-c

Figure 7d

Figure 7e

Figure 7f-k

Expanded View Figures

## Data Availability

All newly generated materials and data associated with this study (stable cell lines, mouse lines, and plasmids) will be provided upon request to the lead contact. The source data of this paper are collected in the following database record: biostudies:S-SCDT-10_1038-S44318-026-00884-z.
