# Supplementary material for: Chromatin condensates tune nuclear mechanosensing and preserve nuclear integrity to prevent cGAS activation
Source: EMBO J. 2026 Aug 12;45(18):6384–420. doi: 10.1038/s44318-026-00884-z (PMC13584762; doi:10.1038/s44318-026-00884-z)
Supplement: Supplementary file 1 [file 44318_2026_884_MOESM1_ESM.pdf]

Appendix for "Chromatin condensates tune nuclear mechanosensing and preserve nuclear integrity to prevent cGAS activation"

D'Annunzio S. *et al*

Table of Contents

Appendix Figure S1 1

Appendix Figure S2 2

Appendix Figure S3 3

Appendix Figure S4 4

Appendix Figure S1. Western Blot analysis of MLL4 in soft and stiff conditions.

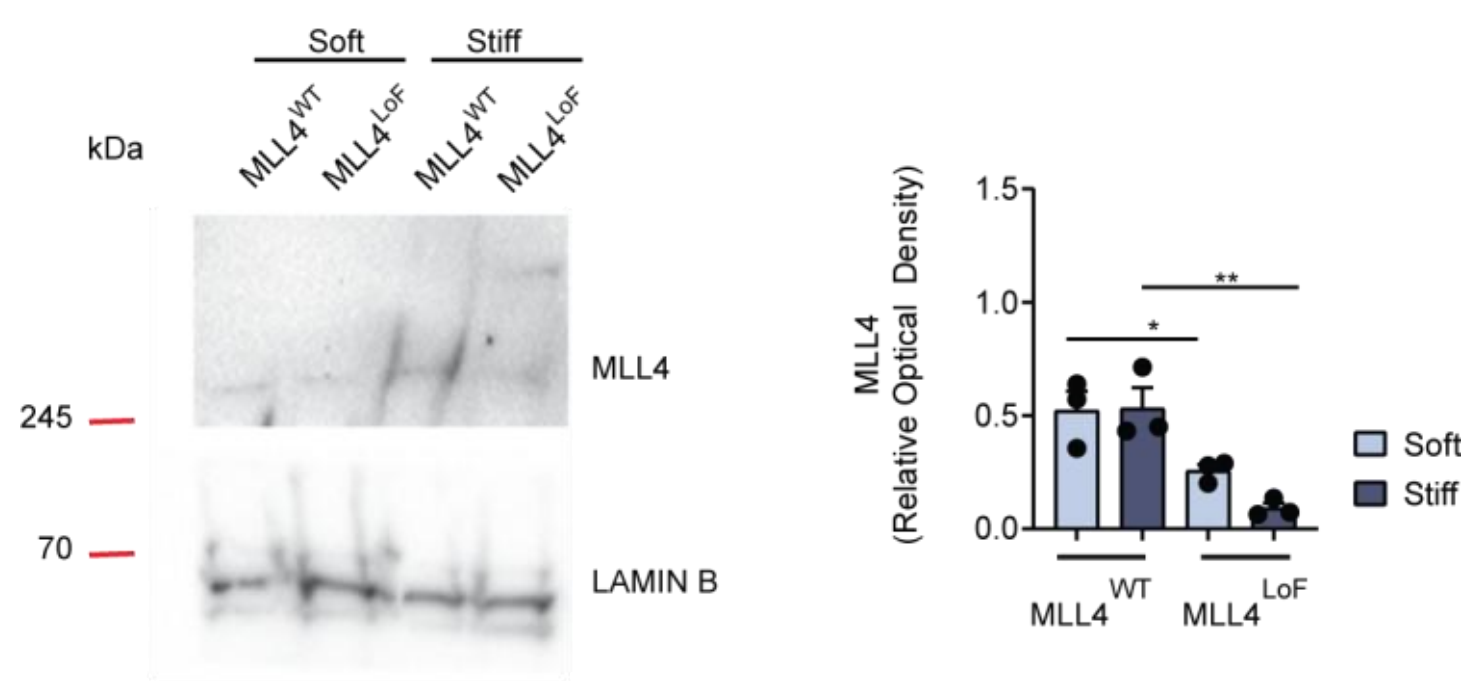

Western Blot analysis of MLL4 and LAMIN B in MLL4^{WT} and MLL4^{LoF} MSCs plated in soft and stiff matrix (nuclear extracts). On the right, signal quantification of MLL4 normalized on LAMIN B1 levels. Bar plots show Mean + SEM of n=3 independent experiments. Statistical significance was determined by Sidak's multiple comparison test (two-way ANOVA).

Appendix Figure S2. Analysis of nuclear (Nu) and cytosolic (Cyt) MLL4 average fraction.

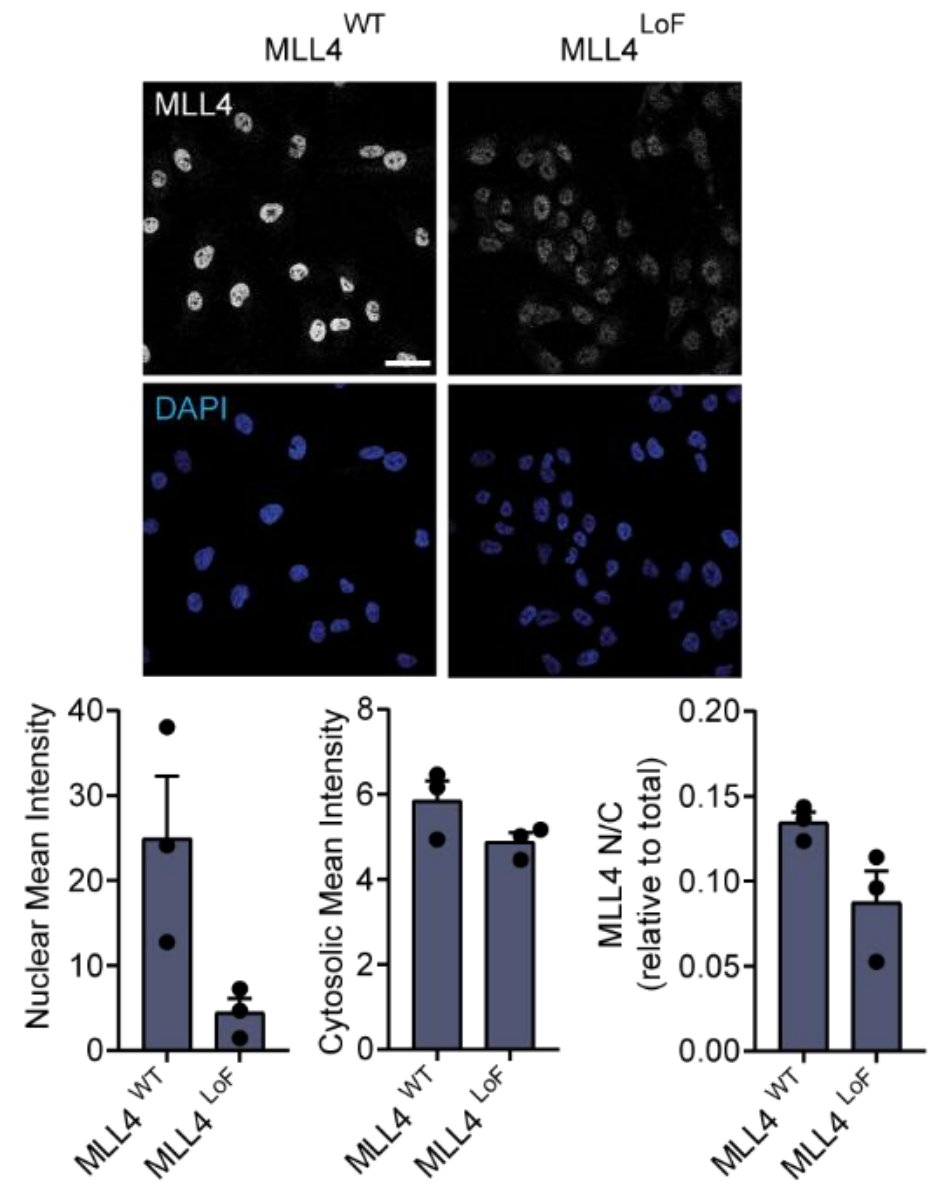

Representative confocal images and relative quantification of nuclear (Nu) and cytosolic (Cyt) MLL4 average fraction in MLL4^{WT} and MLL4^{LoF} MSCs in stiff conditions. Scale bars, 20 µm. Bar plots show Mean + SEM of n=3 independent biological replicates. A two-tailed unpaired student's t-test determined statistical significance.

Appendix Figure S3. Atomic force microscopy (AFM) measurements of nuclear stiffness.

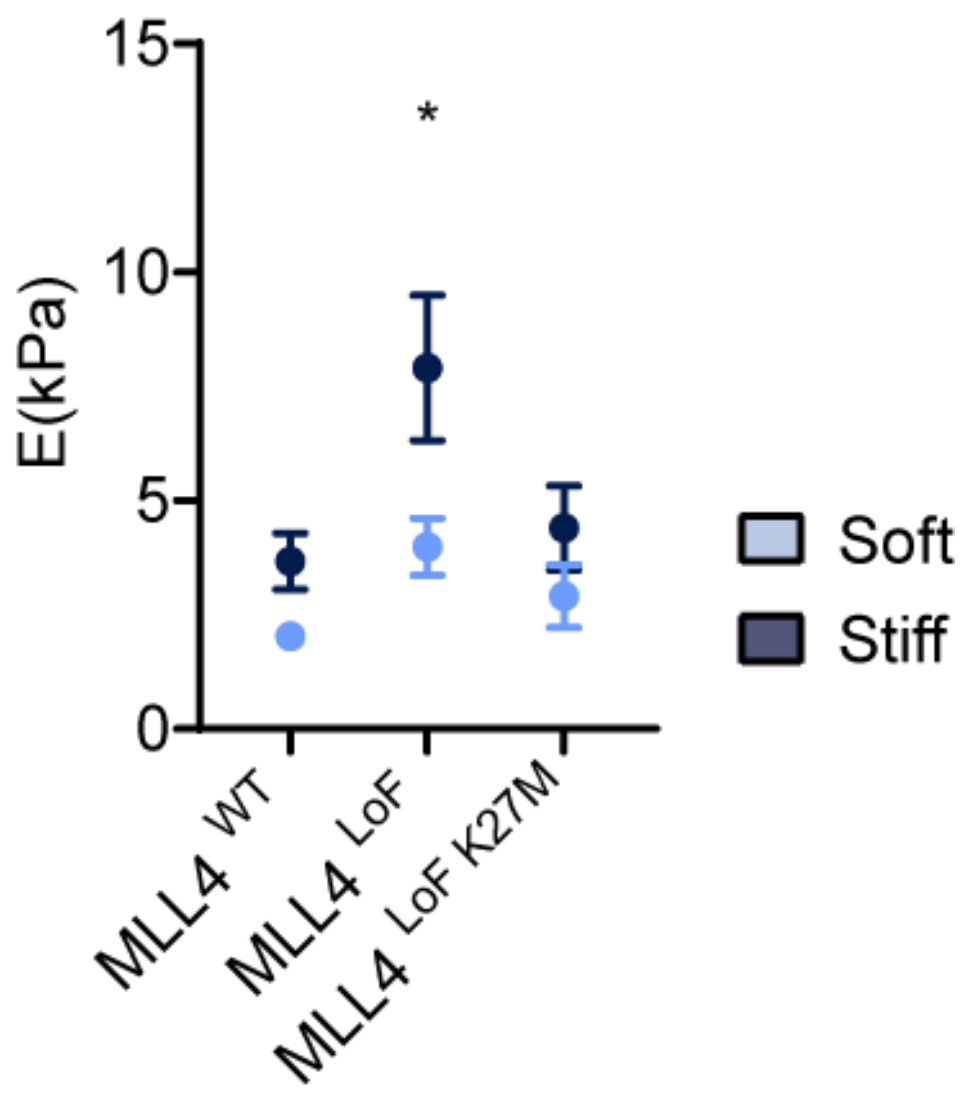

Scatter plot representing atomic force microscopy (AFM) measurements of nuclear stiffness. Data represent the elastic modulus of nuclei (E, kPa) in $MLL4^{WT}$, $MLL4^{LoF}$ and $MLL4^{LoF}$ K27M MSC in soft and stiff conditions. Each point corresponds to the Mean + SEM of at least 15 nuclei per condition from 2 different biological replicates. Statistical significance was determined by Sidak's multiple comparisons test (two-way ANOVA).

Appendix Figure S4. Analysis of Lamins in soft and stiff conditions.

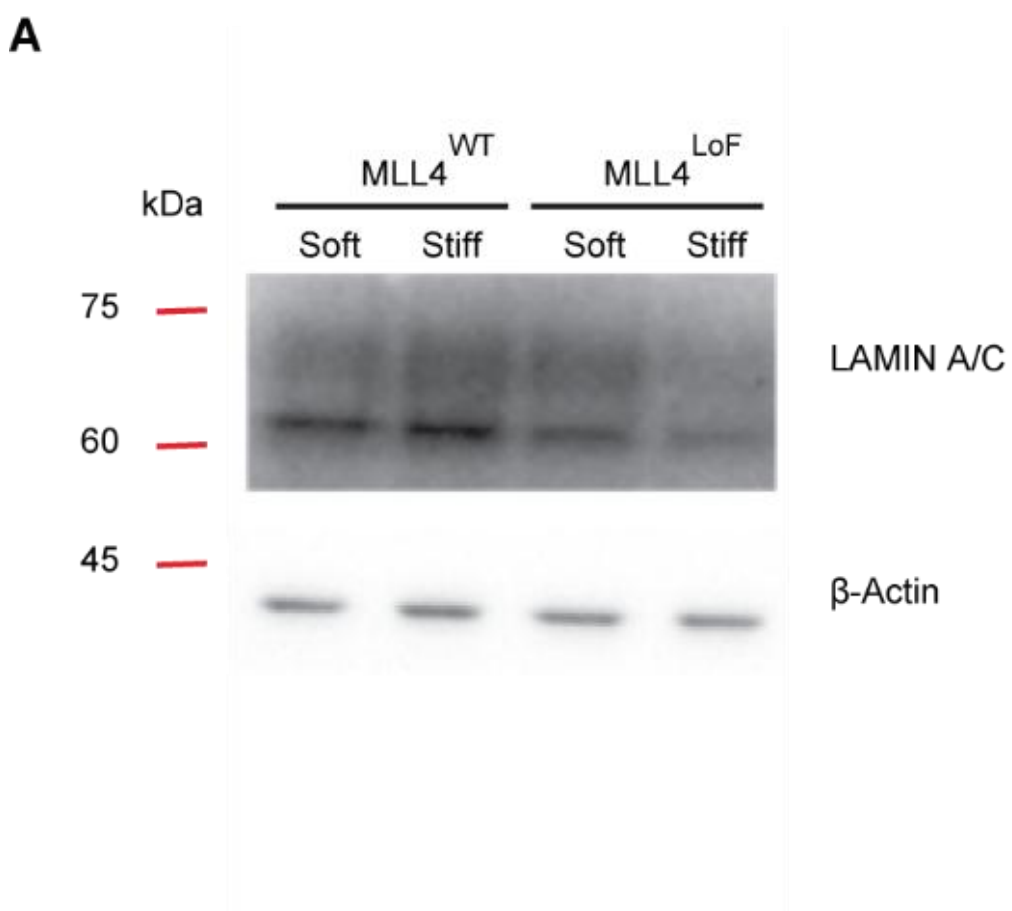

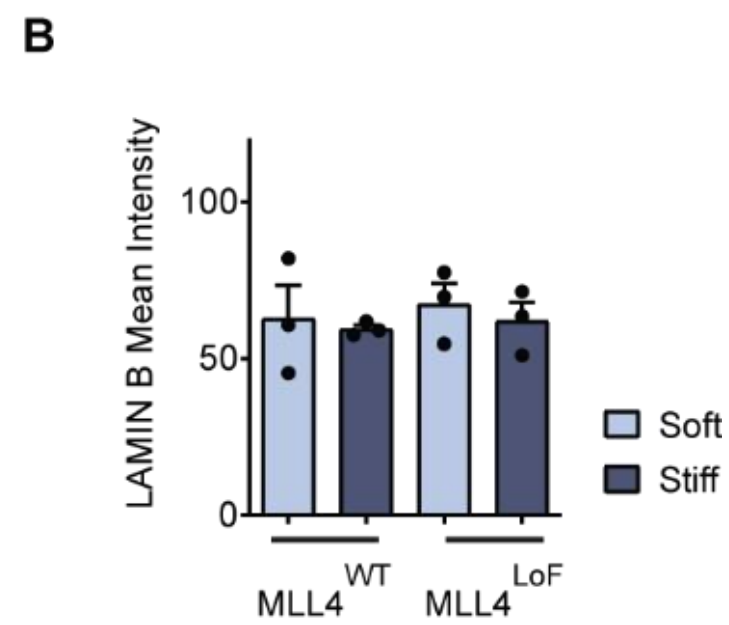

(A) Western Blot analysis of LAMIN A/C in $MLL4^{WT}$ and $MLL4^{LoF}$ MSCs plated in soft and stiff matrix. **(B)** Bar plots show Immunostaining quantification of nuclear LAMIN B Mean Intensity in $MLL4^{WT}$ and $MLL4^{LoF}$ MSCs plated in soft and stiff matrix (Mean + SEM of n=3 independent experiments). Statistical significance was determined by Sidak's multiple comparison test (two-way ANOVA).
