# Supplementary material for: Chromatin condensates tune nuclear mechanosensing and preserve nuclear integrity to prevent cGAS activation
Source: EMBO J. 2026 Aug 12;45(18):6384–420. doi: 10.1038/s44318-026-00884-z (PMC13584762; doi:10.1038/s44318-026-00884-z)
Supplement: Supplementary file 2 [file 44318_2026_884_MOESM2_ESM.pdf]

THE EMBO JOURNAL

Chromatin condensates tune nuclear mechanosensing and preserve nuclear integrity to prevent cGAS activation

Sarah D'Annunzio, Martina Di Santo, Giulia Vitali, Lucia Santomaso, Daniela Michelatti, Leonardo Morelli, Chiara Bernardis, Maulana Ariefai, Shiza Nasir, Sara Lago, Lorenzo Lunelli, Claudia Testi, Emanuele pontecorvo, Alessandro Poli, Fabrizio Pennacchio, Paolo Maiuri, Elodie Sanchez, David Genevieve, Lorenzo Petrolli, Thomas Tarenzi, Roberto Menichetti, Raffaello Potestio, Giancarlo Ruocco, and Alessio Zippo

Corresponding author(s): Alessio Zippo (alessio.zippo@unitn.it)

Review Timeline:

Submission Date:	8th Oct 25
Editorial Decision:	23rd Jan 26
Revision Received:	6th May 26
Editorial Decision:	25th Jun 26
Revision Received:	1st Jul 26
Accepted:	20th Jul 26

Editor: Cornelius Schneider

Transaction Report: This manuscript was submitted to The EMBO Journal after peer review at another journal.

(Note: With the exception of the correction of typographical or spelling errors that could be a source of ambiguity, letters and reports are not edited. Depending on transfer agreements, referee reports obtained elsewhere may or may not be included in this compilation. Referee reports are anonymous unless the Referee chooses to sign their reports.)

UNIVERSITÀ DI TRENTO

Department of Cellular, Computational and Integrative Biology - CIBIO

Università di Trento,
Dipartimento CIBIO
Via Sommarive 9, POVO

Trento, 7th of August 2025

For clarity, we have highlighted changes in the revised manuscript related to the requested information's as red text.

Reviewer comments:

Reviewer #1
This manuscript shows that MLL4 condensates can sense mechanical conditions of the cell and are important in protecting the nucleus from rupture and consequent activation of cGAS-STING pathway and cell death, and links MLL4 deficiency to the inflammatory response in Kabuki syndrome. This work is a significant extension of the authors' previous paper in Nature Genetics showing that MLL4 condensates regulates nuclear mechanical stress in Kabuki syndrome.
The mechanical perspective to study how nuclear condensates regulate cell function and impact pathologies is novel. The authors performed rigorous and well-controlled experiments, and the data are overall solid. While some of the major conclusions overlap with what their Nature Genetics paper has shown, this work has also added significantly more details. The part on nuclear envelop (NE) rupture and cGAS activation is new and important. While it is reasonable to believe that the primary activities of the nuclear proteins are to regulate the genetic function of the genome, their roles in the non-genetic function of genome is very interesting (and understudied). This work is a significant endeavor in the non-genetic function of these nuclear proteins. However, it would be much more insightful if they could partially address how much interplay of the (epi)genetic and non-genetic (mechanical) function of the MLL4 condensates is really mediating the whole process from sensing the mechanical environment to generating the cellular effects.
This work provide convincing evidence that MLL4, and especially its PrLD condensates can act as a cell/nuclear mechano-sensor. Considering that condensate formation in general is a physical phenomenon and inherently affected by mechanic forces, how unique or specific shall we view the MLL4-PrLD condensates (as opposed to other numerous nuclear condensates) as a mechano-sensor? Using the assays in Fig. 3., it would be very interesting to test condensates formed by other nuclear proteins (related and unrelated to MLL) and see if they respond differently to the different nuclear mechanic environment of MLL4 WT vs. MLL4(Q4092X) MSCs. These results may shed some light on the nature (pure mechanic or biochemical signaling) of mechano-sensing by these condensates. If the mechano-sensing property is shared by other nuclear protein condensates, this could broaden the significance of the work.

Response:
We sincerely thank Reviewer #1 for recognizing the novelty and significance of our study and for highlighting the importance of investigating non-genetic roles of chromatin regulators, particularly in nuclear mechanotransduction. We appreciate the Reviewer's suggestion to examine whether the mechano-sensing function we describe is unique to MLL4, or potentially shared by other nuclear proteins, both related or unrelated to MLL4.

While we agree that MLL4 is unlikely to be the only chromatin-associated factor with mechano-sensing properties, our data strongly support the conclusion that its prion-like domain (PrLD) plays a functionally specific role in mechano-sensing. To explore this, we first carried out structure prediction and molecular dynamics (MD) simulations focusing on the polyQ-rich stretch within the PrLD. Despite being part of an intrinsically disordered region, this segment showed a marked propensity to form transient α-helical conformations (Figure 4A–B). Importantly, under increasing simulated mechanical pressure, this α-helical content progressively transitioned into β-sheet-rich structures, as observed across distinct simulation trajectories (Figure 4C–F; S4A). This transition

mirrors what is known for mechanosensitive domains, which respond to stress by changing their secondary structure, a hallmark mechanism in protein-based mechano-sensing[24, 25].
To validate these predictions experimentally, we performed a targeted mutagenesis of the polyQ region. Based on prior evidence supporting the stabilizing role of interspersed Leucine residues in β-sheet formation[1], we substituted these residues with Alanine and expressed the resulting MLL4-PrLD variant in MSCs. This variant abolished mechano-induced condensate formation upon cell confinement (Figure 4G), closely phenocopying the effects of polyQ deletion (Figure 3I). Notably, the same mutant showed the same propensity to assemble into condensates in response to blue-light stimulations, strengthening the relevance of PrLD conformational transitions in MLL4 condensates formation (Figure S4B, C). Overall, these new set of experiments strengthen the relevance of PrLD conformational transitions as molecular mechanisms to decode the nuclear mechanical signals.
To test the broader relevance of this mechanism, we investigated whether other chromatin-associated proteins also exhibit similar mechano-response function. Specifically, we selected the transcriptional and Polycomb-associated proteins MED1 and BMI1 and fused them to the optogenetic module for controlling their nucleation by light stimulation. Both proteins successfully formed condensates upon blue-light stimulation (Figure S3H), confirming their capacity for LLPS. However, neither OptoMED1 nor OptoBMI1 formed condensates in response to mechanical stress (cell confinement; Figure S3H-I). These results suggest that mechanical responsiveness is not an intrinsic property of all chromatin condensates, at least not via the mechanism observed for MLL4.
These results do not preclude the possibility that other chromatin factors harboring different IDRs could harbor mechano-sensitive properties, possibly through other, yet to be defined mechanisms. Indeed, in two recent works it has been shown that mechanical load reshapes nuclear condensates, including the polarized distribution of BRD4 upon cell migration through constrictions[2, 3]. Overall, we acknowledge that it is currently challenging reaching a general conclusion regarding the mechano-sensory functions of chromatin factors belonging to condensates Accordingly, we have revised the Discussion section to reflect this broader context and to acknowledge that identifying molecular signatures that confer mechano-sensing properties to nuclear condensates remains a key challenge for the field.

Another major question is on how MLL4 LoF affects the downstream mechanical phenotypes of the cells, including NE more prone to rupture. The data do not really suggest whether this is a pure mechanical effects or involves transcriptional changes induced by MLL4 LoF. One could imagine MLL4 LoF causes changes in certain target genes that regulate NE resilience and integrity. Would be interesting and valuable to design assays to test these possibilities.

Response:
We are thankful to the Reviewer for this comment, which focuses on better dissecting the mechanisms by which MLL4 LoF affects NE integrity. In the original manuscript we began to address this point by analyzing the contribution of BAF1, which is a critical mediator of NE repair. We have found that BAF1 is downregulated in the MLL4 loss-of-function (LoF) conditions and contributed to the downstream activation of the cGAS/STING pathway (Figure 7A, Figure S7A-D). However, when we mimicked the ~2-fold reduction in BAF1 using CRISPR interference (CRISPRi), we were unable to fully recapitulate the extent of NE rupture and cGAS/STING activation observed in MLL4 LoF cells (Figure S7F–G). These findings suggest that while BAF1 contributes to the phenotype, additional pathways likely involving mechanosensitive signaling must be involved. In support of this, we previously showed that ATR hyperactivation contributes to MLL4 LoF-associated defects[8]. Recent studies[4, 5]have further established a link between ATR activity and Lamin A turnover, implicating ATR-mediated phosphorylation in NE instability. These findings are consistent with our data showing increased Lamin A/C dysregulation in MLL4-deficient cells.

Nevertheless, we further investigated this aspect by re-analyzing the gene expression profiling and found that signatures related to ECM, cell adhesion, and cytoskeleton contractility were downregulated in the MLL4 LoF condition, while we did not retrieve major alterations in the expression patterns of genes involved in NE homeostasis and repair (Figure S6D-E). We further corroborated the contribution of the cytoskeleton-transmitted mechanical forces acting on the NE by measuring the level of contractile perinuclear actin cap (Figure S6F). In addition, by interfering with the formation of a functional LINC complex through the overexpression of the dominant negative GFP–Nesprin-1/2–KASH protein, we showed that the NE rapture was depending on the proper balancing between chromatin- and cytoskeleton-mediated mechanical forces acting on the NE (Figure S6G).
We believe that the additional analyses and experimental validations helped in clarifying the integrated mechanisms by which MLL4 LoF causes the increased NE fragility. It remains to dissect whether the observed changes in gene

expression of ECM and cytoskeleton-related genes is resulting from a direct contribution of MLL4 to the chromatin states, or it is mediated by the action of downstream mechano-responsive TFs.

The work only studied cells with reduced MLL4 level, leaving an open question which regions and activities of MLL4 mediate the mechano phenotypes following mechano-sensing (mechano-sensing is likely mediated by PrLD, thought the assays were done in the presence of endogenous MLL4). Is the methylation activity and/or PrLD of MLL4 involved in turning the sensed mechanical changes to the cellular phenotypes? It is worthy of at least some discussion.

Response:
We agree that untangling the MLL4 mechano-response from its enzymatic activity is an important aspect related to this work. To experimentally dissect these contributions, one would ideally perform rescue experiments using either a methyltransferase-dead mutant, or a full-length MLL4 construct lacking the PrLD. However, such experiments are technically demanding because MLL4 is an exceptionally large protein (~5,537 amino acids), making it extremely difficult to express exogenously in a controlled and physiological manner. Overexpression of full-length MLL4, or its domain-deleted variants, often leads to non-physiological phenotypes due to protein mislocalization, aggregation, or over-saturation of interacting partners. We attempted to overcome this limitation by using CRISPR-based genome editing to introduce a Y5425A point mutation, which selectively inactivates the SET domain responsible for H3K4 methyltransferase activity. Unfortunately, despite extensive screening, we were unable to recover viable clones harboring this specific mutation, likely due to selective pressure against its integration. Given these technical limitations, we could not assess the potential contribution of MLL4 histone methyltransferase activity. Nevertheless, in the attempt to investigate the contribution of the PrLD in the MLL4 mechano-responsiveness, we found that the deletion of the PolyQ stretch from MLL4-PrLD abrogated its assembly into condensates upon cell confinement (Figure 3I of the revised manuscript). Furthermore, we showed that the impairment of PrLD conformation transition towards beta-like motifs by substituting the interspersed Leu into Ala impaired its assembly into condensates in response to cell confinement (Figure S3H, I). This new set of data supports a relevant contribution of the PrLD in mediating MLL4 responsiveness to mechanical cues. However, we can not exclude that other MLL4-associated functions including its methyltransferase activity could participate in mediating the investigated nuclear mechano-response. We have discussed this point in the Discussion section of the revised manuscript.

Fig. 1B, why were the BRD4 condensates (number and area) greatly increased in the MLL LoF cells compared to MLL WT cells (both in soft condition)?

Response:
We thank the Reviewer for this observation. Indeed, we consistently detected a relative increase in both the number and area of BRD4 condensates in MLL4 LoF cells compared to wild-type controls under soft matrix conditions. This finding was reproduced across independent mesenchymal stem cell (MSC) clones as well as in Kabuki syndrome (KS)-derived fibroblasts (Figure 1 A, B and Figure S1 E, F). To assess whether this increase reflects a broader phenomenon affecting transcriptional condensates, we extended our analysis to MED1, a coactivator similarly involved in phase-separated transcription hubs. Strikingly, MED1 condensates showed a comparable increase in number and size under MLL4 LoF and soft matrix conditions (Figure S1O), suggesting a generalized reorganization of transcriptional condensates in this context. Notably, we also detected an opposite trend (although not always statistically significant) for PcG-condensates, which showed a reduced number of condensates in the MLL4 LoF maintained on a soft matrix (Figure 1 C, D and Figure S1H-M).
We do not have a definitive mechanistic explanation for this phenomenon associated with the *KMT2D* haploinsufficiency and the weakening of external mechanical forces. We postulate that, under these conditions, cells may attempt to restore transcriptional robustness by enhancing the assembly or stability of transcriptional condensates (e.g., BRD4, MED1), possibly as a feedback mechanism to counteract chromatin softening.
Supporting this idea, both our previous work and recent publications indicate that MLL4 LoF does not cause a global transcriptional shutdown, despite its known role in enhancer regulation. This transcriptional buffering may be partly explained by an increase in active histone marks, such as H3K4me3[7]. In a parallel, in a yet to be published study, we also observed enhanced H3K4me3 deposition in MLL4-deficient MSCs, further suggesting that epigenetic compensatory mechanisms may help maintain transcriptional output. We cannot exclude the possibility that other chromatin regulators, not directly involved in histone methylation, also contribute to the maintenance or even potentiation of transcriptional condensates in this setting. These may include IDR-containing factors that assemble or stabilize condensates in response to changes in nuclear mechanics.

In summary, the increased BRD4 (and MED1) condensates likely reflect adaptive compensatory mechanisms triggered by the combined loss of MLL4 function and external mechanical signals. However, we recognize that fully disentangling the direct vs. indirect effects of MLL4 LoF will require integrated molecular profiling, ideally in combination with a degron-based acute depletion system, which would help temporally separate primary from secondary effects. That said, we believe that a full resolution of these mechanisms is beyond the immediate scope of this study and not essential to support our central conclusion: that MLL4-PrLD acts as a functional nuclear mechano-sensor, whose loss results in altered chromatin mechanics and nuclear integrity.

The authors used H3.3 K27M mutant to rescue the effects in many assays. This is good as it helps show the interplay of the mechanical and epigenetic interplay of the condensates in mediating the function. However, they always describe this as the result of rescuing the "PcG condensates" (implying the physical or even mechanical form of the PcG condensates may directly regulate the phenotypes), and, in Discussion, suggest that this shows "inward forces generated by chromatin compartmentalization are mechanically coupled with the outward mechanical stimuli". K27M may affect the phenotypes mainly through an epigenetic mechanism (inhibiting K27 methylation on target genes that may regulate mechanical phenotypes). Data here do not sufficiently distinguish the (epi)genetic mechanisms from pure mechanical mechanisms.

Response:
This is an important point raised by the Reviewer, and we agree that our current approach cannot fully distinguish between the effects on PcG enzymatic activity and chromatin compartmentalization, as noted in the original manuscript. Unfortunately, there are no available methods that can effectively separate the (epi)genetic from the mechanical functions, and it is likely that these mechanisms are interrelated, further complicating the analysis.
Nevertheless, we agree to emphasize in the discussion section of the revised manuscript that our strategy cannot uniquely distinguish between these two mechanisms, which represents a limitation of the study.

We appreciate the reviewer's insightful comment and fully agree that the H3.3 K27M mutant, while instrumental in our assays, does not allow us to completely disentangle the contribution of PcG enzymatic activity from the biophysical and mechanical roles of PcG condensates. The interpretation that the K27M-mediated phenotypic rescue arises from altering PcG condensates does indeed rest on the assumption that condensate organization and epigenetic regulation are functionally intertwined.

We recognize that the current state of the field lacks experimental tools that can clearly dissect these two layers of function (biophysical, condensate-mediated compartmentalization versus enzymatic histone mark deposition) in a fully independent manner. Moreover, our data suggest that these mechanisms may not operate in isolation but rather converge to regulate nuclear architecture and mechanotransduction in a cooperative manner. Therefore, the effects we observed likely reflect a combination of both altered chromatin epigenetic states and changes in condensate behavior that collectively influence nuclear mechanics.
In response to this comment, we have revised the Discussion to explicitly clarify this limitation. We now emphasize that while the H3.3 K27M mutant is a powerful tool to probe the involvement of PcG-associated chromatin domains, our findings do not unambiguously distinguish between the epigenetic and mechanical effects of PcG condensates. We believe this clarification improves the interpretive balance of our manuscript and avoids overstating the mechanistic specificity of the K27M rescue experiments.

Reviewer #2
The manuscript from Alessio zippo et al. explored the role of chromatin condensates in nuclear mechanosensitivity and response, especially the function of MLL4 in Kabuki syndrome. This study is of value to the research field and offers potential therapeutic benefit, but there are some concerns of the manuscript that should be resolved prior to publication. I have detailed suggestions below:

Response:
We are pleased that the Reviewer#2 appreciated the added value of this work in which we described and characterized the role of chromatin condensates in mediating the nuclear mechano-response.

Major points:
1. In Figure 1-S1, the study concluded that external mechanical forces is associated with an augmented MLL4-dependent assembly of transcriptional condensates. Can authors explain why MLL4 LoF. MSCs exhibited

significantly contrary tendency, such as more BRD4 condensates, less condensates compromising PRC1 in soft group?

Response:
As correctly noticed, we found that in the soft condition there is a diminished clustering of PcG condensates, with respect to the stiff condition (Figure 1 C, D and Figure S1H-M). This pattern is counterbalanced by the increased of the transcriptional-associated condensates, as detected by monitoring the clustering of BRD4 (Figure 1 A, B and Figure S1 E, F), and further confirmed through the detection of MED1 distribution (Figure S1O) Given the reproducibility, we can postulate that the measured increased of transcriptional condensates in the MLL4 LoF MSCs grown with a low mechanical load (soft conditions), represent a mechano-related response, which permits to preserve the nuclear mechanical properties by counterbalancing the PcG condensates.
From a broader chromatin context, we can anticipate that possible compensatory mechanisms may modulate transcriptional condensates under conditions of reduced mechanical input and MLL4 loss-of-function (LoF).
Indeed, both our previous work[8] and emerging studies indicate that MLL4 LoF does not lead to widespread transcriptional collapse. This can, in part, be attributed to compensatory increases in active histone marks, such as H3K4me3[7]. In an independent, ongoing project, we have further observed enhanced H3K4me3 deposition in MLL4-deficient MSCs, supporting the idea that cells may engage redundant or alternative chromatin-based mechanisms to preserve transcriptional capacity. While these findings raise the possibility that other chromatin factors may contribute to the observed resilience of transcriptional condensates, these mechanisms remain speculative at this stage. Notably, the potential involvement of non-histone-modifying factors in reorganizing condensates under altered mechanical conditions cannot be ruled out. We agree that comprehensive molecular profiling, ideally in combination with our MLL4-specific degron system, will be necessary to dissect direct versus indirect effects of MLL4 loss on chromatin organization and condensate dynamics. However, we respectfully note that such an in-depth mechanistic investigation is beyond the current scope of this work. Importantly, we wish to clarify that the central conclusions of our study indicating that MLL4 condensates serve as mechanosensitive chromatin hubs are based on direct functional and biophysical evidence, including nuclear mechanical measurements and condensate behavior. While the identity and contributions of potential compensatory chromatin regulators remain to be fully elucidated, these findings do not alter the core novelty and significance of our discovery that MLL4 plays a role in nuclear mechanotransduction.

Also, can the author explain why these two stiffness values (soft (0.5kPA) and stiff (32kPA)) were chosen for the experiment. Also, authors have utilized fibroblasts from healthy donor (Ctrl) or Kabuki patients (Mut) in their previous publication and current investigation, can authors obtained mesenchymal stem cell from donor and patients for investigation.

Response:
In physiological context, cells encounter a wide range of extracellular matrix (ECM) elasticities across different tissues[6]. By mimicking these varying stiffnesses in vitro, studies have shown that mesenchymal stem cells (MSCs) differentiate optimally into specific cell types-such as adipocytes, chondrocytes, or osteoblasts-depending on the substrate's elasticity, which mirrors the physiological ECM stiffness of their respective tissues[6, 7]. Based on this rationale, we selected substrate stiffness values corresponding to the elastic moduli of soft (adipose) and stiff (cartilage) tissues, corresponding to 0.5kPa and 32kPa, respectively.
Concerning the possibility to retrieve MSCs from patient-derived primary fibroblasts, this may represent an interesting suggestion to further demonstrate that the observed mechano-related phenotype is independent from the genetic mutations determining MLL4 LoF. However, trans-differentiation procedures are often inefficient and variable, causing a certain level of heterogeneity in the retrieved cell population. We thus adopted an alternative strategy to control MLL4 abundance, independently for genetic mutations of *KMT2D*, namely developing an endogenous MLL4 degron system in MSCs (as detailed in the next response to the same Reviewer). The obtained results confirmed that MLL4 abundance determines the balancing of chromatin condensates (Figure S7T, U), which impinge on NE integrity (Figure S7V), resulting in aberrant activation of cGAS-STING pathway (Figure 7J, K).
Besides these new sets of data that confirmed our previous findings, we would like to stress that in the original paper we confirmed the major results also using an independent MSCs mutant carrying a different mutation of *KMT2D*, further supporting the raised conclusions.

2. A recent study has reported that KMT2D truncation mutations lead to cytosolic accumulation of truncated MLL4, and correction of KMT2D truncation mutation in an isogenic background restores multifaceted MLL4 functions

(PMID: 38113256). Can authors further explore the roles of nuclear to cytoplasmic translocation of MLL4 in regulating mechanosensitivity and response, and whether correction of KMT2D truncation mutation can reverse the mechanosensitivity and response.

Response:

In our previous work, we demonstrated that the truncating mutations introduced in the engineered MSC models result in a loss of MLL4 function (LoF), primarily via nonsense-mediated mRNA decay (NMD). This led to reduced mRNA levels, decreased MLL4 protein abundance, and diminished deposition of the H3K4me1 histone mark[8]. These findings were validated through immunofluorescence (IF), which did not reveal any aberrant cytoplasmic redistribution of MLL4, neither in MSCs nor in primary fibroblasts derived from Kabuki Syndrome (KS) patients[8]. To further address this point, we extended our analysis by performing IF using an antibody targeting an epitope located at the N-terminus of the MLL4 protein, thus detecting potential truncated forms. Our data confirm that, at least in the KS disease model we developed, truncating mutations do not lead to cytoplasmic accumulation of MLL4 (Figure S1A–C). Instead, the mutant protein undergoes degradation, as quantitatively demonstrated by both IF (Figure S1B, C)[8]. Therefore, we are confident in excluding the possibility that the observed mechano-related phenotypes are due to cytoplasmic mislocalization of MLL4.

Importantly, we do not consider our findings to be in contradiction with the referenced study[9]. In oncogenic settings, alterations in signaling pathways and disruptions in NMD regulation could plausibly permit the accumulation of truncated MLL4 in the cytoplasm, a mechanism that appears absent in the context of non-transformed MSCs or primary fibroblasts derived from KS patients.

To directly evaluate whether MLL4 function modulates the mechano-response, we developed a PROTAC-degron-based strategy. Specifically, we knocked in a HaloTag module at the 5' end of one KMT2D allele, generating an endogenous HaloTag-MLL4 fusion protein. This construct enables temporal control of MLL4 protein degradation via a PROTAC-guided system (Figure 7H, I and Figure S7O–Q).

We first confirmed that this system could faithfully mimic KMT2D haploinsufficiency by demonstrating a ~50% reduction in MLL4 protein levels within 8 hours of PROTAC treatment. This degradation was accompanied by decreased H3K4me1 and, to a lesser extent, H3K27Ac levels, without affecting cell viability or proliferation (Figure S7P–S). Notably, the acute degradation of MLL4 disrupted the balance between transcriptional (e.g., BRD4) and PcG-associated condensates, confirming its role in chromatin compartmentalization (Figure S7T–U). Regarding the mechano-response, MLL4 depletion decreased Lamin A/C expression (Figure S7V), and an increased frequency of nuclear envelope (NE) rupture, as assessed by cGAS/STING foci formation (Figure 7J). Finally, under mechanical confinement, HaloTag-MLL4 degradation phenocopied the mechanical hypersensitivity observed in KMT2D haploinsufficient cells. This was evident through increased cGAS/STING pathway activation and subsequent programmed cell death (Figure 7K).

Collectively, these new results provide direct evidence that MLL4 levels critically regulate nuclear mechanical properties, chromatin condensate equilibrium, and mechano-responsiveness in MSCs. Our findings underscore that MLL4 abundance defines the cellular mechano-response, establishing MLL4 as a key nuclear mechanosensor. This is consistent with previous studies showing that KMT2D is a dosage-sensitive gene, where both haploinsufficiency and triplosensitivity result in phenotypic abnormalities[10]. Dosage-sensitive genes are frequently enriched for intrinsically disordered regions (IDRs) that promote multivalent, weak interactions critical for phase separation and condensate dynamics[11, 12]. By selectively targeting MLL4's capacity to form condensates and by demonstrating that its abundance dictates mechanical responsiveness, our study provides evidence that KMT2D dosage sensitivity is mechanistically linked to the PrLD-dependent biophysical properties of MLL4 condensates.

3. Multiple lines of evidence have indicated that the shape of the nucleus and NE integrity are mediated by a dome-like structuring of F-actin filaments, called the perinuclear actin cap, which wraps around the apical surface of the nucleus, keeping the NE smoothly distended and preventing its deformation. Actin cap filaments directly connect to the NE, in part through the LINC complexes, forming a structural continuum between the cytoskeleton and the NE. Authors should illustrate the connections between cytoskeleton and the NE, as well as the potential roles of cytoskeleton-related factors in regulating chromatin condensates-mediated nuclear mechano-sensing and integrity.

Response:

We thank the Reviewer for highlighting the crucial role of the perinuclear actin cap and LINC complex in regulating nuclear shape and integrity. To directly examine the functional coupling between cytoskeletal forces and nuclear mechano-responsiveness, we designed a series of experiments to decouple nuclear mechanics from cytoskeleton-

transmitted external forces. Specifically, we overexpressed a dominant-negative GFP–Nesprin-1/2–KASH construct, which disrupts the formation of a functional LINC complex and thus blocks mechanical signal transduction from the actin cytoskeleton to the nuclear envelope. This experimental strategy allowed us to interrogate the role of cytoskeleton–NE coupling in modulating chromatin condensate behavior and nuclear mechanical homeostasis. Strikingly, upon LINC complex disruption, the mechanically induced increase in transcriptional condensates typically observed in response to high ECM stiffness was abrogated, phenocopying the behavior of MLL4 LoF cells (Figure 1A–B and Figure S1G). Moreover, the expected reduction of PcG-associated condensates under high stiffness conditions was rescued by impairing LINC complex activity (Figure 1C–D and Figure S1L), suggesting that external force transmission through the cytoskeleton is required for proper chromatin condensate compartmentalization. Importantly, under these same conditions, we also observed a reduction in nuclear envelope (NE) integrity, recapitulating the phenotype seen in MLL4-deficient cells (Figure S6G). These observations were further supported by independent evidence from the Nesprin1-based FRET sensor, which confirmed reduced cytoskeletal tension transmitted to the NE in MLL4 LoF conditions (Figure 5F). Additionally, transcriptomic analyses revealed a downregulation of genes involved in cytoskeletal organization, focal adhesion, and ECM remodeling in MLL4 LoF cells (Figure S6C–F). These changes likely compromise actomyosin contractility, weakening the overall cytoskeletal architecture and further contributing to impaired nuclear force balance.
Collectively, these findings demonstrate that mechanical communication between the actin cytoskeleton and the nucleus, mediated through the LINC complex, is essential for proper chromatin condensate reorganization and nuclear mechanosensing. They support the central hypothesis that the balance between outward (cytoskeleton-derived) and inward (chromatin-based) forces is fundamental for preserving nuclear envelope architecture and mechanical resilience. Moreover, they suggest that MLL4 functions at the interface of this force equilibrium by influencing the composition and dynamics of nuclear condensates in response to mechanical cues.

4. The previous study of the authors has shown that MLL4 LoF was associated with reduced nuclear YAP/TAZ in MSCs, and previous studies have reported YAP add-back can rescue distorted nuclear shape, and inhibit cgas-sting activation. Authors should investigate the roles of YAP/TAZ in chromatin condensates-mediated nuclear mechano-sensing, and whether restoring YAP/TAZ activity can protect NE integrity and inhibit cgas-sting activation in MLL4 LoF MSCs.

Response:
We thank Reviewer #2 for highlighting the potential contribution of YAP/TAZ to chromatin condensates-mediated nuclear mechano-sensing, nuclear shape, and NE integrity in the context of KS. To address this point, we took advantage of the already established Tet-On inducible system to restore YAP/TAZ levels in MLL4 LoF condition[8]. We found that by restoring YAP/TAZ nuclear abundance, we rescued nuclear shape, NE integrity, and cGAS clustering, resulting in a diminished frequency of cell death upon mechanical stress (shown in Figure S5L, M; Figures S6C; Figure S7N). These findings suggest that in the KS condition the reduced NE tension limits the YAP/TAZ nuclear shuttling[13], thereby exacerbating the occurrence of dysmorphic nuclei and NE raptures, activating cGAS/STING signaling and the mediated cell death. By including these new set of data, we better clarified the mechanism by which the MLL4-dependent perturbation of chromatin condensates affects the balancing between inwards and outwards nuclear mechanical forces, resulting in a diminished activity of YAP/TAZ in controlling nuclear shape and NE integrity.

5. Authors should investigate whether Remodelin, a small molecule that has been shown to rescue nuclear shape, can prevent the cGAS/STING-mediated cell death in KS.

Response:
We thank Reviewer #2 for the insightful suggestion to investigate whether Remodelin, a small molecule previously shown to ameliorate nuclear shape abnormalities by targeting the nucleolar acetyltransferase NAT10, could also rescue nuclear envelope (NE) integrity and suppress cGAS/STING-mediated cell death in MLL4 LoF mesenchymal stem cells (MSCs), thereby further supporting the mechanistic link between NE deformation and inflammatory signaling in Kabuki Syndrome (KS). In response, we treated MLL4 LoF MSCs with Remodelin at concentrations ranging from 10 μM to 40 μM, based on previously reported efficacious doses. However, our results indicated a poor responsiveness to the treatment. Specifically, Remodelin did not rescue the increased NE fragility, as evidenced by persistent NE rupture events and no significant reduction in cGAS clustering or downstream cell death under mechanical stress conditions (see Supplementary Figure 1).

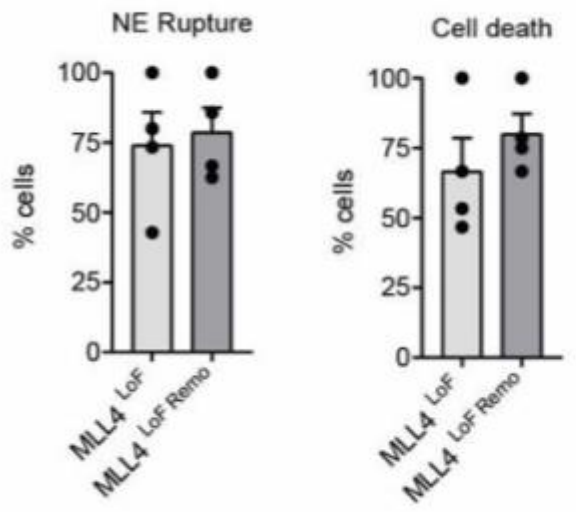

Quantification of NE rupture and cell death of MLL4LoF MSCs untreated or treated with Remodelin (40uM) and passing through microchannels of 3um.
Bar plots show Mean + SEM of n=3 independent biological replicates.
Statistical significance was determined by unpaired student's t-test.

Supplementary Figure_1

Although disappointing, these results were not fully unexpected, given that Remodeling inhibits the nucleolar Acetyltransferase NAT10, whose transcript level is unaltered in the MLL4 LoF MSCs. Although we tested different concentrations of Remodeling (from 10 to 40 μM), we can not exclude that a different treatment regime could partially rescue the observed mechano-induced phenotype. In addition, our data showed that the major drivers of the altered nuclear mechanics in the KS condition is related to the balancing of chromatin condensates, possibly involving multiple layers of chromatin modifications and genome organization, which could not be restored by targeting a single chromatin factor. Overall, we are confident that the poor responsiveness to Remodeling does not impinge on the robustness of our findings yet underlying the possibility to screen for small molecules rescuing the defective nuclear mechanics in the context of KS.

6. In vivo studies should be conducted to investigate the protective effects of STINGi (H-151 HY- 112693), or Remodelin, restoration of YAP-TAZ activity in KS, and cGAS-STING inactivation-related mice, YAP-overexpression mice can be adopted for in vivo investigation.

Response:
While we acknowledge the therapeutic potential of our findings, we believe that testing these drugs and/or genetic backgrounds in a preclinical mouse model of KS is beyond the scope of this study. Evaluating the efficacy of these compounds in addressing clinical manifestations associated with KS would require the establishment of multiple pre-clinical mouse models and testing different therapeutic regimes to determine appropriate dosages and tissue-specific responses. While we fully acknowledge the potential of chromatin-mechanical models to inform therapeutic directions, including STING or YAP/TAZ targeting, we believe these translational applications constitute a distinct follow-up study. As such, they fall outside the mechanistic and conceptual focus of the present work, which primarily dissects the cell-intrinsic roles of MLL4 in chromatin condensate dynamics and nuclear mechano-responsiveness.

7. MLL4 is one of the most frequently mutated genes in human malignancies, particularly epithelial cancers, so authors can also investigate the roles of MLL4 in mediating mechanosensing of epithelial cells, not only mesenchymal stem cells.

Response:
We thank the Reviewer for highlighting the potential broad implications of our findings and the possibility of extending the relationship between MLL4 LoF and the pathogenesis of epithelial cancers (carcinomas). However, there are some important aspects to be considered:

- the mechanobiology of epithelial and mesenchymal cells differs substantially, considering the diversity in cell adhesion (cadherin-dependent vs ECM-mediated), cytoskeleton organization (cortical actin vs stress fibers and actin apical cap), and nuclear mechanics (different chromatin context). Specifically, in mesenchymal cells, the nucleus contributes to the cell mechano-response, with cytoskeleton-transmitted forces acting directly on the NE through the LINC complex. In this respect, the applied outward forces are balanced by chromatin-mediated inward forces to preserve nuclear morphology and genome integrity. Indeed, the elongated nuclear morphology and its flexibility represent a hallmark of mesenchymal cells, which are capable of reshaping the nuclei in response to mechanical loads occurring, for example, during

cell migration or confinement. On the contrary, epithelial cells are organized within tissues to define physical barriers, subject to tensional forces dissipated and transmitted through the cell adherent junctions. These key differences are also reflected by the divergent mechanosensitive gene programs, which are activated by the mechanoresponsive TFs and cofactors such as YAP/TAZ, MRTF-SRF, β-catenin, and AP-1 family members. The implications of these differences in mechanobiology are well exemplified by the recent findings showing a specific contribution of YAP/TAZ-mediated mechanotransduction to stromal (mesenchymal) cells ageing, which depends on the YAP/TAZ-dependent preservation of nuclear shape and NE integrity, thus preventing the activation of cGAS/STING signaling and senescence[14]. Finally, the differences in the relative Lamin A/C abundance and the modalities through which chromatin modifications and organization contribute to the mechanical response can explain the diverse contribution of nuclear mechanics in epithelial and mesenchymal cells [8, 15, 16].

- Individuals affected by KS experience various clinical manifestations, including skeletal muscle hypotonia, cardiac and kidney defects, altered immune responses, and developmental-associated intellectual disabilities. All these symptoms may be linked to defects in activating appropriate mechano-responses in the stromal cells. However, current evidence suggests that epithelial cell functions and mechano-responsiveness in KS individuals remain largely unaffected, as they do not exhibit major clinical manifestations related to dysfunctional epithelial cells and tissue barriers.

Therefore, we postulated that MLL4 may contribute differently to mechanobiology, depending on the cell-specific nuclear mechanical properties and the cytoskeleton-nucleus coupling mechanisms. For these reasons, we focused only on the MLL4-related mechanobiology of mesenchymal cells in vitro and in vivo.

Minor points:

1. Introduction has to better clarify the scientific questions of this study and provide more theoretical background. Authors have to better describe the interaction of chromatin structure, MLL4, nuclear envelope rupture; nuclear integrity, and mechano-sensing under the background of Kabuki syndrome.

Response:
We revised the introduction section by providing a more comprehensive background on Kabuki syndrome and the role of nuclear mechanobiology in the associated pathological manifestations, as requested. Additionally, we also revised the text to better specify the biological questions being addressed and to offer greater insights into the background supporting the hypothesis.

2. The result part "MLL4 condensates are responsive to external mechanical forces" is difficult to read and understand, please revise this part by clearly presenting major findings and conclusions.

Response:
We revised the text accordingly to improve the clarity and the readiness of this section.

3. Some grammatical mistakes should be corrected, such as "external mechanical forces is associated with an……" is should be corrected to are. "……. in line with what we observed in vitro (Figure 1G).", I do not find figure 1g in your manuscript. Please check and revise your manuscript carefully.

Response:
We apologize for the grammatical errors and/or typos. We revised the text, as requested.

Reviewer #3

Annunzio and colleagues present a study entitled "Chromatin condensates tune the nuclear mechano-sensing and integrity, preventing cGAS activation" which aims to show that relative amount of transcriptional versus Polycomb condensates tunes nuclear mechano-responsiveness to extracellular matrix stiffness using MLL4 loss-of-function in Kabuki Syndrome in mesenchymal stem cells as a model. Specifically, the authors propose that MLL4 increased transcriptional chromatin condensates which soften chromatin and that this mechanism is important for preserving nuclear integrity during confined cell migration as absence of MLL4 in this context lead to cGAS-STING activation and subsequent cell death.

While initially excited by the concept, despite the limited context of Kabuki syndrome and LoF of MLL4, the authors actually already published (PMID: 33169020) and extremely similar story showing that MLL4 in the same Kabuki

syndrome system impairs Polycomb-dependent chromatin compartmentalization. There they implicate ATR in the process.

Response:
We thank the Reviewer for raising this important point, and we are sorry that we did not adequately deliver the differences in the addressed biological questions. Our previous study investigated how dysfunction of MLL4 contributes to the etiology and progression of Kabuki Syndrome and, more generally, how it determines the proper chromatin environment to modulate the genome functions8. We found that MLL4 participates in assembling transcriptional condensates through its PrLD, counterbalancing the Polycomb-dependent chromatin condensates, contributing to determining the nuclear mechanical properties. Furthermore, we showed that upon the MLL4 LoF, the nuclear mechano-sensor ATR resulted chronically activated, and its inhibition rescued the MLL4-dependent mechanical stress, permitting a proper commitment of mesenchymal stem cells towards chondrocytes, both in vitro and in vivo.

The present work addresses a fundamentally different biological question: how chromatin senses external mechanical forces and decodes this information to maintain cellular homeostasis and function. Specifically, we investigated the role of chromatin condensates in determining the mechanical properties of the nucleus.

By integrating various methodologies to mimic the different types of mechanical stresses that cells may experience within tissues, we found that MLL4 functions as a mechano-sensor, forming condensates in response to increased mechanical load. Additionally, we demonstrated that nuclear integrity relies on the balance between transcriptional condensates and PcG condensates. When this equilibrium is disrupted, as in the case of KS characterized by MLL4 LoF, the nuclei exhibit increased nuclear envelope rupture and subsequent activation of the cGAS/STING signaling pathway in response to mechanical stress.
All these findings are novel and were not addressed or anticipated in our previous work or in any other published articles. For these reasons, we believe this work contributes significantly to defining the role of chromatin condensates beyond their potential functions in gene expression and DNA damage response, as acknowledged by Reviewers #1 and #2. Furthermore, our findings open new perspectives in the field of mechanobiology by highlighting the importance of chromatin organization in determining nuclear mechanical properties and the mechanical balance in response to external forces. These findings go beyond the current stage of understanding the role of the nucleus in mechanobiology, which has been considered limited to the involvement of the nuclear lamina and the interconnection with the cytoskeleton through the LINC complex.
We have now improved the Introduction and Discussion sections to better clarify the addressed biological question and the relevance of our findings, showing how chromatin condensates contribute directly to the nuclear mechano-responsiveness.

My enthusiasm was further dampened by the extremely poor an inappropriate statistical analyses and poor quality of images, especially as it compared to the previously published study. From the onset in figure 1, I do not see the phenotypes in the provided images or any statistical significance in the presented graphs (authors use cell numbers to do statistics until figure 6 until when they do start pooling means of replicas togethers, however even then they take >2 condition dataset and break it down into pairs and do paired statistical analyses, which are again not proper).
The study is too repetitive with their previous work and improper statistical analyses make it impossible to review this manuscript. There are also some typos and mislabeled figures, some of which I point out.

Response:
We are sorry that the Reviewer enthusiasm for our work may have been diminished due to concerns regarding the applied statistical methods. Specifically, the Reviewer criticized our choice of using "n", the number of clusters (or their intensity)/per cell, which were obtained from three independent biological replicates. We originally selected this method because it accurately represents the distribution of our data, which is intrinsically heterogeneous.
Nonetheless, we acknowledge that the chosen statistical methods bring about limitations regarding the possibility of determining the statistical significance of differences measured among the analyzed conditions. To address this point, we conducted a Tukey's and Sidak's multiple comparison test (two-way ANOVA) evaluating the differences between the means of the multiple groups, considering the analyzed conditions as dependent events (genotypes and mechanical loads). In addition, to strengthen the robustness of our results, we increased the numerosity of the analyzed conditions to reach a numerosity of biological replicates n>3 in most of the assays. Of importance, by

reanalyzing our data, we confirmed that clustering of transcriptional and PcG condensates increased and decreased in response to the augmented matrix stiffness, respectively (Figure 1A-D, Figure S1A, S1D-0). In addition, we confirmed that MLL4 abundance determines a time-dependent post-natal growth delay, which is associated with a nuclear mechanical response to tissue stiffening, in vivo (Figure 2A, B and Figure S2A, B). Similarly, we confirmed that the increase of mechanical stress (either by ECM stiffness or cell confinement) supported the dynamic clustering of MLL4 PrLD (Figure 3C, 3D, 3F, and Figure S3D), which resulted in being dependent on the conformational flexibility of the PolyQ stretch (Figure 4G). Concerning the contribution of MLL4 clustering to the nuclear shape, we verified the increase in Lamin A/C abundance in response to matrix rigidity, which was abrogated upon MLL4 LoF, though the weakening of the cytoskeleton-transmitted mechanical forces in the NE (Figure 5A, 5F, Figures S5A-C and Figure S5L). To assess the effects of genotype, stiffness, and their interaction on Area, Excess of Perimeter, Flatness, Volume, and nuclear biophysical properties, we employed linear mixed-effects models. Here, genotype and stiffness were treated as fixed effects, while replicates were considered as a random intercept to account for repeated measurements. Model fitting was performed using Restricted Maximum Likelihood (REML). We report the p-values associated with interaction terms (genotype × stiffness) to identify context-specific genotype effects across stiffness conditions. This model is well-suited to analyze morphometric features (Area, Excess of Perimeter, Flatness, Volume, and material properties) because it accounts for both fixed effects (genotype and stiffness) and random variability across replicates. By including replicates as a random intercept, it captures technical variation and improves the reliability of effect estimates, allowing us to detect true genotype × stiffness interactions. In addition, the Linear mixed model is not limited by the dynamic range of measurements (detected by Brillouin and EOP), thus, even with narrow ranges, it helps isolate subtle effects while controlling for replicate-level noise. By determining statistical significance with a linear mixed-effects model, we verified that the MLL4 LoF weakened the nuclear mechano-response in response to the augmented ECM rigidity, in relation to nuclear shape (area, volume and flatness), NE invagination, and chromatin stiffening (Figure 5C-E, Figure S5D-H, Figure S5 K, M, and Figure 1F). Notably, the altered, MLL4-dependent nuclear mechanical response was restored by rescuing the PcG clustering through the expression of the H3.3K27M or by restoring the YAP/TAZ nuclear abundance. Finally, we also confirmed the statistical significance of the increase of NE rapture, which induced the increment of cGAS clustering into foci, triggering the activation of STING pathway, resulting in an augmented cell death, in response to mechanical stress (Figure 6A, 6G, 6I, Figure S6A, S6B, S6G, S6J, S6L, Figure 7B, 7C and 7F).
In sum, we are convinced that by increasing the numerosity of biological replicates and improving the statistical analyses, we strengthen the significance and robustness of our data. We thank the Reviewer for this constructive comment that ultimately helped us improve the relevance of our results to better support the raised conclusions.

Conceptually, the way that the manuscript is written requires a lot of external knowledge on the Kabuki syndrome and MLL4 - it is not all well introduced and motivated for a general audience. The previously published results on this same topic should have been discussed at length to motivate this current continuation work - of course I understand why as many findings were already shown there but this would make the work much more assessable and pleasant to read and may be more understandable from the onset. Furthermore - why is substate stiffness modulation, in particular, relevant to Kabuki Syndrome? This manipulation, which is central to the work, comes out of the blue - it is, again, completely unmotivated.

Response:
We have introduced the topic related to Kabuki Syndrome and MLL4 in the Introduction section, as requested. Specifically, we described the previous findings and clarified the potential link between the alterations in the mechano-response and the clinical features characterizing KS-affected individuals, thus emphasizing the rationale and clinical relevance of this work (see also the Discussion section of the revised manuscript). Indeed, many of the clinical manifestations can be associated with a perturbed mechano-response as exemplified by the postnatal growth retardation, skeletal muscle hypotonia, facial dysmorphisms, delayed immune response, and cardiac and kidney defects. All the mentioned manifestations involve tissues whose functioning and homeostasis rely on mechano-signaling, thus representing a rationale to interrogate the contribution of the nuclear mechanics to the improper mechano-response in KS. We are confident that the revised version of the manuscript properly delivers the rationale of the study and the novelty of our findings, including their potential impact on other pathological contexts.

Some specific comments

1. The fact that transcription levels remain unaltered in all the manipulations (Figure S1C) is conceptually puzzling - if there really was differential condensation, why would there be no difference in transcriptional activity?

Response:
We thank the Reviewer for this comment, which helps us better understand the importance of the biophysical context for tuning the dynamic formation of chromatin condensates. Generally speaking, the assembly of chromatin condensates is guided by the physical mechanism of liquid-liquid phase separation (LLPS). At equilibrium, LLPS occurs when the concentration of macromolecules increases beyond a certain threshold, thus suggesting that any mechanisms changing the protein abundance, including transcription regulation, can lead to the formation or dissolution of condensates. Based on this concept, it would be expected that the mechanical load should change the protein abundance, possibly by altering the transcript levels of genes codifying for the chromatin factors contributing to the assembly of the analyzed chromatin condensates. However, the nucleus represents a crowded environment in which many dynamic processes, including transcription, RNA splicing, and genome doubling, are co-occurring, thus increasing the viscosity of the nucleoplasm and limiting the motion of macromolecules. Increasing evidence is highlighting the contribution of physicochemical determinants in tuning coacervation of macromolecules, suggesting that phase-separating biomolecules harbor sequence-encoded sensors responsive to environmental changes[17, 18]. Indeed, the time-dependent material properties of phase-separated chromatin condensates regulate their function and biophysical interplay with the surrounding nuclear components. From a material science perspective, the nucleus is a heterogeneous viscoelastic medium whose mechanical and rheological properties vary across scales. In this work, we investigated how the biophysical properties of chromatin and the exerted mechanical forces are perceived and decoded by chromatin condensates. In this respect, the findings that the transcript levels of genes codifying for the chromatin factors were unchanged in response to mechanical stress facilitated our analyses and data interpretation.

2. Why is figure 1 including identical data (and samples?) imaged on confocal and super resolution microscope? What is the motivation there? This seems redundant.

Response:
We included both confocal and SIM imaging data to strengthen the reproducibility of the obtained results, independent of the achieved resolution and acquisition modalities. Indeed, the post-processing image data analyses for identifying condensates can be strongly influenced by the signal-to-noise ratio and by the optical resolution, which is limited to 250-300nm in a scanning confocal setting. To ensure that the measured differences in the numerosity of condensates were not affected by the mentioned intrinsic limitations of acquisition modality, we repeated the measurements on an independent set of biological replicates by SIM microscopy. The obtained results confirmed the increase of transcriptional condensates in response to ECM stiffening, while PcG condensates showed an opposite response (Figure 1A-D).

3. An alternative approach to measure nuclear/chromatin and matrix stiffness should have been employed to supplement Brillouin microscopy studies - this is the standard in the field as all methods have limitations.

Response:
We acknowledge Reviewer #3's suggestion that comparing nuclear stiffness measurements obtained via Brillouin Microscopy (BM) with complementary techniques like Atomic Force Microscopy (AFM) is generally appropriate. However, in this particular case, such a comparison is neither feasible nor meaningful due to the limitations of other approaches in measuring nuclear mechanical properties with the required spatial resolution (x, y, z).
To accurately assess the contribution of chromatin to nuclear stiffness it is essential to reach sub-micrometric resolution along the x, y, and z axes, as well as the ability to scan the entire nuclear volume in 3D. Brillouin microscopy offers high spatial resolution and sensitivity by focusing light within microscopic dimensions, making it superior to bulk rheological or mechanical techniques that provide only averaged 2D measurements over large areas. The alternative methods lack the ability to achieve the desired sub-cellular resolution. In contrast, Brillouin microscopy enables the resolution of mechanical properties at a sub-micrometer scale in 3D, enabling precise mapping of mechanical heterogeneity within the cell nucleus.

Unlike AFM or nanoindentation, BM is a purely optical technique that does not require mechanical contact or the application of external force to the sample. While AFM and nanoindentation involve physical interaction with the

sample (often through surface deformation or mechanical probing), BM uses light scattering to non-invasively probe mechanical properties. This minimizes the risk of damaging delicate biological structures, such as cell nuclei. Moreover, AFM and nanoindentation typically provide indirect measurements of the nucleus's elastic modulus, often limited to surface deformations, which may not fully capture the internal mechanical characteristics of the nucleus.
In summary, Brillouin microscopy's combination of high sensitivity, spatial resolution, and the non-contact light scattering approach uniquely enables the direct measurement of chromatin-dependent mechanical properties in both cells and tissues. This capability is particularly valuable, as we have demonstrated BM's spatial precision in distinguishing the mechanical contributions of cell nuclei versus the extracellular matrix (ECM) to cartilage stiffness within the growth plate. This unique feature makes BM the only appropriate method for accurately assessing nuclear mechanical properties in stiff tissues, as supported by recent findings[19]. Therefore, we believe that a comparison with other conventional techniques would neither be feasible (since no comparable information would be obtained) nor enhance the significance of our work.

4. Figure 2 A and B panels appear to be mixed as they do not match figure legends.

Response:
We apologize with the Reviewer for exchanging the figure legends, and we corrected it in the revised manuscript.

5. Many of quantifications in 3 do not seem to match representative images - all of the conditions appear to have a very similar amount of condensates - I just do not see how one gets to quantification presented in 3C. Either the representative images or quantification are incorrect

Response:
We apologize for not properly choosing representative images matching the quantifications of the dynamic assembly of OptoMLL4 condensates in response to blue light stimulation. After verifying the image quantification procedures that confirmed the statistical significance of the observed changes in the numerosity and sizes of assembled condensates (Figure 3C, D, and Figure S3D), we selected alternative images that better convey the information obtained from quantifying multiple cells under biologically independent conditions. We would like to emphasize that the representative images currently provided show only two time points (before and after light stimulation). As such, they can not fully capture the dynamic changes in condensate number and size that occur in response to light stimulation, nor their dependence on genetic background or substrate stiffness. This is why we quantified these parameters throughout the time-lapse imaging process. Overall, we believe that the improvement of the visualization of MLL4 PrLD clusters helps in supporting the quantitative analyses detecting the dynamic changes in condensate assembly in response to blue-light stimulation.

6. Data in 3G is a bit puzzling ie confinement not inducing condensation - if nuclear mechanics is the proposed mechanism, which is logical, all the conditions should from condensates as confinement triggers chromatin remodeling and condensation.

Response:
In Figure 3G, we showed that the degree of cell confinement significantly influences the nuclear mechano-response, particularly regarding the assembly of MLL4 condensates. Our findings indicate a non-linear response to mechanical load, characterized by a threshold-dependent mechanism. This suggests that only when mechanical load exceeds a certain threshold do we observe the transient modulation of nuclear mechanical properties, which is necessary for the formation of MLL4 condensates. These results align with previously described mechanisms involving other mechano-sensors and the nucleus itself, which can initiate a feedback mechano-response in a threshold-dependent manner[20]. In addition, considering that the assembly of condensates relies on phase transitions, including LLPS, which occurs when the local concentration of macromolecules overcomes a certain threshold, our findings indicate that the degree of confinement impinges on the biophysical properties of MLL4 and its clustering into condensates.

7. Figure 4 legends has two Es and panel F is missing in figure legends.

Response:
We apologize for the mislabeling and we corrected it in the revised manuscript.

8. The quality of 3D imaging appears of relatively low quality (for volume quantification; phenotype is clear and already published in their previous paper) the resolution in z is clearly quote low and segmentation would be very challenging - how did the authors make their measurements (simple statement of "3D analysis was performed by using the 3D plugin suite" is not sufficient)? It is unlikely that the data in 5D, 5C would reach significance; I am not sure this difference is below segmentation error.

Response:
Regarding the quality of the 3D reconstructed images, we acknowledge the differences noted compared to our previous work8, in which we did not measure potential changes in nuclear shape in response to external mechanical forces. To improve the image quality, we increased the axial image segmentation by acquiring z-step size di 0.1um (using Nikon Ti2 Eclipse, in scanning confocal modality, equipped with CFI Apo SR HP TIRF 100XC/1.49 objective), combined with 3D volume imaging rendering using the 3D viewer plugin from ImageJ (Figure 5B). In the Methods section, we further detail the adopted procedures to retrieve the nuclear-associated measurements.
Concerning the statistical significance of the measurements in the nuclear area, we re-analyzed the data, considering the independent biological replicates to appropriately determine the sample size (n) for our analyses. For this comparison, to assess the effects of genotype, stiffness, and nuclear volume (or area), we employed linear mixed-effects models. Specifically, genotype and stiffness were treated as fixed effects, while replicates were considered as a random intercept to account for repeated measurements. Model fitting was performed using Restricted Maximum Likelihood (REML). We report the p-values associated with interaction terms (genotype × stiffness) to identify context-specific genotype effects across stiffness conditions.

9. In 4E FRET efficiency images are not convincing and quantifications really showing lack of significance if proper statistics were done.

Response:
The Nesprin1-based FRET sensor is specifically designed to measure cytoskeletal forces acting on the nuclear envelope, and the results we obtained are consistent with prior studies using the same sensor[21, 22].
We carefully rechecked the representative images and ensured that they correspond correctly to the analyzed samples, shown in Figure 5F of the revised manuscript. Possibly, the impression that the signal intensities vary modestly among samples is due to the chosen color scale, yet it recapitulates the quantified values and the measured differences as an Inverted FRET index (2-fold increment in response to ECM stiffness).
Regarding the quantifications, we have re-analyzed the data, using the number of independent biological replicates as n, and statistical significance was determined by Sidak's multiple comparison test (two-way ANOVA). The obtained results showed that the increase in ECM stiffness caused an increase in cytoskeletal-transmitted forces acting on the nuclear envelope. However, we did not measure the same increment in the MLL4 LoF MSCs. These results are in line with the new set of data showing that in MLL4 LoF MSCs (a) genes involved in cell adhesion and cytoskeleton organization were downregulated (Figure S6D); (b) the contractile perinuclear actin cap was reduced (Figure S6F); while in wild-type MSCs (c) the cytoskeleton-nucleus mechanical uncoupling thought the overexpression of the dominant negative GFP–Nesprin-1/2–KASH protein increased the frequency of NE rapture (Figure S6G). Overall, we are confident that the improvement in the data quantification strengthens the significance of the retrieved results, supporting the notion that chromatin condensate balancing contributes to determining the nuclear mechanics, which is coupled to the cytoskeleton-transmitted external forces.

10. Further, similarly to stiffness measurements, authors need to properly measure tension on the nuclear envelope versus tension across nesprin to make any conclusions with respect to nuclear mechanics.

Response:
We appreciate the Reviewers' insightful observation regarding the FRET tension sensor utilized in this study, which is based on Nesprin1, a key component of the LINC complex. As noted, Nesprin1 spans from the inner nuclear envelope, where it interacts with SUN domain-containing proteins, to the cytoplasm, where it binds to actin. Consequently, this sensor is sensitive only to forces exerted by the cytoskeleton on the nuclear envelope and does not detect tension parallel to the membrane[21].
While we believe that this system serves as a valuable proxy for measuring nuclear envelope tension, we acknowledge the need for clarification. To properly describe the measurements retrieved from the FRET experiments, we modified the text throughout the manuscript to reflect this nuance, changing references from "NE tension" to "measurements of the cytoskeletal forces acting on the NE." To avoid overinterpretation of the results,

we also changed the paragraph title in "Unbalancing chromatin condensates affects nuclear shape in response to mechanical cues". We thank the Reviewer for the constructive feedback, which enhances the clarity of our work.

11. As mentioned above, Figure 6A is the first proper graph with means of independent experiments on which statistical analyses can be done at first sight, however what the authors then go on to do is instead of comparing the 6 conditions together, instead of doing proper statistical analyses which would likely yield lack of statistical significance, they perform 3 pairwise comparisons, which is not appropriate

Response:
To address the criticism, we treated the four analyzed conditions as dependent events and conducted an ANOVA statistical test to evaluate the differences between the means of the multiple groups. The obtained results, illustrated in Figure 6A of the revised manuscript, confirmed the statistical robustness of our findings related to the increment of spontaneous NE rupture in MLL4 LoF MSCs. Additionally, we repeated the ANOVA analyses for the data presented in the associated supplementary Figure S6A-C and S6G. Similarly, we measured the statistical significance of data represented in Figure 6G, 6I, S6J, and S6L by two-way ANOVA. The obtained results confirmed the robustness of our results related to the increased sensitivity of MLL4 LoF MSCs to mechanical stress, resulting in an increase in NE fragility.

12. Further neither the images nor quantifications show a clear effect an effect on cGas foci - authors may consider just showing cGas and removing the nuclear counterstain

Response:
To improve the visualization of the cGAS foci in MSCs grown on soft and stiff substrate matrices, we showed the images of the cGAS immunofluorescence separated from the nuclei staining (Figure 6A), as suggested. We also re-determined the statistical significance of the measured differences in the four experimental conditions, using Sidak's multiple comparison test (two-way ANOVA).

13. Migration movies and the way figures relating to cellular migration (3I, many panes in figure 6), are presented make them very hard to assess - many more time points would be necessary to assess the effects - in the present form it is impossible to assess the phenotypes.

Response:
We appreciate the feedback regarding the inclusion of multiple time points in the figure panels to enhance the clarity of the assessed effects. We improved the representative image qualities, highlighting the dynamic formation of MLL4 PrLD condensates during MSCs migrating through microchannel constrictions of different heights (Figure 3J). We think that the representative images better represent the measured increment of MLL4 PrLD condensates in response to nuclear deformation. In addition, we also improved the image representation of the NE rupture events by showing multiple image time points of the cellular distribution of NLS-GFP and cGAS clustering in MSCs migrating through the microchannel constrictions (Figure 6E-J and Figure S6I, K). We thank the Reviewer for the constructive comment that helped us improve the quality of the representative images to deliver our findings better.

14. Figure 4 is poorly explained and motivation to a non-expert and breaks up a flow int the paper - consider editing to better integrate.

Response:
We apologize for not conveying the motivation behind our use of a molecular dynamics approach to explore the possible mechanism by which MLL4 might function as a mechano-sensor through conformational changes. We better motivated our choice in the revised manuscript, emphasizing the well-established molecular mechanisms by which mechano-sensors respond to mechanical forces by conformational changes[23, 24]. This will help underscore the importance of the molecular insights gained from our study.
Nevertheless, in the revised manuscript, we also included a new set of data to experimentally verify our computational prediction, by means of mutagenizing the interspersed Leucine within the MLL4 PrLD, which we predicted to experience conformational transition in response to mechanical load. (Figure 4A-F of the revised manuscript). We found that impairing the conformational transitions abrogated the mechano-response of MLL4 PrLD (Figure 4G), similarly to the observed response upon deletion of the PolyQ stretch (Figure. 3I). We believe that these new experiments clarify the relevance of investigating the role of conformational changes occurring in response to mechanical cues, thus supporting the MLL4 function as a mechano-sensor.

Regarding data accessibility for non-experts, we recognize that the current manuscript may not be suitable for a general audience unfamiliar with these methodologies. We revised the text to clarify complex concepts and improve readability. Additionally, we discussed the significance of adopting multidisciplinary approaches to investigate how macromolecules contribute to nuclear mechano-signaling.

15. Authors state that "By analyzing the nuclear morphology, we found that the nuclear area, volume, and flatness were increased in cells maintained on a stiff matrix, and this response was weakened upon MLL4 LoF" - figure 5E would indicate that LoF MLL4 cells exhibit decreased cell spread area on both soft and stiff substrates? However Figugre 1 would suggest the opposite as MLL4 LoF representative images have comparatively large nuclei on stiff substrates.

As the reviewer correctly pointed out, we stated that the increased nuclear area in response to augmented substrate stiffness is diminished in the MLL4 LoF condition, which is reflected in the representative images shown in Figure 1. Indeed, we observed a relative impairment of the nuclear response to increased substrate stiffness in MLL4 LoF cells compared to wild-type cells, and the images in Figure 1 aim to convey this same observation. That said, we carefully re-evaluated the presented images and the corresponding quantifications to ensure they accurately represent the data and maintain high standards of quality and reproducibility. We have now changed the representative images of Figure 5E to properly match them with the quantitative measurements of excess of perimeter and nuclear morphology (Figure 5C, 5E).

16. What does the re-establishment of the PcG condensates via H3.3K27M mutant do to nuclear mechanics (stiffness and NE tension)?

Concerning the contribution of PcG condensates to nuclear mechanics, we have already assessed this through Brillouin microscopy measurements on MSCs grown on both soft and stiff substrates (Figures 1E and F). The results demonstrated that PcG condensates play a significant role in determining the mechanical properties of chromatin. Indeed, we found that the expression of the H3.3 K27M mutant, which has a dominant negative effect on PcG activity and compartmentalization, alleviated the increment of chromatin stiffness characterizing MLL4 LoF MSCs cultured on stiff substrates. In addition, by rescuing the augmented PcG condensates MLL4 LoF MSCs, we re-established the nuclear shape and the relative abundance of Lamin A/C (Figure 5D and S5F), ameliorating NE integrity with the consequent dampening of cGAS/STING activation and the associated cell death (Figure S7J, S7L).

However, we acknowledge that we can not distinguish between the biochemical or mechanical contribution of H3.3 K27M mutant, and unfortunately, there are no available tools to address it appropriately. We discussed this point as a current technical limitation of our study.

References:

1. Fujiwara, K., Toda, H. & Ikeguchi, M. Dependence of α-helical and β-sheet amino acid propensities on the overall protein fold type. *BMC Struct Biol* **12**, 18 (2012).
2. Zhao, J.Z., Xia, J. & Brangwynne, C.P. Chromatin compaction during confined cell migration induces and reshapes nuclear condensates. *Nat Commun* **15**, 9964 (2024).
3. Todorovski, V. *et al.* Confined environments induce polarized paraspeckle condensates. *Commun Biol* **6**, 145 (2023).
4. Joo, Y.K. *et al.* ATR promotes clearance of damaged DNA and damaged cells by rupturing micronuclei. *Mol Cell* **83**, 3642-3658.e3644 (2023).
5. Kovacs, M.T. *et al.* DNA damage induces nuclear envelope rupture through ATR-mediated phosphorylation of lamin A/C. *Mol Cell* **83**, 3659-3668.e3610 (2023).
6. Swift, J. *et al.* Nuclear lamin-A scales with tissue stiffness and enhances matrix-directed differentiation. *Science* **341**, 1240104 (2013).
7. Engler, A.J., Sen, S., Sweeney, H.L. & Discher, D.E. Matrix elasticity directs stem cell lineage specification. *Cell* **126**, 677-689 (2006).

8. Fasciani, A. *et al.* MLL4-associated condensates counterbalance Polycomb-mediated nuclear mechanical stress in Kabuki syndrome. *Nat Genet* **52**, 1397-1411 (2020).
9. Zhao, Z. *et al.* Somatic mutations of MLL4/COMPASS induce cytoplasmic localization providing molecular insight into cancer prognosis and treatment. *Proc Natl Acad Sci U S A* **120**, e2310063120 (2023).
10. Collins, R.L. *et al.* A cross-disorder dosage sensitivity map of the human genome. *Cell* **185**, 3041-3055.e3025 (2022).
11. Yang, L. *et al.* Phase separation as a possible mechanism for dosage sensitivity. *Genome Biol* **25**, 17 (2024).
12. Veitia, R.A. Emerging links between phase separation and transcription factor haploinsufficiency. *Trends Genet* **40**, 998-1000 (2024).
13. Elosegui-Artola, A. *et al.* Force Triggers YAP Nuclear Entry by Regulating Transport across Nuclear Pores. *Cell* **171**, 1397-1410.e1314 (2017).
14. Sladitschek-Martens, H.L. *et al.* YAP/TAZ activity in stromal cells prevents ageing by controlling cGAS-STING. *Nature* **607**, 790-798 (2022).
15. Nava, M.M. *et al.* Heterochromatin-Driven Nuclear Softening Protects the Genome against Mechanical Stress-Induced Damage. *Cell* **181**, 800-817.e822 (2020).
16. Killaars, A.R., Walker, C.J. & Anseth, K.S. Nuclear mechanosensing controls MSC osteogenic potential through HDAC epigenetic remodeling. *Proc Natl Acad Sci U S A* **117**, 21258-21266 (2020).
17. Villegas, J.A., Heidenreich, M. & Levy, E.D. Molecular and environmental determinants of biomolecular condensate formation. *Nat Chem Biol* **18**, 1319-1329 (2022).
18. Delarue, M. *et al.* mTORC1 Controls Phase Separation and the Biophysical Properties of the Cytoplasm by Tuning Crowding. *Cell* **174**, 338-349.e320 (2018).
19. Martinez-Vidal, L. *et al.* Progressive alteration of murine bladder elasticity in actinic cystitis detected by Brillouin microscopy. *Sci Rep* **14**, 484 (2024).
20. Lomakin, A.J. *et al.* The nucleus acts as a ruler tailoring cell responses to spatial constraints. *Science* **370** (2020).
21. Poli, A. *et al.* PIP4K2B is mechanoresponsive and controls heterochromatin-driven nuclear softening through UHRF1. *Nat Commun* **14**, 1432 (2023).
22. Pennacchio, F.A. *et al.* N2FXm, a method for joint nuclear and cytoplasmic volume measurements, unravels the osmo-mechanical regulation of nuclear volume in mammalian cells. *Nat Commun* **15**, 1070 (2024).
23. Wojciechowski, M., Szymczak, P., Carrión-Vázquez, M. & Cieplak, M. Protein unfolding by biological unfoldases: insights from modeling. *Biophys J* **107**, 1661-1668 (2014).
24. Saini, K. & Discher, D.E. Forced Unfolding of Proteins Directs Biochemical Cascades. *Biochemistry* **58**, 4893-4902 (2019).

UNIVERSITÀ DI TRENTO

Department of Cellular, Computational and Integrative Biology - CIBIO

Università di Trento,
Dipartimento CIBIO
Via Sommarive 9, POVO

Trento, 11th of September 2025

Reviewer comments:

Reviewer #1:
The authors have tried to address my questions by experiments and analyses. They have partially addressed some of my questions with new data. Despite the authors' efforts, the molecular mechanisms by which MLL4 condensates mediate mechano-response still remain a major unanswered question, largely due to the understandable technical difficulty in the field. The authors have added sufficient discussions to reflect these unaddressed questions.

We are pleased that Reviewer #1 recognized our effort to address all the raised criticisms and acknowledged that technical limitations in the field do not permit us to directly determine which regions and activities of MLL4 mediate the mechano-phenotypes following mechano-sensing. Indeed, as discussed in our rebuttal letter, to reach any valid conclusion, it would be necessary to rescue the MLL4 LoF by expressing either a methyltransferase-dead mutant versus the full-length protein, or a PrLD-deleted form of MLL4. However, these experiments are particularly challenging because MLL4 is a large protein (5,537 amino acids), making it difficult to express it to a similar level as the endogenous gene, thus properly rescuing its function. That said, we performed additional experiments to demonstrate that the MLL4 prion-like domain (PrLD) plays a functionally specific role in mechano-sensing. Indeed, we have performed molecular dynamics (MD) simulations of the polyQ-rich stretch within the PrLD, which showed that under increasing simulated mechanical pressure, it changes its conformation, forming β-sheet-rich structures. This transition mirrors the pattern of mechanosensitive domains, which respond to mechanical load by changing their secondary structure24, 25. To experimentally validate this prediction, we substituted the interspersed Leucine with Alanine (thus abrogating the conformational transition). We tested the resulting MLL4-PrLD variant for its clustering into condensates in response to cell confinement. While this mutant showed the same propensity to assemble into condensates in response to blue-light stimulations, it did not form condensates upon cell confinement, closely phenocopying the effects of polyQ deletion. Based on these results, we concluded that the PrLD of MLL4 is necessary for its mechano-sensing. However, we can not exclude that other domains or functions of MLL4 can contribute to its mechano-responsiveness. As originally requested by the Reviewer ("It is worthy of at least some discussion"), we discuss this specific point in the discussion section of the revised manuscript, as follows:
"Future efforts aimed at developing strategies to selectively perturb condensate organization without affecting catalytic activity (and vice versa) will be essential to fully dissect their individual contributions to nuclear mechanics and chromatin-based mechanoresponsiveness."

The current data and writing do not make it easy to understand how overexpression of H3.3K27M restores PcG condensates in MLL4 LoF cells, and how that restores the mechano-phenotypes, since MLL4 PrLD seems to have relative unique properties to mediate mechano-sensing. This is pertinent to the central question in this paper.

We apologize for not having properly clarified how H3K27M restores PcG condensates. "To rule out the contribution of PcG condensates to the altered responsiveness of the MLL4Q4092X MSCs, we rescued its functionality by overexpressing the H3.3 K27M mutant, which has a dominant-negative effect on PcG activity and compartmentalization (Figure S1M)10. Indeed, we referred to our previous work in which we demonstrated that the overexpression of H3.3K27M, which represent a cancer-specific point mutation that alters the biochemistry of PRC2–chromatin interactions, and the associated histone-mark landscape, affects the nucleation and the multivalent interactions occurring among the PRC1/cPRC components, thus reducing the assembly of Polycomb condensates Regarding the request to clarify better the mechanism by which restoring PcG condensates rescue the nuclear mechanics, this point has been already addressed in our previous work (Fasciani A. et al., Nat Genetics 2020) in which we demonstrated using and optogenetic approach that the increased assembly of PcG condensates is sufficient to drive the mechano-related phenotype by increasing the chromatin stiffness. <u>We can further clarify this point within the manuscript's text, without the need to address it experimentally.</u>

That said, the Reviewer did not previously raise these comments, so it was difficult for us to address them accordingly. Indeed, the Reviewer previously asked us to comment on the limitations associated with the H3K27M strategy, which cannot correctly distinguish between the effects on PcG enzymatic activity and chromatin compartmentalization. As previously mentioned, unfortunately, no available methods can effectively separate the (epi)genetic functions from the mechanical functions, and these mechanisms are likely interrelated, further complicating the analysis.
We discuss this specific point in the discussion session, as requested by the Reviewer:
"*It is important to note that while the use of the H3.3 K27M mutant allowed us to probe the involvement of PcG associated domains, this approach does not fully disentangle the epigenetic effects of PcG enzymatic activity from the biophysical and mechanical contribution of PcG condensates. These two mechanisms are likely interdependent, and the current lack of tools to separate them represents an inherent limitation of the study*."

In sum, we can adequately address these points by describing the mechanism by which the overexpression of H3K27M can rescue the clustering of PcG and its impact on the nuclear mechanics, referring to our previous work.

Reviewer #2:
The authors addressed all the questions raised by the reviewers. I am happy to accept this manuscript for publication in *[journal name redacted]*.

We are pleased that the Reviewer#2 appreciated our revised manuscript in which we included all the arranged experiments to address his/her criticisms.

Reviewer #3:
The authors did a reasonable job better motivating Kabuki syndrome work and highlighting the group's previous work in a very similar topic - the manuscript is definitely much improved.

We are pleased that Reviewer#3 acknowledged our effort in designing multiple approaches to address all the raised (fourteen) points adequately and found the revised manuscript much improved.

I did find the authors comments regarding their findings "go beyond the current stage of understanding the role of the nucleus in mechanobiology, which has been considered limited to the involvement of the nuclear lamina and the interconnection with the cytoskeleton through the LINC complex." quite upsetting as it illustrates lack of knowledge of relatively large body of literature linking chromatin to nuclear mechanics, many of which the authors site.

We apologize if our comment was not entirely appropriate, and we did not intend to undermine the relevance of previous works addressing the potential contribution of chromatin to the nuclear mechanical properties. Indeed, our comment referred to the added value of our work as "contributes significantly to defining the role of chromatin condensates beyond their potential functions in gene expression and DNA damage response, as acknowledged by Reviewers #1 and #2. Furthermore, our findings open new perspectives in the field of mechanobiology by highlighting the importance of chromatin organization in determining nuclear mechanical properties and the mechanical balance in response to external forces." The latter sentence referred to our comment in which we stated that "we demonstrated that nuclear integrity relies on the balance between transcriptional condensates and PcG condensates. When this equilibrium is disrupted, as in the case of KS characterized by MLL4 LoF, the nuclei exhibit increased nuclear envelope rupture and subsequent activation of the cGAS/STING signaling pathway in response to mechanical stress."
Considering the context of the criticized comment, we did not overestimate the relevance of our finding nor undervalue the importance of previous works linking chromatin to nuclear mechanics. As this comment was not part of the manuscript (but just in the rebuttal letter), it should not be considered a valid point to reject our work.

Statistical section got improved although I remains skeptical of chosen statistical methods and significance given how the raw data looks -furthermore, precise n numbers should be indicated never >= and for all data specific numbers of cells should be indicated.

We are pleased that Reviewer#3 acknowledged our effort in adequately addressing the raised criticism related to the statistical analyses, as requested. Indeed, we performed a Tukey's and Sidak's multiple comparison test (two-way ANOVA) evaluating the differences between the means of the multiple groups, considering the analyzed conditions as dependent events (genotypes and mechanical loads). To analyze morphometric features (Area, Excess of Perimeter, Flatness, Volume, and material properties), we employed a linear mixed-effects model by treating genotype and stiffness as fixed effects, while replicates were considered as a random intercept accounting for repeated measurements. Model fitting was performed using Restricted Maximum Likelihood (REML). Furthermore, we increased the numerosity of the biological replicates to improve the consistency and reproducibility of the measured differences. It is quite challenging to address the Reviewer's

comment (*I remains skeptical of chosen statistical methods and significance given how the raw data looks*) as it does not refer to any specific scientific points/notions that we could properly address to improve the statistical analyses further. Furthermore, after carefully checking the revised manuscript, we are confident that we have consistently reported the correct number of the analyzed biological replicates and never used the >= symbol, except in the rebuttal letter, in which we indicated that the number of analyzed replicates is n>3 in most of the experiments.

Image analysis section did not improve much - one cannot use the methods to reproduce authors analysis, especially as it relates to analyses of 3D shape.

We are sorry that the Reviewer was not fully satisfied with the "Image analysis" section reported in the Methods, as we followed the previous request to improve it by detailing the adopted procedures to reconstruct the 3D nuclear images and the associated measurements (volumes). In the revised manuscript, we reported the step-by-step procedure we normally employ to retrieve this information using the 3D plugin suite of ImageJ. We are confident that the described procedure permits the reproduction of the performed analyses on the nuclear volume. Nevertheless, we can easily improve the description of this standard methodology further, as requested.

Most importantly, I only requested one experiment which was to complement mechanical measurements with AFM and the authors did not perform.

Regarding the request to confirm the measurement of the nuclear stiffness by AFM, we argued extensively in the rebuttal letter about the motivations for which we did not proceed with such analyses. Indeed, although we agree that confirming the measured changes in chromatin stiffness using an orthogonal approach would be beneficial, AFM (or a similar approach) does not have the requested spatial resolution to distinguish the chromatin contribution to the nuclear mechanics properly. This is a very relevant point that limits the applicability of AFM to properly distinguish the contribution of chromatin from the nuclear lamina to the nuclear mechanical properties. In addition, AFM provides indirect measurements of the nucleus's elastic modulus E, as it probes the cell surface deformations, which cannot uniquely depict the nuclear mechanical properties.
That said, we can underline the potential limitations associated with not having employed an orthogonal measurement of chromatin mechanics in the "Limitations of the study" section of the manuscript.

Overall I remain unenthusiastic about the work and not fully convinced of the findings but the manuscript has improved and there are interesting observations within the work that would be of interest to the community.

We are pleased to notice that, despite these criticisms, the Reviewer#3 underlined the improvement of the revised manuscript and acknowledged its importance for the scientific community. We can smooth some of these comments by improving the textual description of the methodologies for image analysis and by underling some intrinsic limitations of the study.

Dear Prof. Zippo,

Thank you for submitting your manuscript for consideration by the EMBO Journal. It has now been seen by an arbitrating referee whose comments are shown below.
As you can see from the report, the referee finds the manuscript interesting and appreciates the revisions undertaken during the previous round. However, the referee also identifies a number of important points that would still need to be addressed for publication in The EMBO Journal.

Given your willingness to commit to major revisions addressing the reviewer's comments, I would like to invite you to submit a revised manuscript based on the preliminary revision plan you have shared with us. Please note that The EMBO Journal's policy allows only a single round of revision, and acceptance of your manuscript will therefore depend on the completeness and quality of your responses in this revised version.

When preparing your letter of response to the referees' comments, please bear in mind that this will form part of the Review Process File, and will therefore be available online to the community. For more details on our Transparent Editorial Process, please review our Editorial Policies: https://link.springer.com/partners/embo-press/editorial-policies

We generally allow three months as standard revision time. As a matter of policy, competing manuscripts published during this period will not negatively impact on our assessment of the conceptual advance presented by your study. However, we request that you contact the editor as soon as possible upon publication of any related work, to discuss how to proceed. Should you foresee a problem in meeting this three-month deadline, please let us know in advance and we may be able to grant an extension.

Thank you for the opportunity to consider your work for publication. I look forward to your revision.

Yours sincerely,

Cornelius Schneider, PhD
Editor
The EMBO Journal
c.schneider@embojournal.org

Instructions for preparing your revised manuscript:

Read our guidance for manuscript revisions and related editorial policies: https://link.springer.com/journal/44318/submission-guidelines#cms-Revised-submissions

Please make sure you upload a letter of response to the referees' comments together with the revised manuscript.

Please also check that the title and abstract of the manuscript are brief, yet explicit, even to non-specialists.

When assembling figures, please refer to our figure preparation guideline in order to ensure proper formatting and readability in print as well as on screen:
https://media.springernature.com/original/springer-cms/rest/v1/content/27825798/data/v1

At EMBO Press we ask authors to provide source data for the main manuscript figures. You will receive a separate email with instructions for providing source data with your revised manuscript, including how to upload and organize the files.

IMPORTANT: When you send the revision we will require
- a point-by-point response to the referees' comments, with a detailed description of the changes made (as a word file).
- a word file of the manuscript text.
- individual production quality figure files (one file per figure)
- a complete author checklist
- Expanded View files (replacing Supplementary Information)
- a Reagents and Tools Table as part of the Methods section

Please remember: Digital image enhancement is acceptable practice, as long as it accurately represents the original data and conforms to community standards. If a figure has been subjected to significant electronic manipulation, this must be noted in the

figure legend or in the 'Materials and Methods' section. The editors reserve the right to request original versions of figures and the original images that were used to assemble the figure.

We realize that it is difficult to revise to a specific deadline. In the interest of protecting the conceptual advance provided by the work, we recommend a revision within 3 months (23rd Apr 2026). Please discuss the revision progress ahead of this time with the editor if you require more time to complete the revisions.

Referee #1:

The authors have submitted a revised manuscript that addresses many of the previous reviewers' comments. Nonetheless, a number of questions and concerns remains. In addition, this reviewer, who was not involved in the previous review, also noticed a few additional items that should be addressed by the authors.

As the three reviewers previously stated, the manuscript would benefit from untangling further the mechanism by which MLL4 loss of function results in the various effects, particularly whether specific effects are directly downstream of altered condensation and chromatin mechanics, or potentially downstream of altered gene expression or other mechanotransduction pathways. The transcriptomic analysis and YAP/TAZ overexpression (Figures 6 and S6) indicate that at least some of the nuclear rupture effects occur downstream of other mechanotransduction pathways and not necessarily as a direct consequence of altered nuclear stiffness). The authors are commended for already including several new experiments in the revised manuscript that go towards answering this question. A more careful analysis of the temporal sequence and dynamics of effects, for example, in response to nuclear confinement or acute MLL4 depletion using the degron system, could substantially advance the current interpretation. Given that the authors have the tools available to disentangle the direct vs. indirect effects of MLL4 LoF, using for example at least some basic molecular profiling in combination with acute depletion of MLL4, and thus temporally separate primary from secondary effects, it is surprising that they have not taken advantage of these capabilities yet. Right now, the relatively long duration for some of the perturbations (30 min for nuclear confinement, 8(?) hours for the degron system), and 48 hours for substrate stiffness allows for many secondary effects to occur that make the interpretation of the results more challenging.

In their response to reviewer #3, the authors state that "we demonstrated that nuclear integrity relies on the balance between transcriptional condensates and PcG condensates". In that case, would the authors expect that any shift in the balance between transcriptional condensates and PcG would result in loss of nuclear integrity? This should be tested experimentally.

I do share the concerns of Reviewer #3 about the need to validate Brillouin microscopy measurements of nuclear stiffness using orthogonal approaches such as AFM, nanoindentation, nuclear stretching, or micropipette aspiration. These techniques have all been used successfully to demonstrate changes in nuclear stiffness in response to altered chromatin organization. While Brillouin microscopy does offer the advantage of spatial resolution and contactless measurements, it is also more prone to measurements being affected by confounding factors such as water content, cell morphology, etc. These factors further complicate the interpretation of Brillouin phase shift measurements and translating them into corresponding changes in material properties. Since the authors report Brillouin shift measurements averaged across the entire nucleus and do not try to determine subnuclear differences in nuclear mechanics, their results should also be detectable by bulk nuclear stiffness measurements using other technologies. The in vitro experiments using MSCs should be well suited for such studies.

In their characterization in Figure 1 and S1, the authors provide information on BRD4, BMI, and PcG cluster number, size, and total fluorescence intensity, and state that the transcript number of MLL4 is unchanged in the MLL4 LoF cells. Since transcript number doesn't always correlate with protein levels, the authors should also confirm MLL4 protein levels at the different substrate conditions by Western blot analysis.

The graph for the results of the confinement experiments shown in Fig. 3G, H is missing a crucial comparison, the data for the MLL4 LoF cells under 3 um confinement, given that only 3um confinement elicited an increase in MLL4 condensates. If the authors experienced increased rates of cell death under those conditions, they could still try to analyze the live MLL4 LoF cells under the 3um confinement conditions. In addition, the authors should consider including short timepoints, as presumably the confinement itself is achieved within minutes (or faster), and condensation should occur on similar or shorter time scales. This would enable them to further discern whether the confinement induced condensation is primarily driven by changes in nuclear geometry-and likely volume-or potentially downstream of other mechanoresponsive factors. In addition, the authors should test whether other proteins such as BMI form increased or increased condensates under confinement.

In the confinement experiments shown in Suppl. Fig. S3F, it appears that the cell density of the MLL3 LoF cells in the 3um confinement condition is substantially lower than in the other conditions (only four nuclei are visible in the field of view, compared to ~8 nuclei/FOV for the other conditions), which could affect the extent of confinement and survival. The authors

should comment on this discrepancy and confirm whether experiments were carried out at similar cell densities.

In the confinement experiments shown in Suppl. Fig. S3H, I, it seems very surprising that the cross-sectional area of the nuclei subject to 3um confinement is substantially smaller than that of the unconfined nuclei, since typically confinement results in an increase in nuclear cross-sectional area. The authors should explain this unexpected finding.

LINC complex disruption using dominant negative nesprin or SUN domain constructs is highly dependent on the expression levels. For the experiments using the C1-KASH construct, the authors should thus confirm successful LINC complex disruption by demonstrating displacement of endogenous nesprins from the nuclear envelope.

While the molecular dynamics simulations are informative, in the current form, the description in the Results section lacks sufficient detail for a reader to interpret the results. The authors should comment on how the increased pressure was modeled in the simulation and how the applied pressure (1, 1.5, or 1.75 atm) compares to experimental conditions. Otherwise, it is difficult to discern whether the applied settings correspond to physiological conditions.

For the measurements of lamin A/C levels (Fig. 5), the authors should confirm altered lamin A/C levels by western blot analysis, given that antibody binding to lamin A/C can depend vary substantially based on mechanical stress (PMID 26301768) and cell density (PMID 36809122). Furthermore, this would also enable the authors do distinguish between the lamin A and C isoforms. For all experiments, the authors should further include measurements of lamin B levels, which are less sensitive to substrate stiffness (PMID 23990565), as a control.

In the description of the experiments with the MLL4 HaloPROTRAC3 system, the authors should include the exact treatment duration, instead of just referring to "by shortly treating" in the text. Right now, neither the main text or the figure legends include this important information about the treatment duration, which is crucial for the interpretation. The Method section indicates "for 8/24 hours", which does not provide sufficient clarify either.

The authors interpretation that "maintaining nuclear integrity requires a proper balancing between inward, nuclear-generated and outward, ECM-mediated mechanical forces." (Introduction) and "applied outward forces are balanced by chromatin-mediated inward forces to preserve nuclear morphology and genome integrity" (Response to reviewers) is too simplistic and likely inaccurate. In mesenchymal cells, cytoskeletal forces likely apply both compressive forces (via apical stress fibers and the 'actin cap') and tensile forces to the nucleus. Intranuclear chromatin movement, whether caused by chromatin condensation, DNA replication, or transcription, could result in inward facing forces but also in an effective increased pressure to the larger chromatin movement. In addition, the effective nuclear deformation and stress on the nuclear envelope will depend further on the local mechanical properties of the nuclear interior, the nuclear envelope, and the perinuclear cytoskeleton, which can all change dynamically. Together, these factors create a much more nuanced and multifaceted picture of nuclear mechanics that should be acknowledged by the authors.

The authors should provide some more background on Kabuki syndrome in the introduction. Furthermore, reducing the disease mechanism to be primarily based on altered mechanotransduction ("clinical manifestations recall an altered mechano-signaling during cell lineage commitment and tissue homeostasis") isn't sufficiently supported by the cited references and would raise questions why other mechanically sensitive tissues are not affected in this disease. While the authors interpretation that MLL4 loss of function altered cellular responses to mechanical stimuli, it seems very likely that loss of MLL4 has pleiotropic effects, including by altering transcriptional regulation independent of mechanosensing. Thus, rather than trying to explain all effects with a single simple hypothesis, the authors should present a more nuanced view to the reader.

UNIVERSITÀ DI TRENTO
Department of
Cellular, Computational and Integrative Biology - CIBIO

Trento, 30th of April 2026

Dear Editor,
please find below a point-by-point response to all the raised criticisms by the Referee #1. For clarity, we have highlighted changes in the revised manuscript related to the requested information's as red text.

Referee #1 comments

The authors have submitted a revised manuscript that addresses many of the previous reviewers' comments. Nonetheless, a number of questions and concerns remains. In addition, this reviewer, who was not involved in the previous review, also noticed a few additional items that should be addressed by the authors.

As the three reviewers previously stated, the manuscript would benefit from untangling further the mechanism by which MLL4 loss of function results in the various effects, particularly whether specific effects are directly downstream of altered condensation and chromatin mechanics, or potentially downstream of altered gene expression or other mechanotransduction pathways. The transcriptomic analysis and YAP/TAZ overexpression (Figures 6 and S6) indicate that at least some of the nuclear rupture effects occur downstream of other mechanotransduction pathways and not necessarily as a direct consequence of altered nuclear stiffness). The authors are commended for already including several new experiments in the revised manuscript that go towards answering this question. A more careful analysis of the temporal sequence and dynamics of effects, for example, in response to nuclear confinement or acute MLL4 depletion using the degron system, could substantially advance the current interpretation. Given that the authors have the tools available to disentangle the direct vs. indirect effects of MLL4 LoF, using for example at least some basic molecular profiling in combination with acute depletion of MLL4, and thus temporally separate primary from secondary effects, it is surprising that they have not taken advantage of these capabilities yet. Right now, the relatively long duration for some of the perturbations (30 min for nuclear confinement, 8(?) hours for the degron system), and 48 hours for substrate stiffness allows for many secondary effects to occur that make the interpretation of the results more challenging.

We thank the Reviewers for these criticisms, which are indeed relevant to better support the direct contribution of MLL4 in regulating nuclear mechanical responses through chromatin-mediated organization into condensates. To better dissect the direct effects of MLL4 on (i) chromatin compartmentalization, (ii) nuclear envelope (NE) integrity, and (iii) downstream cGAS/STING signaling, we increased the temporal resolution of our analyses by examining earlier time points following MLL4 degradation, specifically 2 and 4 hours after PROTAC3 treatment (data shown in Fig. 7I-K and Fig. EV6 O-V). The newly obtained results are in line with the originally presented data that were retrieved upon 8 h of PROTAC3 treatment. This time point was originally selected because it consistently produced a ~50% reduction in MLL4 protein abundance, closely mimicking *KMT2D* haploinsufficiency in our single-allele knock-in model.

In the newly obtained data, time-course results indicated that comparable levels of MLL4 depletion can be achieved as early as 2h after PROTAC3 treatment. We therefore repeated all the analyses shown at these earlier time points to resolve the direct contribution of MLL4 to chromatin

condensate dynamics and NE integrity. We found that 2h after MLL4 degradation H3K4me1 and H3K27ac levels decreased of about 50% (Fig. 7I and Fig. EV6Q-S), indicating its direct contribution to modulate this chromatin states, in line with what have been previously proposed[1-3]. In the same time frame, we measured a gradual weakening of transcriptional condensates clustering, while PcG condensates resulted in being augmented 8 hours after treatment (Fig. EV6T, U). Overall, this time-resolved analyses highlighted that while MLL4 directly contributes to determine the chromatin states and the assembly of transcriptional condensates, the measured redistribution and organization of PcG proteins into condensates may result as an indirect compensatory mechanism to preserve chromatin organization. Nevertheless, the acute MLL4 degradation diminished Lamin A/C level (Fig. EV6V), resulting in the activation of cGAS-STING pathway that enhanced the sensitivity of $MLL4^{HaloTag}$ MSCs to mechanical stress, resulting in a time-dependent increase in cell death (Fig. 7J, K).

With respect to confinement experiments, we acknowledge the limitations of our current static confiner. To improve temporal resolution, we implemented an orthogonal approach in which cells are confined in suspension, following the protocol that was originally designed by the Piel team to determine the contribution of the nucleus to cell mechano-response[4]. This strategy enables us to uncouple compressive forces from cytoskeleton-mediated forces, which are present when confinement is applied to adherent cells. Using this approach, we observed a time-dependent response of MLL4 PrLD to confinement, with clustering emerging as early as 5 minutes after the application of mechanical load and reaching a maximum within 30 minutes (Fig. 3F–H). Notably, this response scaled with the level of applied mechanical stress, as 3 μm confinement induced a marked increase in both the number and size of MLL4 PrLD condensates. Importantly, this dynamic response was strongly attenuated in the $MLL4^{Q4092X}$ MSCs chromatin context and was dependent on the polyQ stretch of the MLL4 PrLD (Fig. 3F–I). Overall, by improving temporal resolution and distinguishing nuclear-intrinsic responses from ECM-mediated mechanical inputs, these experiments further support a direct role for the MLL4 in regulating the dynamic assembly of chromatin condensates.

In their response to reviewer #3, the authors state that "we demonstrated that nuclear integrity relies on the balance between transcriptional condensates and PcG condensates". In that case, would the authors expect that any shift in the balance between transcriptional condensates and PcG would result in loss of nuclear integrity? This should be tested experimentally.

This is an important conceptual question that we agree merits further discussion. Although many cofactors have been implicated in transcriptional condensate assembly via IDR-driven multivalent interactions, we currently lack definitive evidence identifying which specific chromatin factors directly tune the biophysical properties of these condensates. Moreover, emerging work suggests that many transcriptional cofactors share molecular features including PolyQ-rich IDRs potentially favoring interactions with RNA Pol II[5], but whether this “molecular grammar” regulates condensate assembly in a manner that reciprocally affects PcG condensates remains unresolved. Experimentally addressing it, however, would require perturbation of an extensive numerous chromatin cofactors and/or their intrinsically disordered regions (IDRs) through point mutations. Indeed, we have shown that Q-to-A substitutions within MLL4 PrLD, which is characterized by a PolyQ stretch, are sufficient to abrogate MLL4 mechano-responsiveness. We interpreted this result considering the high propensity of PolyQ stretch to contribute to $\boldsymbol{\beta}$-rich configurations compare to Alanine, thus weakening the conformational transition in response to mechanical load. Whether a similar mechanism governs the assembly of other chromatin factors characterized by PolyQ-rich IDRs into transcriptional condensates remains largely unclear. Even if shared, it cannot be assumed that these factors would equivalently contribute to

counterbalancing PcG condensates. Moreover, it should also be evaluated how such mechano-mediated conformational transitions may influence interactions with the CTD of RNA Pol II and whether this contributes to tuning the assembly of PcG condensates. While highly interesting, rigorously testing this hypothesis goes beyond the scope of the present study, at this stage. We have, however, explicitly discussed its conceptual relevance in the revised manuscript (pages 19 and 20 of the revised manuscript).

I do share the concerns of Reviewer #3 about the need to validate Brillouin microscopy measurements of nuclear stiffness using orthogonal approaches such as AFM, nanoindentation, nuclear stretching, or micropipette aspiration. These techniques have all been used successfully to demonstrate changes in nuclear stiffness in response to altered chromatin organization. While Brillouin microscopy does offer the advantage of spatial resolution and contactless measurements, it is also more prone to measurements being affected by confounding factors such as water content, cell morphology, etc. These factors further complicate the interpretation of Brillouin phase shift measurements and translating them into corresponding changes in material properties. Since the authors report Brillouin shift measurements averaged across the entire nucleus and do not try to determine subnuclear differences in nuclear mechanics, their results should also be detectable by bulk nuclear stiffness measurements using other technologies. The in vitro experiments using MSCs should be well suited for such studies.

We acknowledge that each technique used to measure nuclear stiffness carries inherent limitations. We selected Brillouin microscopy because it uniquely provides spatially resolved, contact-free measurements that allow us to distinguish the local contribution of chromatin organization to nuclear mechanics. By contrast, most alternative approaches including AFM, micropipette aspiration, and nanoindentation measure integrated mechanical responses of the whole nucleus and cannot discriminate among distinct nuclear components.

That said, we agree that orthogonal measurements would strengthen our conclusions regarding the contribution of MLL4 to chromatin biophysical properties and, more broadly, to nuclear mechanics. We therefore complemented the Brillouin microscopy data with two additional approaches:

a. AFM-based measurements of nuclear stiffness. We performed Atomic Force Microscopy on MSCs grown on soft and stiff matrix by placing the cantilever probe tip directly over the nucleus to then determine the elastic module *E*. These measurements were consistent with previously reported AFM-based nuclear stiffness values and, importantly, corroborated our Brillouin microscopy data. Specifically, we observed an increase in nuclear stiffness in MLL4 LoF MSCs grown on stiff matrices, which was rescued by restoring PcG condensate balance through expression of the H3.3K27M dominant-negative mutant (Fig. Appendix 3).
b. Micro-rheology measurements of nuclear viscosity by probing nucGEM motions within the nucleoplasm[6]. This method captures mesoscale properties of nucleoplasm, which are affected by changes in chromatin compartmentalization and/or mechano-osmotic signal[6-8]. Indeed, nucGEM micro-rheology has recently been adopted to detect mesoscale chromatin organization changes[7] and to corroborate variations in nuclear mechanical properties measured by AFM[8]. By determining nucGEM means square displacement (MSD) from which we retrieved the the diffusion coefficient *D* and the anomalous diffusive exponent α (Fig. 1G-K), we found that increased ECM stiffness was associated with a reduced particle motion, reflecting an increase in viscosity and macromolecular crowding, in line with what have been previously shown[8]. When comparing the contribution of MLL4 to the nucleoplasm mesoscale properties, we found that

its haploinsufficiency correlated with a modest increase in α values that was partially rescued by the concomitant inhibition of PcG activity (H3.3K27M condition), suggesting a direct contribution of chromatin compartmentalization to the nuclear biophysical properties.

Overall, these orthogonal measurements are consistent with the Brillouin microscopy data and support the conclusion that MLL4 contributes to the material properties of the nucleus and its mechanical responsiveness to ECM-derived forces.

In their characterization in Figure 1 and S1, the authors provide information on BRD4, BMI, and PcG cluster number, size, and total fluorescence intensity, and state that the transcript number of MLL4 is unchanged in the MLL4 LoF cells. Since transcript number doesn't always correlate with protein levels, the authors should also confirm MLL4 protein levels at the different substrate conditions by Western blot analysis.

We have performed the requested control by quantifying MLL4 protein abundance by WB, which remained unchanged across substrate stiffness conditions, corroborating the IF measurements and the transcripts levels. We included these data as supplementary material (Fig. Appendix 1).

The graph for the results of the confinement experiments shown in Fig. 3G, H is missing a crucial comparison, the data for the MLL4 LoF cells under 3 um confinement, given that only 3um confinement elicited an increase in MLL4 condensates. If the authors experienced increased rates of cell death under those conditions, they could still try to analyze the live MLL4 LoF cells under the 3um confinement conditions. In addition, the authors should consider including short timepoints, as presumably the confinement itself is achieved within minutes (or faster), and condensation should occur on similar or shorter time scales. This would enable them to further discern whether the confinement induced condensation is primarily driven by changes in nuclear geometry-and likely volume-or potentially downstream of other mechanoresponsive factors. In addition, the authors should test whether other proteins such as BMI form increased or increased condensates under confinement.

As anticipated by the Reviewer, MLL4 LoF cells grown under adherent conditions exhibit substantial cell death upon 3 μm confinement, which introduces major confounding factors when attempting to quantify condensate responses. For this reason, we initially omitted these data, as cells are subjected to combined compressive and tensile forces that are not compatible with survival in the MLL4 LoF condition.

T To address this limitation experimentally, we adopted an alternative approach in which cells were confined while maintained in suspension, allowing us to preserve cell viability and reliably quantify chromatin condensates[9]. Importantly, this strategy also enabled improved temporal resolution. Under these conditions, we observed that MLL4 PrLD condensates are rapidly induced, with clustering detectable as early as 5 minutes after confinement, and that this response is attenuated in the MLL4 LoF condition (Fig. 3F–H). Notably, the increase in condensate size scaled with the level of applied mechanical load and was maintained at later time points (30 minutes). These results, combined with the measurements of changes in nuclear area, indicate that coacervation of MLL4 into condensates occur in the same time window of nuclear mechanical response to cell confinement, with MLL4 LoF affecting mechano-responsiveness.

Concerning the potential mechano-response of other chromatin factors harboring IDRs and belonging to chromatin condensates, we have addressed this point in the previously revised manuscript (data now shown in Fig. EV2 I, J). Specifically, we tested the behavior of BMI1 and MED1 using the same

optoIDR system through which we assessed MLL4 PrLD responsiveness in response to cell confinement. Under confinement conditions, neither protein showed an increase in condensate formation, suggesting that mechano-responsiveness is not a general property of chromatin condensates, but may depend on specific features of their IDRs. Considering this observation, we have expanded the Discussion (pages 19–20) to comment on how IDR composition and sequence features may contribute to the selective mechano-responsiveness of chromatin regulators.

In the confinement experiments shown in Suppl. Fig. S3F, it appears that the cell density of the MLL3 LoF cells in the 3um confinement condition is substantially lower than in the other conditions (only four nuclei are visible in the field of view, compared to ~8 nuclei/FOV for the other conditions), which could affect the extent of confinement and survival. The authors should comment on this discrepancy and confirm whether experiments were carried out at similar cell densities.

We confirmed that the experiments were performed using the same cell density. As pointed out by the Reviewer, the reduced number of cells/FOV in the MLL4 LoF 3µm confinement condition reflects an increased cell death. Said that, we addressed this potential confounding factor by repeating all the measurements on cells in suspension, as previously described (data shown in Fig. 3F-H). We are confident that this new set of results properly address the raised concern providing valuable data related to the MLLl4 mechano-response to confinement.

In the confinement experiments shown in Suppl. Fig. S3H, I, it seems very surprising that the cross-sectional area of the nuclei subject to 3um confinement is substantially smaller than that of the unconfined nuclei, since typically confinement results in an increase in nuclear cross-sectional area. The authors should explain this unexpected finding.

We thank the Reviewer for highlighting this point, which we have carefully re-examined using multiple approaches. Upon revisiting the images, we found that the representative examples shown did not accurately reflect the overall changes in nuclear morphology upon confinement. In our datasets, we typically observe a modest increase in nuclear cross-sectional area after 90 minutes of 3 µm confinement, consistent with previous reports. Notably, by comparing the changes in cross-sectional nuclear area upon confinement of MSCs grown in adhesion or in suspension, we found a remarkable difference with the latter condition providing a heigh-dependent linear increase in nuclear area, already measurable five minutes after providing the mechanical stimulus (shown in Fig. 3F). This observation indicates that measuring the effects of confinement on cells in suspension represents a valuable experimental condition as it permits to couple nuclear mechanical response with the dynamic assembly of chromatin condensates[9].

LINC complex disruption using dominant negative nesprin or SUN domain constructs is highly dependent on the expression levels. For the experiments using the C1-KASH construct, the authors should thus confirm successful LINC complex disruption by demonstrating displacement of endogenous nesprins from the nuclear envelope.

We agree with the Reviewer that the effect of the dominant negative GFP–Nesprin-1/2–KASH construct depends on its expression level. To address this point, we assessed the efficacy of the C1-KASH construct in disrupting LINC complex function by monitoring changes in actin organization using Phalloidin staining (Fig. EV1F). This readout provides a functional measure of impaired cytoskeleton–

nucleus coupling and is complementary to the direct assessment of nesprin displacement. Importantly, all analyses related to LINC complex disruption were restricted to cells exhibiting a clear reduction in actin polymerization reflected by a decrease in the cell area, thereby ensuring that only cells with effective perturbation of LINC function were included.

While the molecular dynamics simulations are informative, in the current form, the description in the Results section lacks sufficient detail for a reader to interpret the results. The authors should comment on how the increased pressure was modeled in the simulation and how the applied pressure (1, 1.5, or 1.75 atm) compares to experimental conditions. Otherwise, it is difficult to discern whether the applied settings correspond to physiological conditions.

We agree that additional detail is required for readers to fully interpret our simulations. We have therefore revised the Results section (pages 11–12) to more clearly describe how pressure was implemented in the molecular dynamics framework. We now clarify that the application of increasing external pressure (1.0 to 1.75 atm) is used as a computational parameter to probe the sensitivity of the polyQ tract to changes in its physical environment, thereby mimicking the effects of mechanical constraints acting on nuclear components such as chromatin and associated macromolecules. We also emphasize that this range of pressure does not aim to directly recapitulate physiological forces, but rather to explore how small perturbations in the system can influence conformational transitions. We believe that this expanded description better clarifies the rationale underlying the simulation design, whose predictions are further supported by our experimental validation (Fig. 4G).

For the measurements of lamin A/C levels (Fig. 5), the authors should confirm altered lamin A/C levels by western blot analysis, given that antibody binding to lamin A/C can depend vary substantially based on mechanical stress (PMID 26301768) and cell density (PMID 36809122). Furthermore, this would also enable the authors do distinguish between the lamin A and C isoforms. For all experiments, the authors should further include measurements of lamin B levels, which are less sensitive to substrate stiffness (PMID 23990565), as a control.

We performed the requested control by monitoring the Lamin A/C protein abundance by WB. The retrieved results (shown in Fig. Appendix 4) confirmed the pattern detected by IF, with an increment of both Lamin A and C levels in response to the increased substrate stiffness, which was weakened in MLL4 LoF conditions.

In the description of the experiments with the MLL4 HaloPROTRAC3 system, the authors should include the exact treatment duration, instead of just referring to "by shortly treating" in the text. Right now, neither the main text or the figure legends include this important information about the treatment duration, which is crucial for the interpretation. The Method section indicates "for 8/24 hours", which does not provide sufficient clarify either.

We apologize for the lack of clarity. We have revised all figure legends of Fig. 7 and EV6 and the main text in which we specified the extent of PROTAC3 treatment.

The authors interpretation that "maintaining nuclear integrity requires a proper balancing between inward, nuclear-generated and outward, ECM-mediated mechanical forces." (Introduction) and "applied outward forces are balanced by chromatin-mediated inward forces to preserve nuclear

morphology and genome integrity" (Response to reviewers) is too simplistic and likely inaccurate. In mesenchymal cells, cytoskeletal forces likely apply both compressive forces (via apical stress fibers and the 'actin cap') and tensile forces to the nucleus. Intranuclear chromatin movement, whether caused by chromatin condensation, DNA replication, or transcription, could result in inward facing forces but also in an effective increased pressure to the larger chromatin movement. In addition, the effective nuclear deformation and stress on the nuclear envelope will depend further on the local mechanical properties of the nuclear interior, the nuclear envelope, and the perinuclear cytoskeleton, which can all change dynamically. Together, these factors create a much more nuanced and multifaceted picture of nuclear mechanics that should be acknowledged by the authors.

We thank the Reviewer for raising this biologically relevant point related to the complexity of mechanical signals controlling nuclear mechanical properties. As the Reviewer correctly pointed out, nuclear mechanics reflects a complex integration of cytoskeletal tensile and compressive forces, intranuclear chromatin dynamics, nuclear envelope properties, and perinuclear cytoskeletal organization. Our simplified description was intended to maintain accessibility for a broad audience, but we agree that a more comprehensive and extended paragraph on this topic would facilitate the understanding of the biological relevance of dissecting the molecular mechanisms governing the multifaceted and dynamic nature of nuclear force balance. To promptly address the raised point, we have revised the manuscript as follows:

Introduction section (page 3): we have modified the original statement to avoid an oversimplified "force balance" model and instead emphasize that nuclear integrity results from the integration of mechanical inputs from both the ECM/cytoskeleton and intranuclear components such as chromatin.

Discussion section (page 19), we have clarified that chromatin condensates should be viewed as one component within a broader mechanical framework. We now explicitly acknowledge that cytoskeletal forces (both tensile and compressive) and intranuclear processes such as chromatin dynamics contribute to shaping nuclear mechanics. This clarification has been incorporated without altering the overall interpretation of our data. We believe these revisions better position our findings within the current understanding of nuclear mechanobiology while maintaining the focus of the study.

The authors should provide some more background on Kabuki syndrome in the introduction. Furthermore, reducing the disease mechanism to be primarily based on altered mechanotransduction ("clinical manifestations recall an altered mechano-signaling during cell lineage commitment and tissue homeostasis") isn't sufficiently supported by the cited references and would raise questions why other mechanically sensitive tissues are not affected in this disease. While the authors interpretation that MLL4 loss of function altered cellular responses to mechanical stimuli, it seems very likely that loss of MLL4 has pleiotropic effects, including by altering transcriptional regulation independent of mechanosensing. Thus, rather than trying to explain all effects with a single simple hypothesis, the authors should present a more nuanced view to the reader.

We thank the Reviewer for this comment related to the potential link between KS phenotypes and mechano-response. Our intention was to highlight that MLL4 LoF likely affects both genetic and non-genetic chromatin functions, but we agreed that the original statement was providing a biased view on the potential underlying mechanisms. We have extended the introduction section to provide more nuanced and balanced background on the clinical manifestations associated with Kabuki Syndrome (page 4 of the revised manuscript). Additionally, we have removed the original statement in which we

linked the clinical manifestation to an altered mechano-signalling to avoid implying that altered mechanotransduction alone accounts for the full clinical spectrum.

Cited literature

1. Hu, D. *et al.* The MLL3/MLL4 branches of the COMPASS family function as major histone H3K4 monomethylases at enhancers. *Mol Cell Biol* **33**, 4745–4754 (2013).
2. Xie, G. *et al.* MLL3/MLL4 methyltransferase activities control early embryonic development and embryonic stem cell differentiation in a lineage-selective manner. *Nat Genet* **55,** 693–705 (2023).
3. Wang, S. P. *et al.* A UTX-MLL4-p300 Transcriptional Regulatory Network Coordinately Shapes Active Enhancer Landscapes for Eliciting Transcription. *Mol Cell* **67**, 308–321 e306 (2017).
4. Lomakin, A. J. *et al.* The nucleus acts as a ruler tailoring cell responses to spatial constraints. *Science* **370** (2020).
5. Ruff, K. M. *et al.* Molecular grammars of predicted intrinsically disordered regions that span the human proteome. *Cell* **189,** 323–342 e317 (2026).
6. Szoradi, T. *et al*. Genetically encoded multimeric (GEM) nanoparticles probe the biophysical properties of the nucleus. *Cell Press* **121**, 496a (2022).
7. Xia, J., Zhao, J. Z., Strom, A. R. & Brangwynne, C. P. Chromatin heterogeneity modulates nuclear condensate dynamics and phase behavior. *Nat Commun* **16,** 6406 (2025).
8. McCreery, K. P. *et al*. Mechano-osmotic signals control chromatin state and fate transitions in pluripotent stem cells. *Nat Cell Biol* **27,** 1757–1770 (2025).
9. Lomakin, A. J. *et al.* The nucleus acts as a ruler tailoring cell responses to spatial constraints. *Science* **370** (2020).

Dear Prof. Zippo,

Thank you for submitting a revised version of your manuscript. Your study has now been re-evaluated by the arbitrating referee, who finds that their previous concerns have been addressed and now recommends publication pending a few minor textual revisions, which we also consider reasonable and helpful.

Apart from these points, there remain only a few mainly editorial issues that need to be addressed before I can formally accept the manuscript for publication:

- FUNDING INFO: missing info in eJP: Short-EMBO Fellowship (#8386); Fabrizio A. Pennacchio by AIRC fellowship #23966; EIC-2022-PathfinderOpen (ivBM-4PAP, n. 101098989); Project "National Center for Gene Therapy and Drugs based on RNA Technology" (CN00000041) financed by NextGeneration EU PNRR MUR-M4C2-Action 1.4-Call "Potenziamento strutture di ricerca e creazione di "campioni nazionali di R&S" (CUP J33C22001130001); the Italian Association for Cancer Research (AIRC), IG# 22911; by the European Union under NextGenerationEU (PRIN 2022 #2022H79275).
REFERENCE FORMAT: not ok, should be alphabetical with 10 authors + et al.
DATA AVAILABILITY SECTION: in, but "Materials availability" and "Data and code availability" should be compiled in one section named "Data Availability"

- As we are switching from a free-text author contribution statement towards a more formal statement based on Contributor Role Taxonomy (CRediT) terms, please remove the present Author Contribution section and instead specify each author's contribution(s) directly in the Author Information page of our submission system during upload of the final manuscript. See https://casrai.org/credit/ for more information.

- FIGURE CALLOUTS: all callouts should be listed sequentially; missing callouts for Fig. 1K and 3J

- EV figures should also be upldd as individual, high-resolution Figure files with legends placed in ms below the main figure legends

- APPENDIX 1 FILE WITH ToC: Appendix file needs to be in PDF format; title page should contain "Appendix for + ms title" (author and affiliation list not necessary) and ToC with the page numbers for the listed items; nomenclature correct in Appendix, but nomenclature should be Appendix Figure S1-S4 throughout ms as callouts

- SOURCE DATA: missing SD files for Fig. 4B-F and 5E; Please provide the Source data in a scheme one figure/folder and then uploaded as .zip files. E.g. all the Source data files for figure 1 need to be saved in a single folder and this needs to be zipped and then uploaded as "SD figure 1.zip" file. For EV and/or appendix figures, ZIP together all source data.

- Please provide suggestions for a short 'blurb' text prefacing and summing up the conceptual aspect of the study in two sentences (max. 250 characters), followed by 3-5 one-sentence 'bullet points' with brief factual statements of key results of the paper; they will form the basis of an editor-written 'Synopsis' accompanying the online version of the article. Please also provide an altered synopsis image, making sure that the aspect ratio conforms to our website's format - it should be exactly 550 pixels wide and 300 pixels high.

- Figure Legends (main + EV):

1. Please note that the exact p values are not provided in the legends of figures 1A, B, C, D, F, J; 2C, D, E, G; 3C, D, F, G, H, J; 5A, C, D, F; 6A, G, I; 7A, B, C, D, F, G, I, J, K; EV1 A, B, C, D, E, F, G, H, I, J, K, L, M, N, O; EV2 B, C, D, E, G, H; EV4 A, B, C, D, E, F, G, H, J, K, L, M; EV5 A-C, E-H, J, L; EV6 B-E, G-M, N, Q, R, S, T, U, V.

2. Please note that the box plots need to be defined in terms of minima, maxima, centre, bounds of box and whiskers, and percentile in the legend of figure 7H

3. Please note that information related to n is missing in the legend of figure 7H"

4. Please note that the dashed borders are not defined in the legend of figures 3B, J; 6E, H, J; EV5 I, K. This needs to be rectified.

Notes:
- Plese rename the movie files to Movie EV1-EV15 with the corresponding callouts, and the legends should be removed from EV figure file and zipped with each movie file.

- Sections need to be named and the order should be corrected: Title page - Abstract - Introduction - Results - Discussion - Methods - Data Availability - Acknowledgements - Disclosure and Competing Interests Statement - References - Figure Legends

- Table(s) - Expanded View Figure Legends. "Declaration of generative AI and AI-assisted technologies in the writing process" section should be placed after the DAS section.

Please let me know if you have any questions regarding any of these points. You can use the link below to upload the revised files.
Thank you again for giving us the chance to consider your manuscript for The EMBO Journal. I look forward to receiving the final version.

With best regards,

Cornelius Schneider

____
Cornelius Schneider, PhD
Editor | The EMBO Journal
c.schneider@embojournal.org

Please refer to our figure preparation guideline in order to ensure proper formatting and readability in print as well as on screen:

https://link.springer.com/journal/44318/submission-guidelines#cms-Figure-and-data-presentation

Instructions for preparing your revised manuscript:

Read our guidance for manuscript revisions and related editorial policies: https://link.springer.com/journal/44318/submission-guidelines#cms-Revised-submissions

Please also check that the title and abstract of the manuscript are brief, yet explicit, even to non-specialists.

When assembling figures, please refer to our figure preparation guideline in order to ensure proper formatting and readability in print as well as on screen:
https://media.springernature.com/original/springer-cms/rest/v1/content/27825798/data/v1

IMPORTANT: When you send the revision we will require

- a point-by-point response to the referees' comments, with a detailed description of the changes made (as a word file).
- a word file of the manuscript text.
- individual production quality figure files (one file per figure)
- a complete author checklist
- Expanded View files (replacing Supplementary Information)
- a Reagents and Tools Table as part of the Methods section

Please remember: Digital image enhancement is acceptable practice, as long as it accurately represents the original data and conforms to community standards. If a figure has been subjected to significant electronic manipulation, this must be noted in the figure legend or in the 'Methods' section. The editors reserve the right to request original versions of figures and the original images that were used to assemble the figure.

We realize that it is difficult to revise to a specific deadline. In the interest of protecting the conceptual advance provided by the work, we recommend a revision within 3 months (23rd Sep 2026). Please discuss the revision progress ahead of this time with the editor if you require more time to complete the revisions. Use the link below to submit your revision:

https://emboj.msubmit.net/cgi-bin/main.plex

Referee #1:

The authors have submitted a revised manuscript with substantial new experimental data to support their findings. These data, along with changes made to the text, address the previous concerns. There are just some minor comments the authors should address:

Page 4, second paragraph. Please double check the following sentence, which appears to have an extraneous quotation mark and some other issues, likely resulting from text edits and cutting and pasting text segments: "however, emerging evidence suggests that defects in cellular responses to mechanical cues may also contribute to aspects of the disease, particularly in the context of tissue development and homeostasis." whose clinical manifestations recall an altered mechano-signaling during cell lineage commitment and tissue homeostasis35-37."

Page 6/7. The newly inserted section with the LINC complex disruption validation is out of place here and factually not accurate, since the referenced panels (Fig. EV1F, G) only show the effect of LINC complex disruption on cell spreading, without including any data supporting the statement that these experiments corroborate the mechano-responsiveness of the transcriptional condensates. The text should be revised accordingly and moved to where the LINC complex disruption is used for functional studies.

Page 8, top paragraph. The authors write that "To determine whether the measured MLL4-dependent increase in nuclear stiffening affected the nucleoplasm viscosity". This would imply that the increased nuclear stiffening drives changes in nucleoplasm viscosity, which is unlikely be the case. Most likely, both stiffness and viscosity change based on differences in chromatin organization. The author should thus replace "affected" with "associated with".

Appendix Figure S1. The graph on the right is missing labels to indicate what the different colors of the bars represent. Presumably they indicate soft and stiff substrates?

Appendix Figure S4. The authors have added a Western blot for lamin A/C, but do not provide any quantification, and it is not clear if their conclusions are drawn based on a single western blot or if only a representative blot is shown here. The authors should provide quantification and statistical analysis regarding changes in the expression of lamin A and C. Note that the figure legend for panel B contains several typos and inconsistencies in the capitalization of words.

UNIVERSITÀ DI TRENTO
Department of
Cellular, Computational and Integrative Biology - CIBIO

Trento, 29th of June 2026

Dear Editor,
please find below a point-by-point response to all the remaining textual corrections raised by the Referee #1. For clarity, we have highlighted changes in the revised manuscript related to the requested information's as red text.

Referee #1 comments

Referee #1:

The authors have submitted a revised manuscript with substantial new experimental data to support their findings. These data, along with changes made to the text, address the previous concerns. There are just some minor comments the authors should address:

We pleased that the Reviewer acknowledged that the new set of experimental data and textual clarifications fully addressed the previously raised criticisms.

Page 4, second paragraph. Please double check the following sentence, which appears to have an extraneous quotation mark and some other issues, likely resulting from text edits and cutting and pasting text segments: "however, emerging evidence suggests that defects in cellular responses to mechanical cues may also contribute to aspects of the disease, particularly in the context of tissue development and homeostasis." whose clinical manifestations recall an altered mechano-signaling during cell lineage commitment and tissue homeostasis35-37."

We thank the Reviewer for this note, and we have edited the text as follows: "However, emerging evidence suggests that defects in cellular responses to mechanical cues may also contribute to aspects of the disease, particularly in the context of development and tissue homeostasis".

Page 6/7. The newly inserted section with the LINC complex disruption validation is out of place here and factually not accurate, since the referenced panels (Fig. EV1F, G) only show the effect of LINC complex disruption on cell spreading, without including any data supporting the statement that these experiments corroborate the mechano-responsiveness of the transcriptional condensates. The text should be revised accordingly and moved to where the LINC complex disruption is used for functional studies.

We thank the Reviewer for highlighting this point, although we do not agree with this comment as we showed the direct effect of the overexpression of the dominant negative GFP–Nesprin-1/2–KASH protein on the numerosity of transcriptional condensates, as measured by BRD4 immunostaining (sown in Fig. EV1G). As correctly mentioned by the Reviewer, we also provided representative images and quantification of the perturbation of cell spreading upon LINC complex disruption (Fig. EV1F) to properly address the previous Reviewer's request. For these reasons we think that the text properly described the retrieved results and it is not misplaced, as suggested by the Reviewer.

Page 8, top paragraph. The authors write that "To determine whether the measured MLL4- dependent increase in nuclear stiffening affected the nucleoplasm viscosity". This would imply that the increased nuclear stiffening drives changes in nucleoplasm viscosity, which is unlikely be the case. Most likely, both stiffness and viscosity change based on differences in chromatin organization. The author should thus replace "affected" with "associated with".

We thank the Reviewer for highlighting this point, and we edited the text, accordantly.

Appendix Figure S1. The graph on the right is missing labels to indicate what the different colors of the bars represent. Presumably they indicate soft and stiff substrates?

We thank the Reviewer for highlighting this point and we edited the Appendix Figure S1 by including the missing labels in the bar plot. We also noticed a similar mislabelling of the scatter plot shown in Appendix Figure S3 and we edited the figure accordantly.

Appendix Figure S4. The authors have added a Western blot for lamin A/C, but do not provide any quantification, and it is not clear if their conclusions are drawn based on a single western blot or if only a representative blot is shown here. The authors should provide quantification and statistical analysis regarding changes in the expression of lamin A and C. Note that the figure legend for panel B contains several typos and inconsistencies in the capitalization of words.

To address the Reviewer's criticism, in the revised manuscript we included the WB for Lamin A/C , which showed that in response to ECM stiffness both protein isoforms are equally affected in the MLL4 LoF condition. In addition, with this assay we corroborated the quantitative measurements of Lamin A/C perturbations that was originally retrieved by IF detection of Lamin A/C (Fig. 5A). Given that this assay was performed to confirm the previous data, we did not perform it in independent biological replicates by which we did not provide statistical measurements of the relative changes in Lamin A/C changes. Concerning the figure legend of Panel B, we here described the quantification of Lamin B intensity performed by IF. We clarified it in the main text in which when we refer to Appendix Figure 4 we stated "We found that while in the wild-type cells Lamin A/C abundance increased in response to the augmented stiffening of the ECM, the *KMT2D* haploinsufficiency resulted in reduced responsiveness to the same mechanical load, while Lamin B levels were unperturbed (Fig. 5A and Appendix Figure S1 and S4)." I hope this clarification improved the readiness of the manuscript related to Lamin A/C and B level changes in response to both mechanical load and MLL4 LoF condition.

Editorial comments

- FUNDING INFO: missing info in eJP: Short-EMBO Fellowship (#8386); Fabrizio A. Pennacchio by AIRC fellowship #23966; EIC-2022-PathfinderOpen (ivBM-4PAP, n. 101098989); Project "National Center for Gene Therapy and Drugs based on RNA Technology" (CN00000041) financed by NextGeneration EU PNRR MUR-M4C2-Action 1.4-Call "Potenziamento strutture di ricerca e creazione di "campioni nazionali di R&S" (CUP J33C22001130001); the Italian Association for Cancer Research (AIRC), IG# 22911; by the European Union under NextGenerationEU (PRIN 2022 #2022H79275).

We included the funding information, as requested.

- REFERENCE FORMAT: not ok, should be alphabetical with 10 authors + et al.

We edited the references and ordered alphabetically in the revised manuscript, as requested.

DATA AVAILABILITY SECTION: in, but "Materials availability" and "Data and code availability" should be compiled in one section named "Data Availability"

We edited the DATA AVAILABILITY section, as indicated.

- As we are switching from a free-text author contribution statement towards a more formal statement based on Contributor Role Taxonomy (CRediT) terms, please remove the present Author Contribution section and instead specify each author's contribution(s) directly in the Author Information page of our submission system during upload of the final manuscript. See https://casrai.org/credit/ for more information.

We removed the present Author Contribution section from the main text and instead we specified each author contribution in the relative information page, as requested.

- FIGURE CALLOUTS: all callouts should be listed sequentially; missing callouts for Fig. 1K and 3J

Thank you for highlighting this point. We have corrected the Fig. 1 (in which we included the letter "k" for labeling the last panel) and the main text, accordantly. Regarding the missing callouts for Fig. 3J, we edited the main text in which the panel was erroneously indicated as Fig. 3I.

- EV figures should also be upldd as individual, high-resolution Figure files with legends placed in ms below the main figure legends

We have uploaded the EV figures as individual, high-resolution figure files and inserted the corresponding figure legend in the main manuscript file, below the main figure legends, as requested.

- APPENDIX 1 FILE WITH ToC: Appendix file needs to be in PDF format; title page should contain "Appendix for + ms title" (author and affiliation list not necessary) and ToC with the page numbers for the listed items; nomenclature correct in Appendix, but nomenclature should be Appendix Figure S1-S4 throughout ms as callouts

We have uploaded a single pdf file for Appendix Figures and the related ToC, as requested. We also properly corrected the nomenclature and its citation in the main manuscript, as suggested.

- SOURCE DATA: missing SD files for Fig. 4B-F and 5E; Please provide the Source data in a scheme one figure/folder and then uploaded as .zip files. E.g. all the Source data files for figure 1 need to be saved in a single folder and this needs to be zipped and then uploaded as "SD figure 1.zip" file. For EV and/or appendix figures, ZIP together all source data.

We have uploaded all the SD files as .zip files. However, we had to provide separate folders for figures exceeding the300MB limit, as requested. Concerning the 4B-F missing SD, this is due to the fact that the retrieved results represent computational modelling by molecular dynamics simulations, which do not rely on experimental measurements (raw data) but mathematical data after computing the conformation of the studied proteins. Given the dimensions of the generated mathematical data and their intrinsic nature, we can not include them as listed in the updates source Data checklist. We have discussed with the EMBO Editorial office that agreed about this solution.

- Please provide suggestions for a short 'blurb' text prefacing and summing up the conceptual aspect of the study in two sentences (max. 250 characters), followed by 3-5 one-sentence 'bullet points' with brief factual statements of key results of the paper; they will form the basis of an editor-written 'Synopsis' accompanying the online version of the article. Please also provide an altered synopsis image, making sure that the aspect ratio conforms to our website's format - it should be exactly 550 pixels wide and 300 pixels high.

We propose the following blurb text:

Besides contributing to genome function, chromatin condensates are active components of the nuclear mechano-response. MLL4 functions as a chromatin mechano-sensor that preserves nuclear integrity and restrains cGAS-STING signalling.

Bullet points:

- Mechanical loading shifts the dynamic balance between transcriptional and Polycomb condensates to tune nuclear mechanical properties.
- MLL4 assembles into mechano-responsive condensates through its Prion-like domain, acting as a chromatin mechano-sensor.
- MLL4-dependent condensate dynamics preserve nuclear integrity in response to mechanical stress.
- MLL4 haploinsufficiency in Kabuki Syndrome compromises the nuclear adaptation to mechanical stress, increasing nuclear fragility.
- Persistent nuclear envelope rupture activates cGAS-STING signalling, linking defective chromatin mechano-responsiveness to Kabuki syndrome pathology

Weh have uploaded a graphical abstract, following your indications

- Figure Legends (main + EV):

1. Please note that the exact p values are not provided in the legends of figures 1A, B, C, D, F, J; 2C, D, E, G; 3C, D, F, G, H, J; 5A, C, D, F; 6A, G, I; 7A, B, C, D, F, G, I, J, K; EV1 A, B, C, D, E, F, G, H, I, J, K, L, M, N, O; EV2 B, C, D, E, G, H; EV4 A, B, C, D, E, F, G, H, J, K, L, M; EV5 A-C, E-H, J, L; EV6 B-E, G-M, N, Q, R, S, T, U, V.

We fully agree on the importance of reporting exact p-values and ensuring full statistical transparency. However, given the very high number of statistical comparisons across the indicated figures, including all exact p-values within the figure legends would result in extremely lengthy and complex text, which we feel would significantly compromise their readability and clarity. To address this concern, we included significance symbols in the figure legends following the standard GraphPad Prism notation: * = $p < 0.05$, ** = $p < 0.01$, *** = $p < 0.001$, **** = $p < 0.0001$, ns = not significant ($p \geq 0.05$), as agreed with the Editor.

2. Please note that the box plots need to be defined in terms of minima, maxima, centre, bounds of box and whiskers, and percentile in the legend of figure 7H

We have properly defined the box plots terms in the related figure legend referring to Fig. 7H, as suggested.

3. Please note that information related to n is missing in the legend of figure 7H"

We have properly included the information about the number of replicates as “n” in the figure legend referring to Fig. 7H, as suggested.

4. Please note that the dashed borders are not defined in the legend of figures 3B, J; 6E, H, J; EV5 I, K. This needs to be rectified.

We have properly defined the dashed boarders in the mentioned figure legends.

Notes:
- Plese rename the movie files to Movie EV1-EV15 with the corresponding callouts, and the legends should be removed from EV figure file and zipped with each movie file.

We have renamed the movie files, as requested and we also included the corresponding legend in each movie file, as requested.

- Sections need to be named and the order should be corrected: Title page - Abstract - Introduction - Results - Discussion - Methods - Data Availability - Acknowledgements - Disclosure and Competing Interests Statement - References - Figure Legends - Table(s) - Expanded View Figure Legends. "Declaration of generative AI and AI-assisted technologies in the writing process" section should be placed after the DAS section.

We have organized the main manuscript following the indicated order of the sections.

2nd Revision - Editorial Decision 20th Jul 2026

Dear Prof. Zippo,

I am pleased to inform you that your manuscript has been accepted for publication in the EMBO Journal.

Your manuscript will be processed for publication by EMBO Press. It will be copy edited and you will receive page proofs prior to publication. Please note that you will be contacted by Springer Nature Author Services to complete licensing and payment information.

You may qualify for financial assistance for your publication charges - either via a Springer Nature fully open access agreement or an EMBO initiative. Check your eligibility: https://link.springer.com/journal/44318/how-to-publish-with-us

Should you be planning a Press Release on your article, please notify the production team via this form in order to coordinate publication and release dates: https://forms.cloud.microsoft/e/eH4A8JVP6g

If you have any questions, please do not hesitate to contact the Editorial Office. Thank you for your contribution to The EMBO Journal.

Yours sincerely,

Cornelius Schneider, PhD
Editor
The EMBO Journal
c.schneider@embojournal.org

*****

Please note that it is The EMBO Journal policy for the transcript of the editorial process (containing referee reports and your response letters) to be published as an online supplement to each paper. If you should prefer removal of any referee-only figures included in the point-by-point response(s), e.g. because they may still be used for future publication or because they have been reproduced from published work by others, please do let us know immediately via response email.
More information is available here: https://link.springer.com/partners/embo-press/editorial-policies#Peer%20review

EMBO Press Author Checklist

Corresponding Author Name: Alessio Zippo	
Journal Submitted to: EMBO J	
Manuscript Number: EMBOJ-2025-122672	

USEFUL LINKS FOR COMPLETING THIS FORM
The EMBO Journal - Author Guidelines
EMBO Reports - Author Guidelines
Molecular Systems Biology - Author Guidelines
EMBO Molecular Medicine - Author Guidelines

Reporting Checklist for Life Science Articles (updated January
This checklist is adapted from Materials Design Analysis Reporting (MDAR) Checklist for Authors. MDAR establishes a minimum set of requirements in transparent reporting in the life sciences (see Statement of Task: 10.31222/osf.io/9sm4x). Please follow the journal's guidelines in preparing your
Please note that a copy of this checklist will be published alongside your article.

Abridged guidelines for figures
1. Data
The data shown in figures should satisfy the following conditions:
- the data were obtained and processed according to the field's best practice and are presented to reflect the results of the experiments in an accurate and unbiased manner.
- ideally, figure panels should include only measurements that are directly comparable to each other and obtained with the same assay.
- plots include clearly labeled error bars for independent experiments and sample sizes. Unless justified, error bars should not be shown for technical
- if n<5, the individual data points from each experiment should be plotted. Any statistical test employed should be justified.
- Source Data should be included to report the data underlying figures according to the guidelines set out in the authorship guidelines on Data

2. Captions
Each figure caption should contain the following information, for each panel where they are relevant:
- a specification of the experimental system investigated (eg cell line, species name).
- the assay(s) and method(s) used to carry out the reported observations and measurements.
- an explicit mention of the biological and chemical entity(ies) that are being measured.
- an explicit mention of the biological and chemical entity(ies) that are altered/varied/perturbed in a controlled manner.
- the exact sample size (n) for each experimental group/condition, given as a number, not a range;
- a description of the sample collection allowing the reader to understand whether the samples represent technical or biological replicates (including how many animals, litters, cultures, etc.).
- a statement of how many times the experiment shown was independently replicated in the laboratory.
- definitions of statistical methods and measures:
  - common tests, such as t-test (please specify whether paired vs. unpaired), simple χ2 tests, Wilcoxon and Mann-Whitney tests, can be unambiguously identified by name only, but more complex techniques should be described in the methods section;
  - are tests one-sided or two-sided?
  - are there adjustments for multiple comparisons?
  - exact statistical test results, e.g., P values = x but not P values < x;
  - definition of 'center values' as median or average;
  - definition of error bars as s.d. or s.e.m.

Please complete ALL of the questions below.
Select "Not Applicable" only when the requested information is not relevant for your study.

Materials

Newly Created Materials	Information included in the manuscript?	In which section is the information available? (Reagents and Tools Table, Materials and Methods, Figures, Data Availability Section)
New materials and reagents need to be available; do any restrictions apply?	Yes	Reagents and Tools Table, Material and Methods

Antibodies	Information included in the manuscript?	In which section is the information available? (Reagents and Tools Table, Materials and Methods, Figures, Data Availability Section)
For **antibodies** provide the following information:<br>- Commercial antibodies: RRID (if possible) or supplier name, catalogue number and or/clone number<br>- Non-commercial: RRID or citation	Yes	Reagents and Tools Table

DNA and RNA sequences	Information included in the manuscript?	In which section is the information available? (Reagents and Tools Table, Materials and Methods, Figures, Data Availability Section)
**Short novel DNA or RNA including primers, probes**: provide the sequences.	Yes	Reagents and Tools Table

Cell materials	Information included in the manuscript?	In which section is the information available? (Reagents and Tools Table, Materials and Methods, Figures, Data Availability Section)
**Cell lines:** Provide species information, strain. Provide accession number in repository **OR** supplier name, catalog number, clone number, and/**OR** RRID.	Yes	Materials and Methods
**Primary cultures:** Provide species, strain, sex of origin, genetic modification status.	Yes	Materials and Methods
Report if the cell lines were recently **authenticated** (e.g., by STR profiling) and tested for mycoplasma contamination.	Yes	Materials and Methods

Experimental animals	Information included in the manuscript?	In which section is the information available? (Reagents and Tools Table, Materials and Methods, Figures, Data Availability Section)
**Laboratory animals or Model organisms:** Provide species, strain, sex, age, genetic modification status. Provide accession number in repository **OR** supplier name, catalog number, clone number, **OR** RRID.	Yes	Reagents and Tools Table, Material and Methods
**Animal observed in or captured from the field:** Provide species, sex, and age where possible.	Not Applicable	
Please detail **housing and husbandry conditions**.	Yes	Materials and Methods

Plants and microbes	Information included in the manuscript?	In which section is the information available? (Reagents and Tools Table, Materials and Methods, Figures, Data Availability Section)
**Plants:** provide species and strain, ecotype and cultivar where relevant, unique accession number if available, and source (including location for collected wild specimens).	Not Applicable	
**Microbes:** provide species and strain, unique accession number if available, and source.	Not Applicable	

Human research participants	Information included in the manuscript?	In which section is the information available? (Reagents and Tools Table, Materials and Methods, Figures, Data Availability Section)
If collected and within the bounds of privacy constraints report on age, sex and gender or ethnicity for all study participants.	Not Applicable	

Core facilities	Information included in the manuscript?	In which section is the information available? (Reagents and Tools Table, Materials and Methods, Figures, Data Availability Section)
If your work benefited from core facilities, was their service mentioned in the acknowledgments section?	Yes	Acknowledgments

Design

Study protocol	Information included in the manuscript?	In which section is the information available? (Reagents and Tools Table, Materials and Methods, Figures, Data Availability Section)
If study protocol has been **pre-registered, provide DOI in the manuscript**. For clinical trials, provide the trial registration number **OR** cite DOI.	Not Applicable	
Report the **clinical trial registration number** (at ClinicalTrials.gov or equivalent), where applicable.	Not Applicable	

Laboratory protocol	Information included in the manuscript?	In which section is the information available? (Reagents and Tools Table, Materials and Methods, Figures, Data Availability Section)
Provide DOI OR other citation details if **external detailed step-by-step protocols** are available.	Not Applicable	

Experimental study design and statistics	Information included in the manuscript?	In which section is the information available? (Reagents and Tools Table, Materials and Methods, Figures, Data Availability Section)
Include a statement about **sample size** estimate even if no statistical methods were used.	Yes	Materials and Methods
Were any steps taken to minimize the effects of subjective bias when allocating animals/samples to treatment (e.g. **randomization procedure**)? If yes, have they been described?	Not Applicable	
Include a statement about **blinding** even if no blinding was done.	Yes	Materials and Methods
Describe **inclusion/exclusion criteria** if samples or animals were excluded from the analysis. Were the criteria pre-established?<br><br>If sample or data points were omitted from analysis, report if this was due to attrition or intentional exclusion and provide justification.	Not Applicable	
For every figure, are **statistical tests** justified as appropriate? Do the data meet the assumptions of the tests (e.g., normal distribution)? Describe any methods used to assess it. Is there an estimate of variation within each group of data? Is the variance similar between the groups that are being statistically compared?	Yes	Materials and Methods

Sample definition and in-laboratory replication	Information included in the manuscript?	In which section is the information available? (Reagents and Tools Table, Materials and Methods, Figures, Data Availability Section)
In the figure legends: state number of times the experiment was **replicated** in laboratory.	Yes	Figure legends
In the figure legends: define whether data describe **technical or biological replicates**.	Yes	Figure legends

Ethics

Ethics	Information included in the manuscript?	In which section is the information available? (Reagents and Tools Table, Materials and Methods, Figures, Data Availability Section)
Studies involving **human participants**: State details of **authority granting ethics approval** (IRB or equivalent committee(s), provide reference number for approval.	Not Applicable	
Studies involving **human participants**: Include a statement confirming that **informed consent** was obtained from all subjects and that the experiments conformed to the principles set out in the WMA Declaration of Helsinki and the Department of Health and Human Services Belmont Report.	Not Applicable	
Studies involving **human participants:** For publication of **patient photos**, include a statement confirming that consent to publish was obtained.	Not Applicable	
Studies involving experimental **animals**: State details of **authority granting ethics approval** (IRB or equivalent committee(s), provide reference number for approval. Include a statement of compliance with ethical regulations.	Yes	Materials and Methods
Studies involving **specimen and field samples:** State if relevant **permits** obtained, provide details of authority approving study; if none were required, explain why.	Not Applicable	

Dual Use Research of Concern (DURC)	Information included in the manuscript?	In which section is the information available? (Reagents and Tools Table, Materials and Methods, Figures, Data Availability Section)
Could your study fall under dual use research restrictions? Please check biosecurity documents and list of **select agents and toxins** (CDC): https://www.selectagents.gov/sat/list.htm	Not Applicable	
If you used a select agent, is the security level of the lab appropriate and reported in the manuscript?	Not Applicable	
If a study is subject to dual use research of concern regulations, is the name of the **authority granting approval and reference number** for the regulatory approval provided in the manuscript?	Not Applicable	

Reporting

The MDAR framework recommends adoption of discipline-specific guidelines, established and endorsed through community initiatives. Journals have their own policy about requiring specific guidelines and recommendations to complement MDAR.

Adherence to community standards	Information included in the manuscript?	In which section is the information available? (Reagents and Tools Table, Materials and Methods, Figures, Data Availability Section)
State if relevant guidelines or checklists (e.g., **ICMJE, MIBBI, ARRIVE, PRISMA**) have been followed or provided.	Yes	
For **tumor marker prognostic studies,** we recommend that you follow the **REMARK** reporting guidelines (see link list at top right). See author guidelines, under 'Reporting Guidelines'. Please confirm you have followed these guidelines.	Not Applicable	Materials and Methods
For **phase II and III randomized controlled trials**, please refer to the **CONSORT** flow diagram (see link list at top right) and submit the CONSORT checklist (see link list at top right) with your submission. See author guidelines, under 'Reporting Guidelines'. Please confirm you have submitted this list.	Not Applicable	

Data Availability

Data availability	Information included in the manuscript?	In which section is the information available? (Reagents and Tools Table, Materials and Methods, Figures, Data Availability Section)
Have **primary datasets** been deposited according to the journal's guidelines (see 'Data Deposition' section) and the respective accession numbers provided in the Data Availability Section?	Not Applicable	
Were **human clinical and genomic datasets** deposited in a public access-controlled repository in accordance to ethical obligations to the patients and to the applicable consent agreement?	Not Applicable	
Are **computational models** that are central and integral to a study available without restrictions in a machine-readable form? Were the relevant accession numbers or links provided?	Yes	Material and methods
If publicly available data were reused, provide the respective **data citations in the reference list**.	Not Applicable	
