# Supplementary material for: Chromatin condensates tune nuclear mechanosensing and preserve nuclear integrity to prevent cGAS activation
Source: EMBO J. 2026 Aug 12;45(18):6384–420. doi: 10.1038/s44318-026-00884-z (PMC13584762; doi:10.1038/s44318-026-00884-z)
Supplement: Supplementary file 3 [file 44318_2026_884_MOESM92_ESM.pdf]

Expanded View Figures

Figure EV1. Substrate stiffness unbalances the transcription- and PcG-associated chromatin condensates.

(**A**) Representative confocal images and relative quantification of nuclear MLL4 mean intensity in $MLL4^{WT}$ and $MLL4^{LoF}$ MSCs on soft and stiff matrix. Scale bars, 15 µm. Bar plots show mean + SEM of $n = 3$ independent biological replicates. Statistical significance was determined by Sidak's multiple comparison test (two-way ANOVA). (**B**) Quantifications of nuclear MLL4 mean intensity in $MLL4^{WT\_2}$ and $MLL4^{LoF\_2}$ MSCs, independent clones on stiff matrix. Bar plots show mean + SEM of $n = 3$ independent biological replicates. Statistical significance was determined by a two-tailed unpaired Student's *t*-test. (**C**) Real-time quantitative reverse transcription PCR of the indicated genes in $MLL4^{WT}$ and $MLL4^{LoF}$ MSCs on soft and stiff matrix (mean + SEM; $n = 3$ independent experiments each). (**D**) Condensates quantifications of immunostaining for BRD4 in $MLL4^{WT\_2}$ and $MLL4^{LoF\_2}$ MSCs, independent clones on soft and stiff matrix. Bar plots show mean + SEM of $n = 3$ independent biological replicates. Statistical significance was determined by Sidak's multiple comparison test (two-way ANOVA). (**E**) Condensates quantifications of immunostaining for BRD4 in primary fibroblasts from healthy donor (Ctrl) or Kabuki patients (Mut) on soft and stiff matrix. Bar plots show mean + SEM of $n = 3$ independent biological replicates. Statistical significance was determined by Sidak's multiple comparison test (two-way ANOVA). (**F**) Representative confocal images of EGFP signal and Phalloidin staining in $MLL4^{WT}$ MSCs expressing pEGFP C1 or pEGFP C1_DN_KASH construct. On the right, quantification of the Phalloidin Area. Bar plots show mean + SEM of $n = 3$ independent biological replicates. Statistical significance was determined by a two-tailed unpaired Student's *t*-test. (**G**) Quantifications of nuclear condensates for BRD4 in $MLL4^{WT}$ MSCs expressing pEGFP C1 or pEGFP C1_DN_KASH construct on soft and stiff matrix. Bar plots show mean + SEM of $n = 3$ independent biological replicates. Statistical significance was determined by Sidak's multiple comparison test (two-way ANOVA). Representative confocal images and relative quantifications of condensates for RING1B in $MLL4^{WT}$/ $MLL4^{LoF}$ (**H**) and $MLL4^{WT\_2}$/ $MLL4^{LoF\_2}$ independent clones (**I**) MSCs on soft and stiff matrix. Scale bars, 15 µm. Bar plots show mean + SEM of $n = 3$ independent biological replicates. Statistical significance was determined by Sidak's multiple comparison test (two-way ANOVA). (**J**) Quantifications of immunostaining for BMI nuclear condensates in $MLL4^{WT\_2}$ and $MLL4^{LoF\_2}$ MSCs, independent clones on soft and stiff matrix. Bar plots show mean + SEM of $n = 3$ independent biological replicates. Statistical significance was determined by Sidak's multiple comparison test (two-way ANOVA). (**K**) Quantifications of nuclear BMI condensates in primary fibroblasts from healthy donors (Ctrl) or Kabuki patients (Mut) on soft and stiff matrix. (**L**) Quantifications of nuclear condensates for BMI in $MLL4^{WT}$ MSCs expressing pEGFP C1 or pEGFP C1_DN_KASH construct on soft and stiff matrix. Bar plots show mean + SEM of $n = 3$ independent biological replicates. Statistical significance was determined by Sidak's multiple comparison test (two-way ANOVA). (**M–O**) Representative images and relative quantifications of nuclear condensates for BMI (**M**), BRD4 (**N**), and MED1 (**O**) in $MLL4^{WT}$ and $MLL4^{LoF}$ MSCs, as well as $MLL4^{LoF}$ MSCs expressing either H3.3WT or H3.3K27M on soft and stiff matrix. Bar plots show mean + SEM of $n = 3$ independent biological replicates. Scale bars, 15 µm. Statistical significance was determined by Sidak's multiple comparison test (two-way ANOVA). *P* values definition: $*p < 0.05$, $**p < 0.01$, $***p < 0.001$, $****p < 0.0001$, ns not significant ($p \geq 0.05$).

© The Author(s)

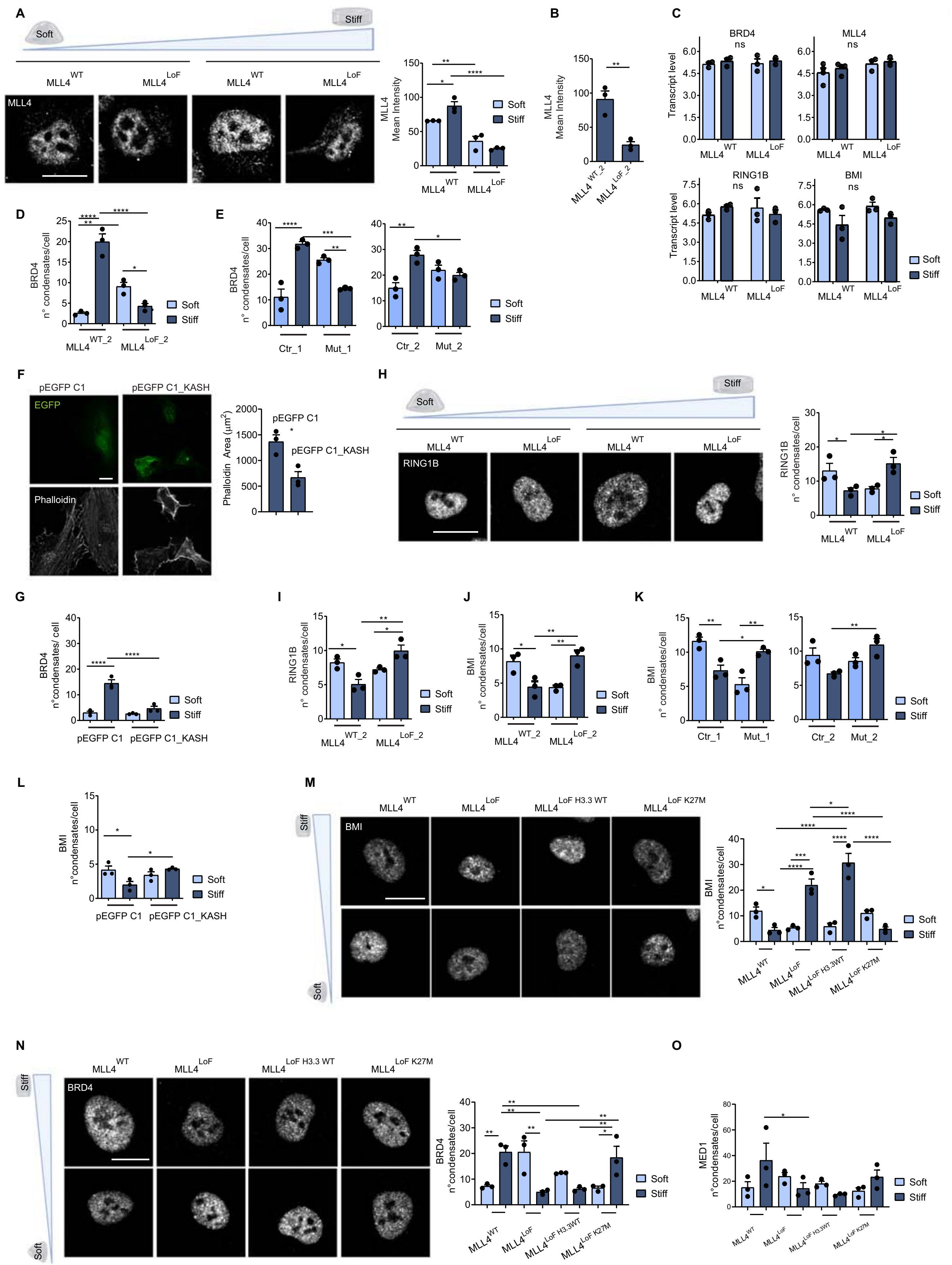
A
Soft
Stiff
MLL4 WT
MLL4 LoF
MLL4
MLL4 Mean Intensity
Soft
Stiff
B
MLL4 Mean Intensity
MLL4 WT_2
MLL4 LoF_2
C
BRD4
MLL4
RING1B
BMI
ns
Transcript level
D
BRD4 n° condensates/cell
MLL4 WT_2
MLL4 LoF_2
E
BRD4 n° condensates/cell
Ctr_1
Mut_1
Ctr_2
Mut_2
F
pEGFP C1
pEGFP C1_KASH
EGFP
Phalloidin
Phalloidin Area (μm²)
H
RING1B
RING1B n° condensates/cell
G
BRD4 n°condensates/ cell
I
RING1B n° condensates/cell
J
BMI n° condensates/cell
K
BMI n°condensates/cell
L
BMI n°condensates/cell
M
MLL4 LoF H3.3 WT
MLL4 LoF K27M
BMI
BMI n°condensates/cell
N
BRD4
BRD4 n°condensates/cell
O
MED1 n°condensates/cell

 © The Author(s)

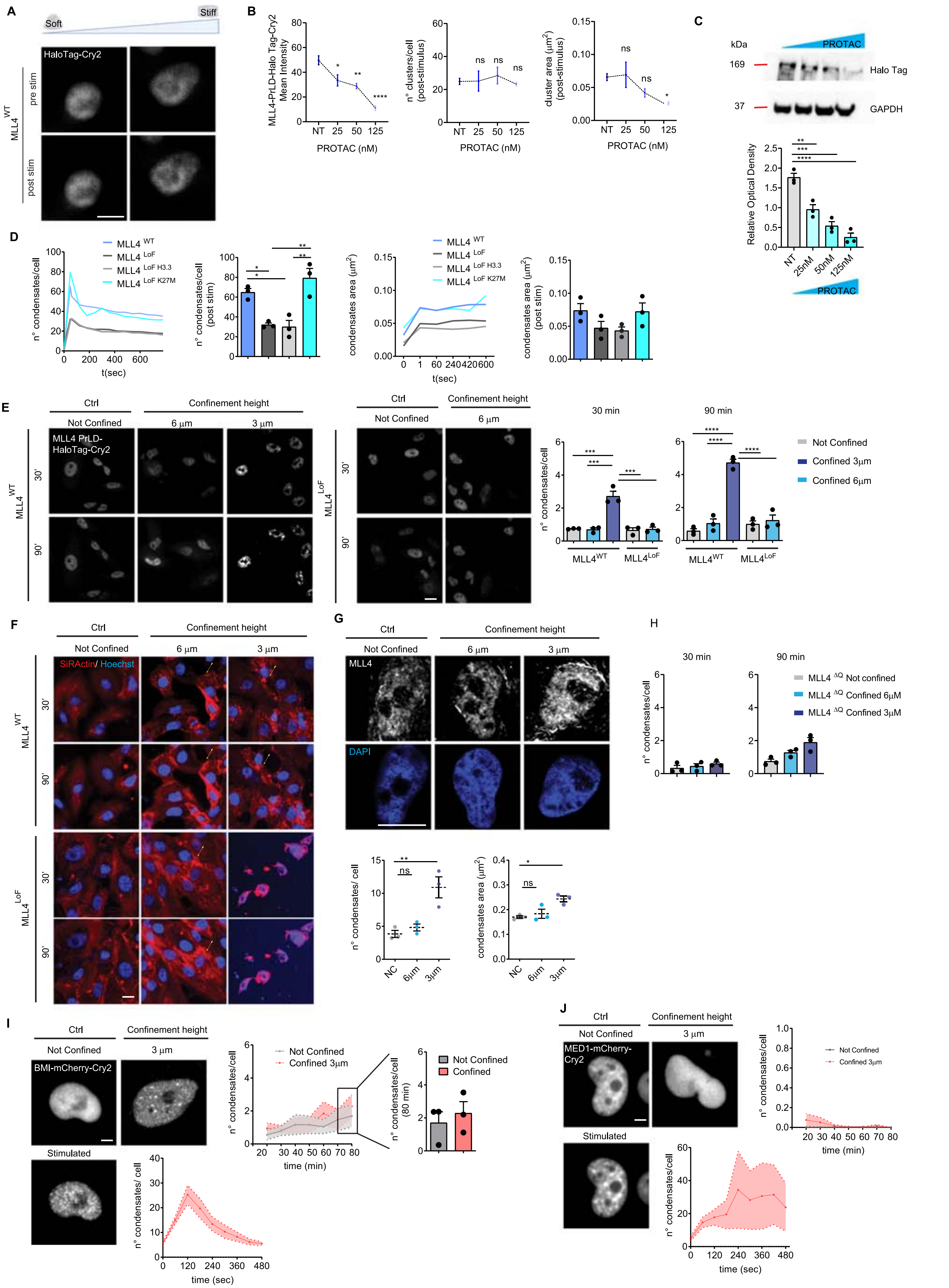
A
Soft
Stiff
HaloTag-Cry2
MLL4 WT
pre stim
post stim
B
MLL4-PrLD-Halo Tag-Cry2 Mean Intensity
PROTAC (nM)
n° clusters/cell (post-stimulus)
cluster area (μm²) (post-stimulus)
NT 25 50 125
C
kDa
PROTAC
169
Halo Tag
37
GAPDH
Relative Optical Density
NT 25nM 50nM 125nM
D
MLL4 WT
MLL4 LoF
MLL4 LoF H3.3
MLL4 LoF K27M
n° condensates/cell
t(sec)
n° condensates/cell (post stim)
condensates area (μm²)
condensates area (μm²) (post stim)
E
Ctrl
Confinement height
Not Confined
6 μm
3 μm
MLL4 PrLD-HaloTag-Cry2
30'
90'
MLL4 WT
MLL4 LoF
30 min
90 min
Not Confined
Confined 3μm
Confined 6μm
F
SiRActin/ Hoechst
G
MLL4
DAPI
n° condensates/ cell
condensates area (μm²)
NC 6μm 3μm
H
MLL4 ΔQ Not confined
MLL4 ΔQ Confined 6μM
MLL4 ΔQ Confined 3μM
I
BMI-mCherry-Cry2
Stimulated
Not Confined
Confined 3μm
n° condensates/cell (80 min)
Confined
time (min)
time (sec)
J
MED1-mCherry-Cry2

Figure EV2. MLL4 condensates respond to external mechanical forces.

(**A**) Representative images of MSCs on soft and stiff matrix overexpressing HaloTag-Cry2 before and after the stimulus with blue light. Scale bars, 10 µm. (**B**) Quantification of the number and area of light-induced droplets of MLL4-PrLD-HaloTag-Cry2 in $MLL4^{WT}$ MSCs after the blue light stimulus treated with PROTAC3 at the concentration indicated (NT nontreated). Bar plots show mean + SEM of $n = 3$ independent biological replicates. Statistical significance was determined by Dunnett's multiple comparison test (Welch and Brown–Forsythe one-way ANOVA). (**C**) Western Blot analysis of MLL4-PrLD-HaloTag-Cry2 in $MLL4^{WT}$ MSCs after PROTAC3 treatment ($n = 3$ independent experiments). Bar plots show mean + SEM of $n = 3$ independent biological replicates. Statistical significance was determined by Dunnett's multiple comparison test (one-way ANOVA). (**D**) Quantification of the number and area of light-induced droplets of MLL4-PrLD-HaloTag-Cry2 at different time points in $MLL4^{WT}$ and $MLL4^{LoF}$ MSCs, as well as $MLL4^{LoF}$ MSCs expressing either H3.3WT or H3.3K27M on soft and stiff matrix. The time point $t = 0$ represents the pre-stimulus. Bar plots show quantifications of n° condensates/cell in the post-stimulus (mean + SEM of $n = 3$ independent biological replicates). Statistical significance was determined by Sidak's multiple comparison test (two-way ANOVA). (**E**) Representative image of $MLL4^{WT}$ and $MLL4^{LoF}$ MSCs overexpressing MLL4-PrLD-HaloTag-Cry2 not confined or confined at 6 and 3 µm in adhesion for 30 and 90 min. Scale bars, 15 µm. On the right, quantification of the number of condensates/cell at the indicated time points. (mean + SEM of $n = 3$ independent biological replicates). Statistical significance was determined by Sidak's multiple comparison test (two-way ANOVA). (**F**) Representative images showing adherent $MLL4^{WT}$ and $MLL4^{LoF}$ MSCs unconfined, or confinement at 6 and 3 µm, at the indicated time points. Cell nuclei are stained with Hoechst (blue), whether the cytoskeleton is marked in red (SirActin). Yellow arrows indicate actomyosin recruitment to the cell-cortex in response to confinement. Scale bars, 15 µm. (**G**) Representative confocal images and relative quantifications of immunostaining for MLL4 in $MLL4^{WT}$ MSCs unconfined and confined at 6 and 3 µm for 45 min. Scale bars, 15 µm. Bar plots show mean + SEM of $n = 3$ independent biological replicates. Statistical significance was determined by Dunnett's multiple comparison test (one-way ANOVA). (**H**) Quantification of the number of condensates/cell in $MLL4^{WT}$ MSCs expressing MLL4-PrLD-ΔQ-HaloTag-Cry2 confined in adhesion at 6 and 3 µm for 30 and 90 min. Bar plots show mean + SEM of $n = 3$ independent biological replicates. Statistical significance was determined by Sidak's multiple comparison test (two-way ANOVA). (**I**, **J**) Representative images of $MLL4^{WT}$ MSCs expressing BMI-mCherry-Cry2 (**I**) and MED1-mCherry-Cry2 (**J**) in not confined cells +/− blue light stimulation and confined at 3 µm. On the right, quantification of the number of condensates at different time points is shown upon confinement and upon stimulation. Bar plots show mean + SEM of $n = 3$ independent biological replicates; two-tailed unpaired Student's *t*-test. Scale bars, 5 µm. *P* values definition: $^{*}p < 0.05$, $^{**}p < 0.01$, $^{***}p < 0.001$, $^{****}p < 0.0001$, ns not significant ($p \geq 0.05$).

 © The Author(s)

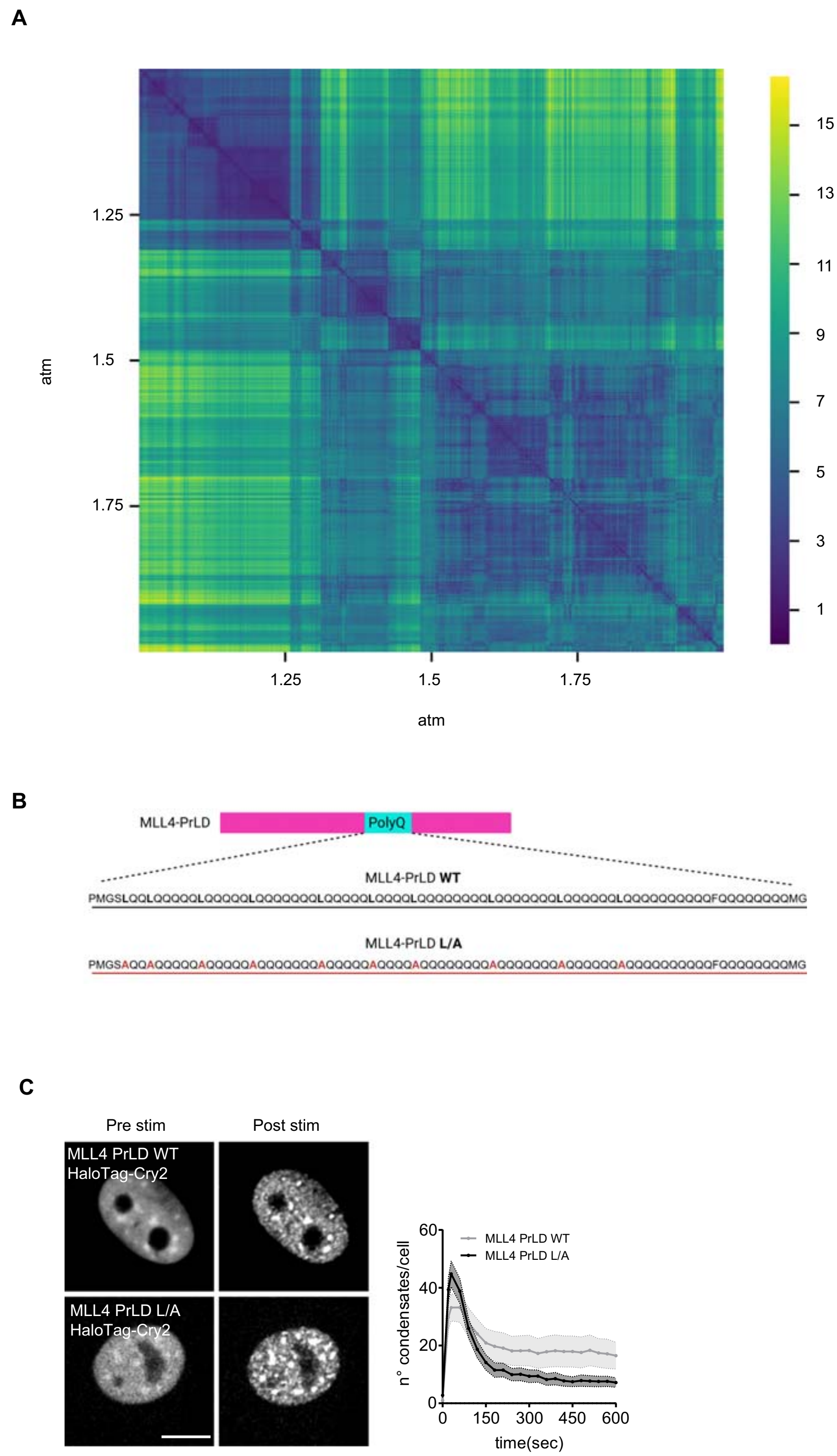

Figure EV3. Mechanical forces determine the folding transition of the polyQ stretch of MLL4-PrLD.

(**A**) Two-dimensional root mean square displacement (RMSD) profile of the polyQ tract of MLL4-PrLD, based upon the coordinates of the two most stable helices along the MD trajectory (i.e., conserved in over 75% of the trajectory frames and involving residues 3928–3935 and residues 3948–3954). A low value of the RMSD is associated with a high degree of structural/conformational similarity between frames: Here, two broad squares (blue shades) might be identified that corroborate the existence of a twofold conformational basin of the polyQ tract, the higher pressure regimes stabilizing a more expanded configuration. Analysis performed within the MD Analysis framework (PMID: 15973002, 21500218). (**B**) Schematic representation of MLL4 PrLD L/A substitutions within the PolyQ stretch. (**C**) Representative images of MSCs overexpressing MLL4-PrLD WT-/ MLL4-PrLD L/A-HaloTag-Cry2 before and after the stimulus with blue light. On the right, quantifications of n° condensates/cell (merge of two biological replicates). Scale bars, 10 µm. *P* values definition: **p* < 0.05, ***p* < 0.01, ****p* < 0.001, *****p* < 0.0001, ns not significant (*p* ≥ 0.05).

© The Author(s)

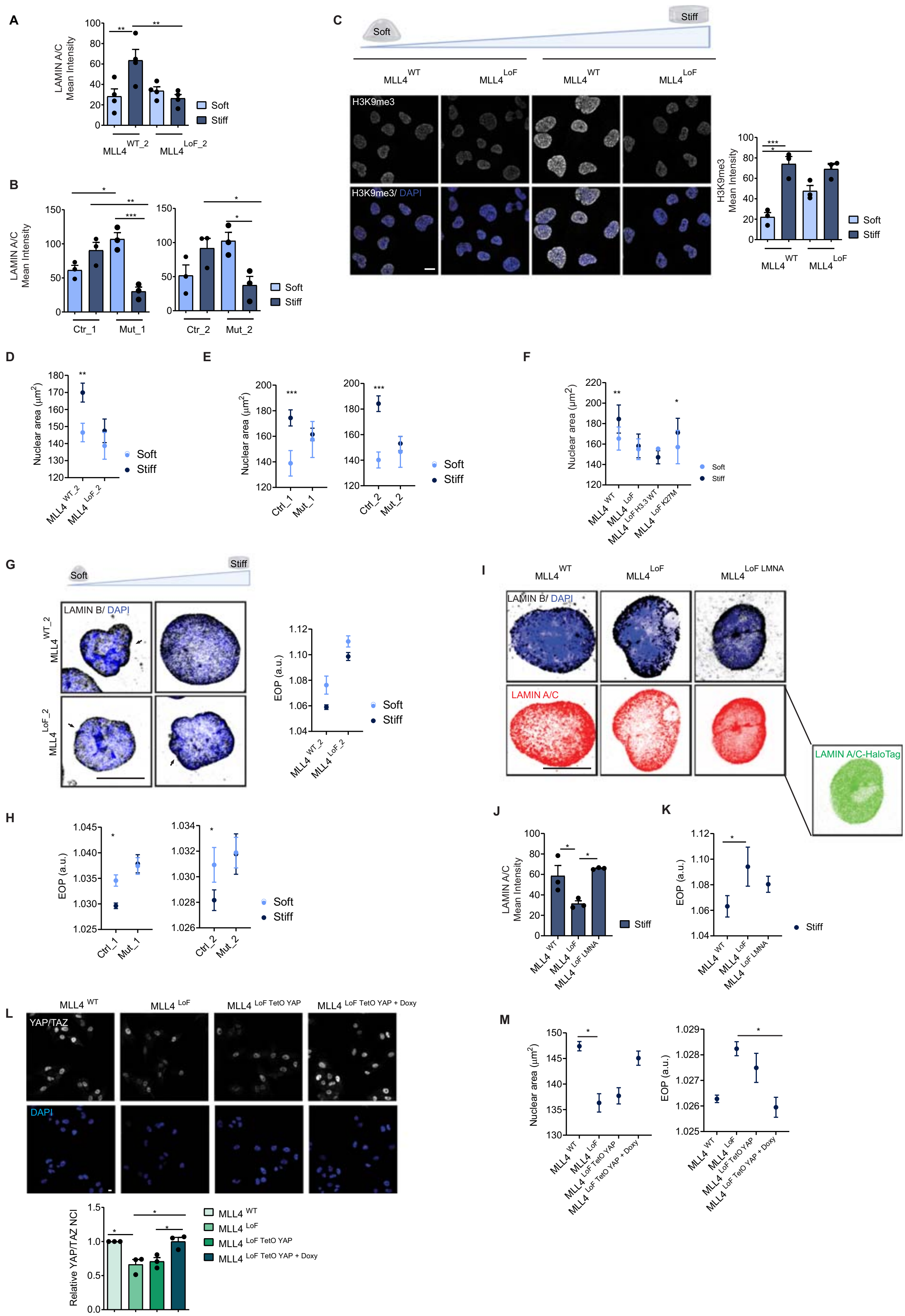
A
B
C
Soft
Stiff
H3K9me3
H3K9me3/ DAPI
D
E
F
G
LAMIN B/ DAPI
H
I
LAMIN A/C
LAMIN A/C-HaloTag
J
K
L
YAP/TAZ
DAPI
M
Nuclear area (µm²)
EOP (a.u.)
Relative YAP/TAZ NCI

◀ **Figure EV4. Unbalancing chromatin condensates affects nuclear envelope tension in response to substrate stiffness.**

(**A**) Quantifications of immunostaining for LAMIN A/C in $MLL4^{WT\_2}$ and $MLL4^{LoF\_2}$ MSCs, independent clones on soft and stiff matrix. Bar plots show mean + SEM of $n = 4$ independent biological replicates. Statistical significance was determined by Sidak's multiple comparison test (two-way ANOVA). (**B**) Quantifications of nuclear LAMIN A/C mean intensity in primary fibroblasts from healthy donors (Ctrl) or Kabuki patients (Mut) on soft and stiff matrix. Bar plots show mean + SEM of $n = 3$ independent biological replicates. Statistical significance was determined by Sidak's multiple comparison test (two-way ANOVA). (**C**) Representative confocal images and relative quantifications of immunostaining for H3K9me3 in $MLL4^{WT}$ and $MLL4^{LoF}$ MSCs on soft and stiff matrix. Scale bars, 10 µm. Bar plots show mean + SEM of $n = 3$ independent biological replicates. Statistical significance was determined by Sidak's multiple comparison test (two-way ANOVA). Quantifications of nuclear area in $MLL4^{WT}$ and $MLL4^{LoF}$ MSCs independent clones (**D**) and in primary fibroblasts (**E**) on soft and stiff matrix. Bar plots show mean + SEM of $n \geq 3$ independent biological replicates. Statistical significance was determined by a linear mixed-effects model. (**F**) Quantifications of the nuclear area in MLL4WT and MLL4LoF MSCs and MLL4LoF MSCs expressing either H3.3WT or H3.3K27M on soft and stiff matrix. Bar plots show mean + SEM of $n \geq 3$ independent biological replicates. Statistical significance was determined by a linear mixed-effects model. Representative confocal images and relative quantification of immunostaining for LAMIN B in $MLL4^{WT}$ and $MLL4^{LoF}$ MSCs independent clones (**G**) and primary fibroblasts (**H**) on soft and stiff matrix. Black arrows indicate the presence of nuclear envelope invaginations, quantified as EOP (excess of perimeter). Scale bars, 10 µm. Bar plots show mean + SEM of $n = 3$ independent biological replicates. Statistical significance was determined by a linear mixed-effects model. (**I**) Representative confocal images of immunostaining for LAMIN B and LAMIN A/C in $MLL4^{WT}$ and $MLL4^{LoF}$ MSCs, as well as $MLL4^{LoF}$ MSCs expressing Halo-tag LAMIN A/C. Bar plots (mean + SEM) showing quantification of LAMIN A/C (**J**) and EOP (**K**) in $MLL4^{WT}$, $MLL4^{LoF}$ and $MLL4^{LoF}$ expressing Halo-tag LAMIN A/C MSCs. Scale bars, 10 µm. $N \geq 3$ independent biological replicates. Statistical significance was determined by Sidak's multiple comparison test (two-way ANOVA) for pane (**J**) and linear mixed-effects model for panel (**K**). (**L**) Representative confocal images of immunostaining for YAP/TAZ in $MLL4^{WT}$, $MLL4^{LoF}$, and $MLL4^{LoF}$ MSCs overexpressing TetO YAP (±24 h doxycycline treatment) on stiff matrix. Scale bars, 10 µm. Below, quantification of the relative nuclear to cytoplasmic ratio (NCI). Bar plots show mean + SEM of $n = 3$ independent biological replicates. Statistical significance was determined by Tukey's multiple comparison test (two-way ANOVA). (**M**) Quantification of nuclear area and nuclear envelope invaginations (EOP, based on LAMIN B immunostaining) in $MLL4^{WT}$, $MLL4^{LoF}$, and $MLL4^{LoF}$ MSCs overexpressing TetO YAP (±24 h doxycycline treatment) on stiff matrix. Mean of ≥3 independent biological replicates is plotted. *P* values definition: $^{*}p < 0.05$, $^{**}p < 0.01$, $^{***}p < 0.001$, $^{****}p < 0.0001$, ns not significant ($p \geq 0.05$).

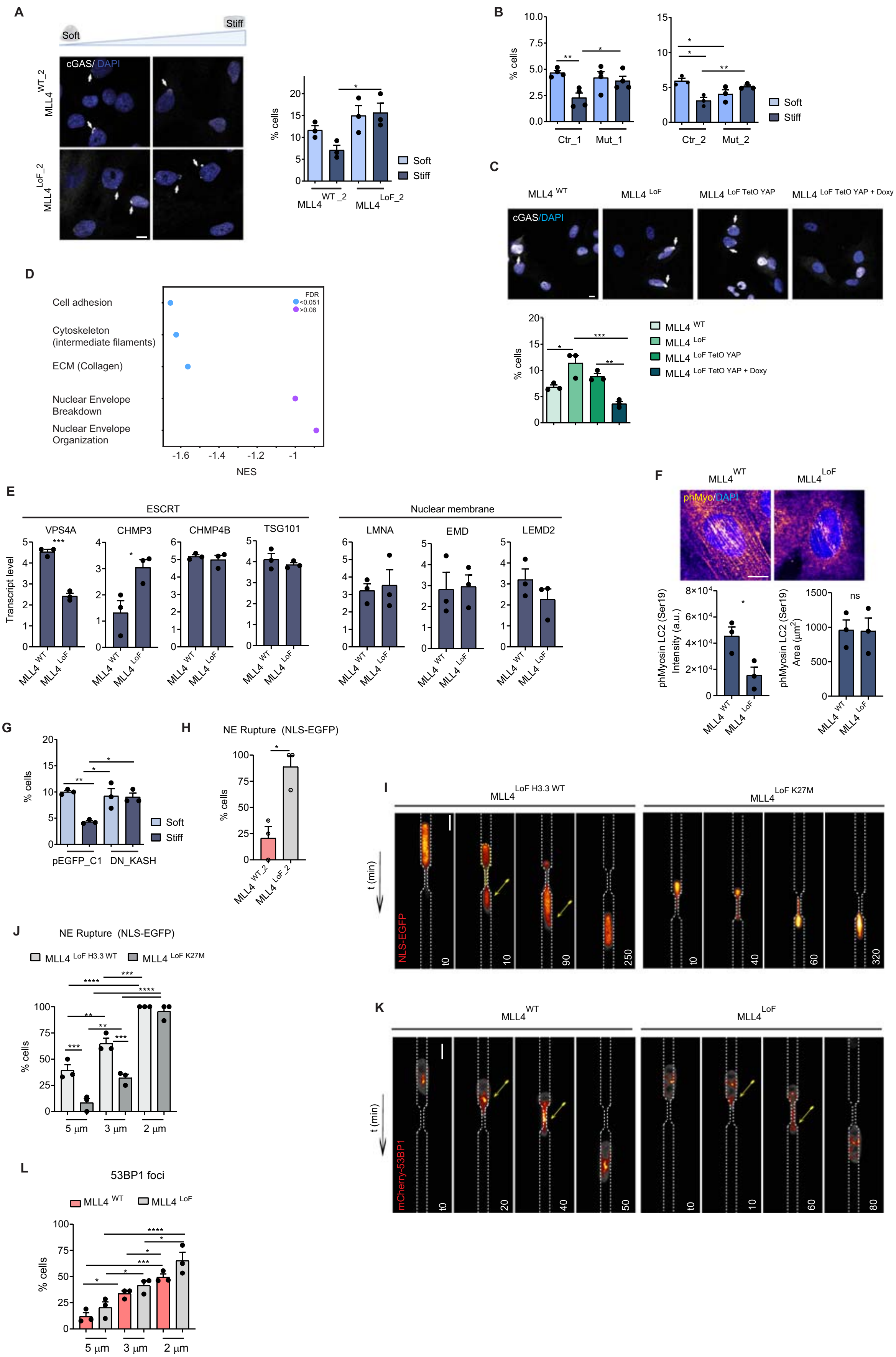
A
Soft
Stiff
cGAS/DAPI
MLL4 WT_2
MLL4 LoF_2
% cells
Soft
Stiff
B
% cells
Ctr_1
Mut_1
Ctr_2
Mut_2
Soft
Stiff
C
MLL4 WT
MLL4 LoF
MLL4 LoF TetO YAP
MLL4 LoF TetO YAP + Doxy
D
Cell adhesion
Cytoskeleton (intermediate filaments)
ECM (Collagen)
Nuclear Envelope Breakdown
Nuclear Envelope Organization
FDR
<0.051
>0.08
NES
E
ESCRT
Nuclear membrane
VPS4A
CHMP3
CHMP4B
TSG101
LMNA
EMD
LEMD2
Transcript level
F
phMyo/DAPI
phMyosin LC2 (Ser19) Intensity (a.u.)
phMyosin LC2 (Ser19) Area (μm²)
ns
G
pEGFP_C1
DN_KASH
H
NE Rupture (NLS-EGFP)
I
MLL4 LoF H3.3 WT
MLL4 LoF K27M
t (min)
NLS-EGFP
J
NE Rupture (NLS-EGFP)
5 μm
3 μm
2 μm
K
mCherry-53BP1
L
53BP1 foci

Figure EV5. MLL4 LoF leads to recurrent NE rupture upon mechanical stress.

(**A**) Representative confocal images of immunostaining for cGAS in $MLL4^{WT\_2}$ and $MLL4^{LoF\_2}$ MSCs independent clones on soft and stiff matrix. Scale bars, 10 μm. On the right, quantification of the percentage of cells showing cGAS accumulation at the nuclear periphery, indicating NE rupture (white arrows). Bar plots show mean + SEM of $n = 3$ independent biological replicates. Statistical significance was determined by Sidak's multiple comparison test (two-way ANOVA). (**B**) Quantification of the percentage of cells showing cGAS accumulation at the nuclear periphery in primary fibroblasts from healthy donors (Ctrl) or Kabuki patients (Mut) on soft and stiff matrix, indicating NE rupture (white arrows). Bar plots show mean + SEM of $n = 3$ independent biological replicates. Statistical significance was determined by Sidak's multiple comparison test (two-way ANOVA). (**C**) Representative confocal images of immunostaining for cGAS in $MLL4^{WT}$, $MLL4^{LoF}$, and $MLL4^{LoF}$ MSCs overexpressing TetO YAP (±24 h doxycycline treatment) on stiff matrix. Scale bars, 10 μm. Below, quantification of % of cells showing cGAS accumulation at the nuclear periphery (white arrows). Bar plots show mean + SEM of $n = 3$ independent biological replicates. Statistical significance was determined by Tukey's multiple comparison test (two-way ANOVA). (**D**) Bubble plot showing the normalized enrichment score (NES) for the specified FDR (false discovery rate) in the selected categories (GSEA analysis). (**E**) Real-time quantitative reverse transcription PCR of the indicated genes implicated in NE organization and repair in $MLL4^{WT}$ and $MLL4^{LoF}$ MSCs on stiff matrix (mean + SEM; $n = 3$ independent experiments each). Statistical significance was determined by a two-tailed unpaired Student's *t*-test. (**F**) Representative confocal images of immunostaining for phMyosin Light Chain 2 (Ser19) in $MLL4^{WT}$ and $MLL4^{LoF}$ MSCs on soft stiff matrix. Scale bars, 10 μm. Below, quantification of phMyosin intensity and area (mean + SEM; $n = 3$ independent experiments). Statistical significance was determined by a two-tailed unpaired Student's *t*-test. (**G**) Quantification of the percentage of cells showing cGAS accumulation at the nuclear periphery in $MLL4^{WT}$ MSCs expressing pEGFP C1 or pEGFP C1 DN_KASH construct on soft and stiff matrix. Bar plots show mean + SEM of $n = 3$ independent biological replicates. Statistical significance was determined by Sidak's multiple comparison test (two-way ANOVA). (**H**) Quantification of the percentage of cells undergoing NE rupture in NLS EGFP- MSCs (independent clones). Bar plots show mean + SEM of $n = 3$ independent biological replicates. Statistical significance was determined by a two-tailed unpaired Student's *t*-test. (**I**) Representative images of $MLL4^{LoF}$ H3.3WT and H3.3 K27M - NLS-EGFP in the process of passing through restrictions of 3 μm at the indicated time points. Scale bars, 10 μm. Dashed white lines highlight the microchannel borders. Yellow arrows indicate cells undergoing NE rupture. (**J**) Bar plots showing the percentage of cells undergoing NE rupture passing through constrictions of different widths (mean + SEM, $n = 3$). Statistical significance was determined by Sidak's multiple comparison test (two-way ANOVA). (**K**) Representative images of $MLL4^{WT}$ and $MLL4^{LoF}$ MSCs expressing mCherry-53BP1 in the process of passing through restrictions of 3 μm at the indicated time points. Scale bars, 10 μm. Dashed white lines highlight the microchannel borders. Yellow arrows highlight cells with 53BP1 foci while passing through the constriction. (**L**) Quantification of the percentage of cells harboring 53BP1 foci ($n = 3$ independent experiments) passing through constrictions of different widths (5, 3, and 2 μm). Bar plots show mean + SEM. Statistical significance was determined by Sidak's multiple comparison test (two-way ANOVA). *P* values definition: *$p < 0.05$, **$p < 0.01$, ***$p < 0.001$, ****$p < 0.0001$, ns not significant ($p \geq 0.05$).

© The Author(s)

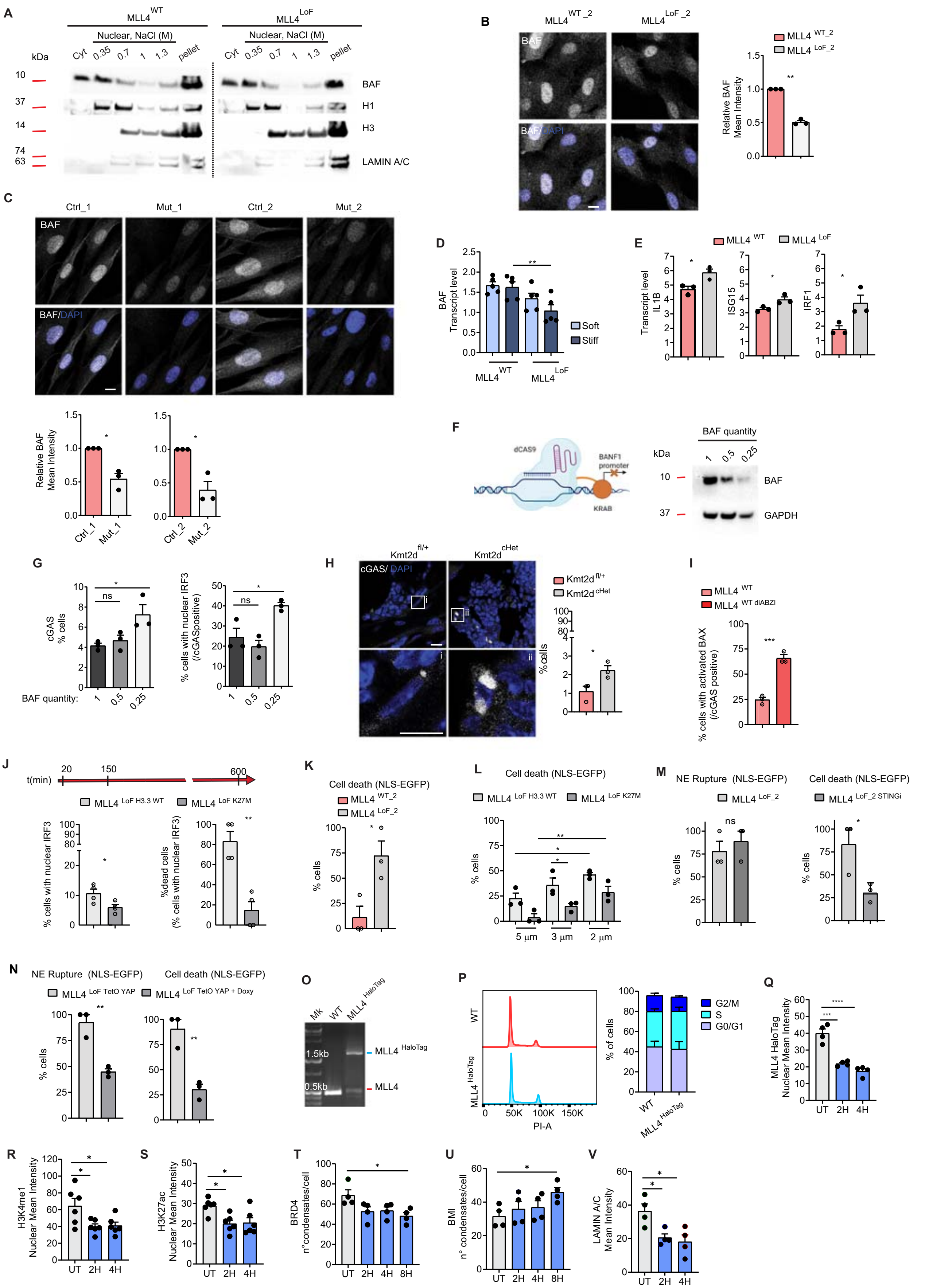

 © The Author(s)

Figure EV6. Disrupted NE integrity guides the cGAS/STING-mediated cell death in KS.

(**A**) Western Blot analysis of BAF, Histone H1, Histone H3, and LAMIN A/C in $MLL4^{WT}$ and $MLL4^{LoF}$ MSCs extracts obtained using a gradient of NaCl. (**B**) Representative confocal images and e quantifications of nuclear BAF mean intensity in $MLL4^{WT\_2}$ and $MLL4^{LoF\_2}$ MSCs on the stiff matrix. Bar plots represent data quantification as mean + SEM of three independent experiments. Statistical significance was determined by a two-tailed paired Student's *t*-test. Scale bar, 10 µm. (**C**) Representative confocal images and relative quantifications of immunostaining for BAF in primary fibroblasts from healthy donors (Ctrl) or Kabuki patients (Mut) on the stiff matrix. Bar plots represent data quantification as mean + SEM of three independent experiments. Statistical significance was determined by a two-tailed paired Student's *t*-test. Scale bar, 10 µm. (**D**) Real-time quantitative reverse transcription PCR of BAF in $MLL4^{WT}$ and $MLL4^{LoF}$ MSCs on soft and stiff matrix. Bar plots show mean + SEM of $n = 5$ biological replicates. Statistical significance was determined by Sidak's multiple comparison test (two-way ANOVA). (**E**) Real-time quantitative reverse transcription PCR of IL1B, ISG15 and IRF1 in $MLL4^{WT}$ and $MLL4^{LoF}$ MSCs on stiff matrix ($n = 3$ independent experiments each). Bar plots show mean + SEM of $n = 3$ independent replicates. Statistical significance was determined by a two-tailed unpaired Student's *t*-test. (**F**) Left: schematic representation of the CRISPRi strategy adopted. The sgRNAs are designed to target the BANF1 promoter. Right: Western Blot analysis of BAF in MLL4WT MSCs transduced at different MOI of sgRNA targeting BANF1 promoter to obtain a gradient of BAF quantity (1 = SCRAMBLE, 0.5 = MOI 1, 0.25 = MOI 0.25). (**G**) Quantification of the percentage of cells showing cGAS accumulation at the nuclear periphery and harboring IRF3 nuclear localization (calculated over the cGAS-positive cells) in $MLL4^{WT}$ expressing different levels of BAF protein. Bar plots show mean + SEM. of $n = 3$ biological replicates. Statistical significance was determined by Dunnett's multiple comparison test (one-way ANOVA). (**H**) Representative confocal images of immunostaining for cGAS in the primary spongiosa of $Kmt2d^{fl/+}$ and $Kmt2d^{cHet}$ mice. Scale bar, 10 µm. On the right, quantification of the percentage of cells showing cGAS accumulation at the nuclear periphery. Bar plots show mean + SEM of data combined from three experimental groups. Statistical significance was determined by a two-tailed unpaired Student's *t*-test. (**I**) Quantification of the percentage of cells with activated BAX measured over the cGAS-positive cells in $MLL4^{WT}$ MSCs +/− treated with diABZI. Bar plots show mean + SEM of $n = 3$ independent experiments. Statistical significance was determined by a two-tailed unpaired Student's *t*-test. (**J**) Quantification of the percentage of $MLL4^{LoF}$ H3.3WT and H3.3 K27M- IRF3-EGFP harboring IRF3 nuclear localization in the first 150′ of time-lapse (6µm confinement). On those cells having nuclear IRF3 was calculated the percentage of cells that undergo cell death within 600′ of time-lapse (Bar plots = mean + SEM of $n = 4$ combined from two independent experiments; two-tailed unpaired student's *t*-test. (**K**) Quantification of the percentage of cells undergoing cell death in NLS EGFP-MSCs independent clones passing through restrictions of 3µm. Bar plots show mean + SEM of $n = 3$ independent experiments. Statistical significance was determined by a two-tailed unpaired Student's *t*-test. (**L**) Quantification of the percentage of cells undergoing cell death in $MLL4^{LoF}$ H3.3WT and H3.3 K27M - NLS-EGFP passing through restrictions of 3 µm. Bar plots show mean + SEM. of $n = 3$ biological replicates. Statistical significance was determined by Sidak's multiple comparison test (two-way ANOVA). (**M**) Quantification of the percentage of cells undergoing NE rupture and cell death in NLS EGFP- $MLL4^{LoF}$ MSCs treated or not with the STINGi passing through restrictions of 3µm. Bar plots show mean + SEM of $n = 3$ independent experiments. Statistical significance was determined by a two-tailed unpaired Student's *t*-test. (**N**) Quantification of the percentage of cells undergoing NE rupture and cell death in NLS EGFP- $MLL4^{LoF}$ MSCs overexpressing TetO YAP (±24 h doxycycline treatment) passing through restrictions of 3 µm. Bar plots show mean + SEM of $n = 3$ independent experiments. Statistical significance was determined by a two-tailed unpaired Student's *t*-test. (**O**) Analyses of genomic DNA to confirm the HaloTag insertion in MLL4 HaloTag MSCs. PCR products were run in 1% agarose gel to separate upper and lower bands corresponding to edited (2291 bp) and parental templates (600 bp), respectively. The PCR strategy established HaloTag insertion (1691 bp) in hemizygosity at the desired site after the knock-in (right part of the gel). (**P**) Representative histograms of cell cycle analysis (PI-A) and relative quantification of cell % of cell cycle phases in WT and MLL4 HaloTag MSCs (mean + SEM of $n = 3$ biological replicates). Bar plot represents mean + SEM of $n > 4$ biological replicates of nuclear mean intensity of the endogenous MLL4 HaloTag (**Q**), H3K4me1 (**R**) H3K27ac (**S**), LAMIN A/C (**V**) retrieved in untreated or treated $MSCs^{HaloTag}$ with PROTAC3 for 2 and 4 h. Statistical significance was determined by a two-tailed unpaired Student's *t*-test. Bar plot (Mean + SEM of $N = 4$ biological replicates) showing quantification of BRD4 (**T**) and BMI (**U**) number of condensates/cell retrieved in untreated or treated $MSCs^{HaloTag}$ with PROTAC3 for 2, 4, and 8 h. Statistical significance was determined by a two-tailed unpaired Student's *t*-test. *P* values definition: $*p < 0.05$, $**p < 0.01$, $***p < 0.001$, $****p < 0.0001$, ns not significant ($p \geq 0.05$).

© The Author(s)
