# Supplementary material for: Chromatin condensates tune nuclear mechanosensing and preserve nuclear integrity to prevent cGAS activation
Source: EMBO J. 2026 Aug 12;45(18):6384–420. doi: 10.1038/s44318-026-00884-z (PMC13584762; doi:10.1038/s44318-026-00884-z)
Supplement: Supplementary file 5 [file 44318_2026_884_MOESM11_ESM.zip › Movie EV9/Movie EV9.docx]

**Movie EV9**

Time-lapse of MLL4^LoF^ MSCs over-expressing the NLS-EGFP reporter passing through a constriction of 2 μm. Events of cell death are visible. The constriction inside the channel was highlighted with two parallel white lines. One frame was taken every 10 minutes. Frame rate 4 fps. Scale bar 10 μm.
