# Supplementary material for: Chromatin condensates tune nuclear mechanosensing and preserve nuclear integrity to prevent cGAS activation
Source: EMBO J. 2026 Aug 12;45(18):6384–420. doi: 10.1038/s44318-026-00884-z (PMC13584762; doi:10.1038/s44318-026-00884-z)
Supplement: Supplementary file 34 [file 44318_2026_884_MOESM3_ESM.zip › Movie EV1/Movie EV1.docx]

**Movie EV1**

Time-lapse of MLL4^WT^ MSCs over-expressing MLL4-PrLD-HaloTag-Cry2 confined at 3μm. One frame was taken every 10 minutes. Frame rate 4 fps. Scale bar 10 μm.
