# Supplementary material for: Chromatin condensates tune nuclear mechanosensing and preserve nuclear integrity to prevent cGAS activation
Source: EMBO J. 2026 Aug 12;45(18):6384–420. doi: 10.1038/s44318-026-00884-z (PMC13584762; doi:10.1038/s44318-026-00884-z)
Supplement: Supplementary file 87 [file 44318_2026_884_MOESM88_ESM.zip › Fig.7a-c/7a.zip!/7a/WB_BAF_Uncropped_Replicates_Fig7a.pdf]

Replicate 1

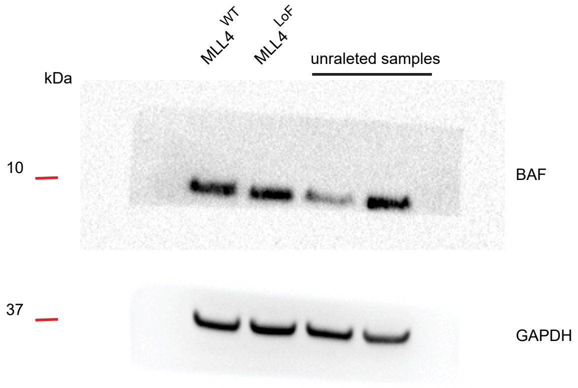

Replicate 2

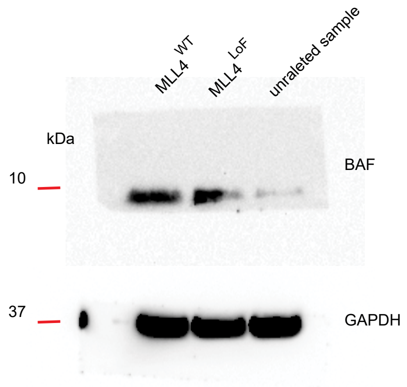

Replicate 3

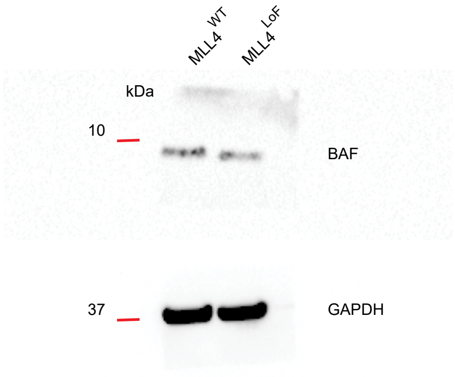

Replicate 4 (shown)

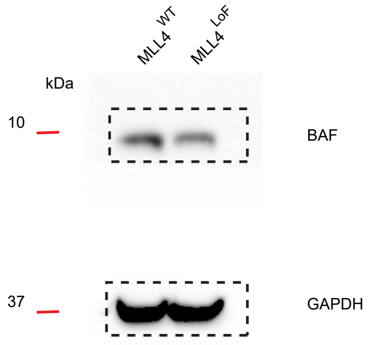

D’Annunzio_SourceData_Figure 7_a
